# Construction of Lymph Node Metastasis-Related Prognostic Model and Analysis of Immune Infiltration Mode in Lung Adenocarcinoma

**DOI:** 10.1155/2022/3887857

**Published:** 2022-06-29

**Authors:** Wujin Li, Debin Ou, Jiguang Zhang, Mingfan Ye

**Affiliations:** Thoracic Surgery Department, Fujian Provincial Hospital, Fuzhou 350001, China

## Abstract

**Background:**

Lung adenocarcinoma (LUAD) is a major cause for global cancer-related deaths. Research reports demonstrate that lymph node metastasis (LNM) is pertinent to the survival rate of LUAD patients, and crux lies in the lack of biomarkers that could distinguish patients with LNM. We aimed to verify the LNM-related prognostic biomarkers in LUAD.

**Methods:**

We firstly accessed the expression data of mRNA from The Cancer Genome Atlas (TCGA) database and then obtained samples with LNM (N+) and without LNM (N-). Differential expression analysis was conducted to acquire differentially expressed genes (DEGs). Univariate-LASSO-multivariate Cox regression analyses were performed on DEGs to build a risk model and obtain optimal genes. Afterwards, effectiveness and independence of risk model were assessed based on TCGA-LUAD and GSE31210 datasets. Moreover, a nomogram was established combining clinical factors and riskscores. Nomogram performance was measured by calibration curves. The infiltration abundance of immune cells was scored with CIBERSORT to explore the differences between high- and low-risk groups. Lastly, gene set enrichment analysis (GSEA) was used to investigate differences in immune features between the two risk groups.

**Results:**

Nine optimal feature genes closely related to LNM in LUAD were identified to construct a risk model. Prognostic ability of the risk model was verified in independent databases. Patients were classified into high- and low-risk groups in accordance with their median riskscores. CIBERSORT score displayed differences in immune cell infiltration like T cells CD4 memory resting between high/low-risk groups. LNM-related genes may also be closely relevant to immune features. Additionally, GSEA indicated that differential genes in the two risk groups were enriched in genes related to immune cells.

**Conclusion:**

This research built a risk model including nine optimal feature genes, which may be potential biomarkers for LUAD.

## 1. Introduction

Lung cancer is the leading cause of cancer death globally, with adenocarcinoma as the most prevalent histologic type [1]. Recent decades have witnessed great progress in cancer treatment; however, the prognosis of patients with lung adenocarcinoma (LUAD) still fails to be satisfying [2]. Studies demonstrated that lymph node metastasis (LNM) is pertinent to the survival rate of LUAD patients [3, 4]. As reported, the 5-year survival of LUAD patients with LNM is only 26-53%, while the survival rate of LUAD patients without LNM is more than 95% [5, 6]. Therefore, it is urgent to predict prognostic biomarkers that are related to LNM occurrence in LUAD and to identify patients with high mortality risk in advance.

The establishment and improvement of many databases further promote our understanding of disease genomic alterations, including identifying biomarkers related to tumor diagnosis and prognosis. For instance, Fu et al. [7] analyzed CEP55 expression in LUAD and lung squamous cell carcinoma (LUSC) by using The Cancer Genome Atlas (TCGA) and Gene Expression Omnibus (GEO) databases and validated by receiver operating characteristic (ROC) curves and univariate and multivariate Cox regression analyses. Ma et al. [8] screened survival related key genes by random survival forest based on TCGA-LUAD database, KM survival curve, and C-index method, and we validated the performance of these genes in GEO database, in which 13 genes as the prognostic biomarkers of LUAD were first reported. Zhang et al. [9] obtained sample cases based on TCGA database, and Cox regression analysis screened the signature genes and constructed the model, which finally yielded the correlation between a 9-gene signaling and glycolysis, and provided a new biomarker for LUAD patient's poor prognosis and metastasis. The above studies presented that abnormally expressed genes can act as prognostic or diagnostic biomarkers of LUAD. Nevertheless, rare was researched on LNM-related differentially expressed genes (DEGs) as prognostic biomarkers of LUAD.

Here, LUAD samples with or without LNM were obtained through TCGA database, which were subjected to differential expression analysis. Enrichment analysis was conducted on the acquired differential genes, and a risk model was built through regression analyses. Besides, immune infiltration abundance of LUAD samples was scored to unravel the correlation between immune infiltration and riskscores. The results may provide an effective prognostic tool for LUAD patients and assist doctors in identifying patients with high risk of mortality to increase their survival rate.

## 2. Materials and Methods

### 2.1. Data Source and Preprocessing

Firstly, mRNA data were accessed from TCGA (https://portal.gdc.cancer.gov/) database along with clinical data (Supplementary Table 1). Samples followed for more than 30 days were screened as the training set, including 330 non-LNM samples (N-) and 171 LNM samples (N+). LUAD gene expression data (tumor: 226) of GSE31210 (GPL570) were downloaded from GEO along with clinical information (Supplementary Table 2) as the validation set.

### 2.2. Differential Expression Analysis

This step was aimed at recognizing differentially expressed genes (DEGs) between LNM samples (N+) and non-LNM samples (N-). To that end, “edgeR” package [10] was applied to undertake differential expression analysis on N+ samples relative to N- samples. The screening threshold value was ∣logFC | >1 and FDR < 0.0510 [10].

### 2.3. Differential Gene Enrichment Analyses

For better understanding of biological processes that DEGs may participate, “clusterProfiler” package [11] was employed to perform Gene Ontology (GO) and Kyoto Encyclopedia of Genes and Genomes (KEGG) analyses. *p*adjust <0.05 and*q*value <0.05 were considered statistically significant [11].

### 2.4. Construction of the Risk Model

Univariate Cox regression analysis was performed on DEGs with “survival” package (*p* < 0.001) [12]. To avoid overfitting of the statistical model, LASSO regression analysis was conducted on genes screened by univariate analysis by using “glmnet” package [13]. Penalty parameter “lambda” was selected by cross validation method. Genes with strong correlation were removed to reduce model complexity (maxit = 5000) [14]. Multivariate Cox regression analysis was undertaken on LASSO regression analysis-screened genes by using “survival” package [15] to build a risk model and obtain optimal genes. Riskscore was calculated as follows:

(1) $$\begin{array}{c} \text{Riskscore}=\overset{n}{\underset{i=1}{\sum }}\left({\text{Coef}}_{i}\times x_{i}\right). \end{array}$$

In this formula, Coef_*i*_ is the cooperativity coefficient and *x*_*i*_ is the relative gene expression standardized by *Z*-score.

### 2.5. Model Assessment

We assessed the validity of the model in training set and validation set. Riskscores of samples were calculated according to the formula. Samples were divided into high- and low-risk groups by median score. Survival analysis was undertaken with survminer package (https://cran.r-project.org/web/packages/survminer/index.html). High- and low-risk groups were further divided by LNM occurrence to undertake survival analysis. Receiver operator characteristic (ROC) curve was drawn by using “timeROC” package [16] to calculate the area under the curve (AUC) values of 1-, 3-, and 5-year overall survival (OS) [16].

Riskscore was taken as a separate feature in the validation of the independence of the model. Univariate and multivariate Cox regression analyses were further conducted combining clinical data to evaluate the ability of the prognostic risk model to predict patient's survival status.

### 2.6. Construction and Evaluation of the Nomogram

A nomogram was generated by using “rms” package [17] combining clinical information and the risk model so as to predict the possibility of patient's 1-, 3-, and 5-year OS [17]. Calibration curves corresponded to 1-, 3-, and 5-year were plotted to validate the prediction efficacy of the nomogram.

### 2.7. Immune Analysis of High- and Low-Risk Groups

CIBERSORT was used to score the abundance of each immune cells in the training set. To increase the accuracy of deconvolution results, we only reserved data with CIBERSORT *p* value<0.05 and analyzed differences in the infiltration abundance of immune cells between high- and low-risk groups [18].

Immune function enrichment analysis was conducted in high- and low-risk groups by using the GSEA software. Gene sets used for analysis were c7: immunologic signature gene sets. Significant screening criterion was FDR < 0.25.

## 3. Results

### 3.1. Differential Expression Analysis

**Figure 1:**
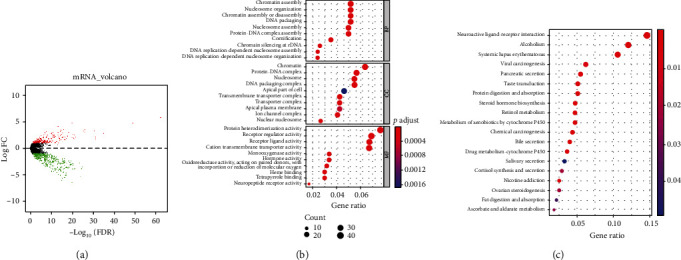
Acquisition and analysis of DEGs. (a) Volcano plot of differential analysis on N+ relative to N- group. Red refers to upregulated genes, and green refers to downregulated genes. (b) GO enrichment analysis. (c) KEGG enrichment analysis.

Differential analysis was conducted on N+ samples relative to N- samples, and 637 DEGs were obtained including 196 upregulated genes and 441 downregulated ones (Figure 1(a)). Enrichment analyses were undertaken on these 637 DEGs. GO analysis suggested that these genes were mainly enriched in biological functions like chromatin assembly, DNA packaging, protein-DNA complex assembly, and ligand (Figure 1(b)). The result of KEGG manifested that these genes were mainly enriched in biological pathways like retinol metabolism, metabolism of xenobiotics by cytochrome P450, and viral carcinogenesis (Figure 1(c)). The above results indicated that these DEGs may participate in chromatin assembly, DNA packaging, retinol metabolism, and so on.

### 3.2. Construction of the Risk Model

**Figure 2:**
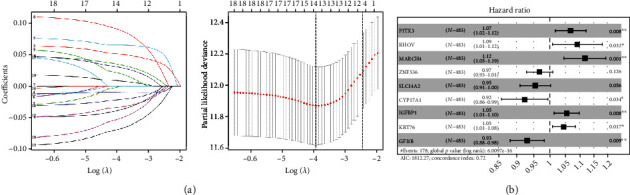
Establishment of the risk model. (a) LASSO regression analysis was undertaken on 19 genes obtained. Fourteen feature genes were finally screened. (b) Forest plot of 9 optimal feature genes which obtained from multivariate Cox regression analysis. ^∗^*p* < 0.05. ^∗∗^*p* < 0.01.

Univariate Cox regression analysis was performed on the above 637 DEGs and obtained 19 prognosis-related genes (Supplementary Table 3). To avoid overfitting of the model, LASSO regression analysis of these 19 genes screened 14 feature genes (Figure 2(a)), which were then subjected to multivariate Cox recession analysis, and 9 optimal feature genes were finally screened (Figure 2(b)). Riskscore = 0.066∗PITX3 + 0.087∗RHOV + 0.111∗MARCH4–0.033∗ZNF536–0.047∗SLC14A2–0.079∗CYP17A1 + 0.053∗IGFBP1 + 0.044∗KRT76–0.072∗GFI1B. Hence, we obtained 9 optimal feature genes through regression analyses to assess LUAD patient's prognostic risk.

### 3.3. Assessment of the Risk Model

**Figure 3:**
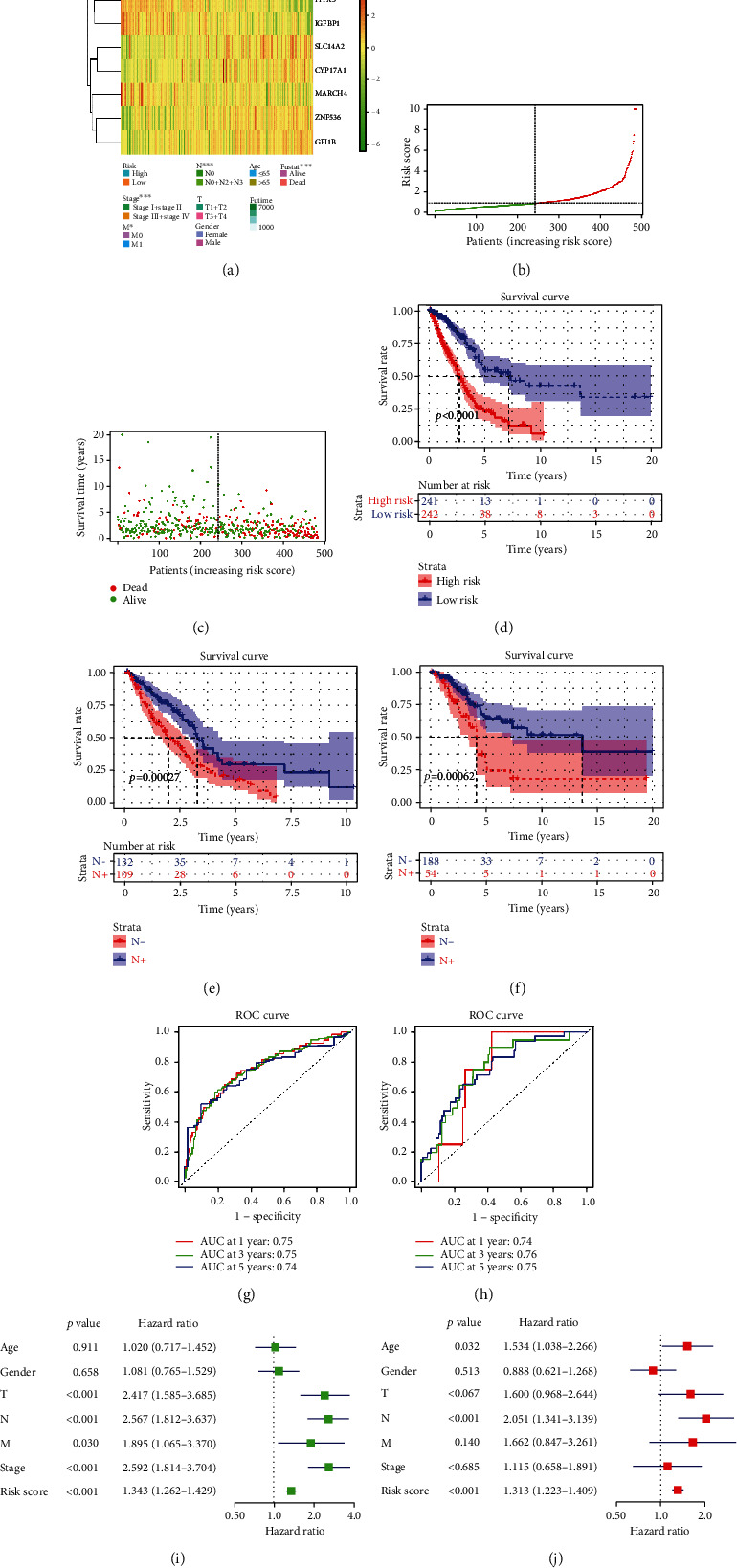
Evaluation of the risk model. (a) Heat map of the expression of 9 genes. (b) Distribution plot of riskscore in each group. (c) Distribution plot of survival status in each group. (d) Survival curve graph of patients (red: high-risk group and blue: low-risk group). (e) Survival curve graph of in N+ and N- patients in the high-risk group (red: N+ patients and blue: N- patients). (f) Survival curve graph of patients in N+ group and N- group in the low-risk group (red: N+ patients and blue: N- patients). (g) ROC curve graph of the risk model in TCGA dataset. (h) ROC curve of the risk model in GSE31210 dataset. (i) Forest plot of univariate and (j) multivariate Cox regression analyses.

Afterwards, we assessed validity and independence of the model. According to heat map of the levels of these 9 optimal feature genes in high- and low-risk groups (Figure 3(a)) combined with clinical features, significant differences were exhibited in stages M and N and survival status between the two risk groups. Patient's survival rate in high-risk group was prominently lower than that in the low-risk group through distribution of the riskscore of 9 optimal feature genes (Figure 3(b)), patient's survival status (Figure 3(c)), and survival curves (Figure 3(d)) in two risk groups. Moreover, the survival rate of N+ patients was lower relative to N- patients in two risk groups (Figures 3(e) and 3(f)). Besides, the performance of the risk model on determining patient's prognosis was evaluated by ROC curve. The 1-, 3-, and 5-year AUC values in training set were, respectively, 0.75, 0.75, and 0.74 (Figure 3(g)), while those in validation set were 0.74, 0.76, and 0.75, respectively (Figure 3(h)). This testified that the risk model based on the training set possessed good determining function on LUAD patient's prognosis. In addition, expression of these 9 optimal feature genes was closely associated with LNM occurrence. Univariate and multivariate Cox regression analyses were conducted on riskscores and clinical data to assess the independence of the model. It was found that riskscores were statistically significant to patient's prognosis (Figures 3(i) and 3(j)). To conclude, the 9-gene risk model suggested a good prognostic effect, and riskscore could be used as an independent factor for the prognosis of LUAD patients.

### 3.4. Construction and Evaluation of the Nomogram

**Figure 4:**
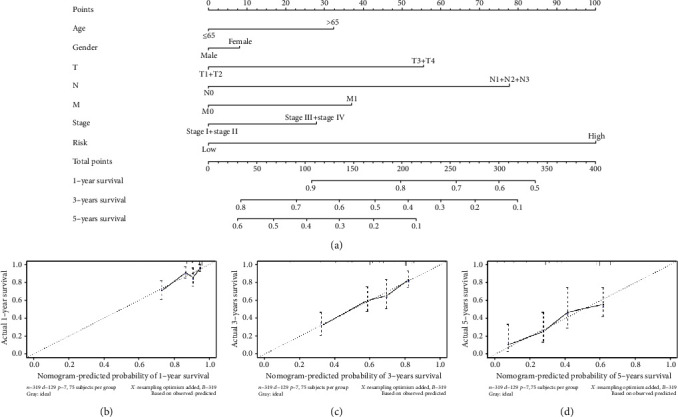
Nomogram establishment and evaluation. (a) Nomogram based on the prognostic riskscore of the 9 feature genes combining clinical data. (b)–(d) Calibration curves of 1-, 3-, and 5-year.

To better apply our model to clinical practice, we established a nomogram which could better predict LUAD patient's prognosis. The nomogram was drawn with risk type (low/high) along with patient's clinical data (age, sex, TNM stage, and clinical stages) to predict the possibility of patient's 1-, 3-, and 5-year survival (Figure 4(a)). Its performance was visualized by calibration curve (45° line referred to the optimal performance). The high fitting of 1-, 3-, and 5-year calibration curves demonstrated a good performance (Figures 4(b)–4(d)). The above results suggested that the nomogram may assist doctors to decide the plan for following treatment of LUAD patients.

### 3.5. Riskscore Was Related to Tumor Immune Infiltration

**Figure 5:**
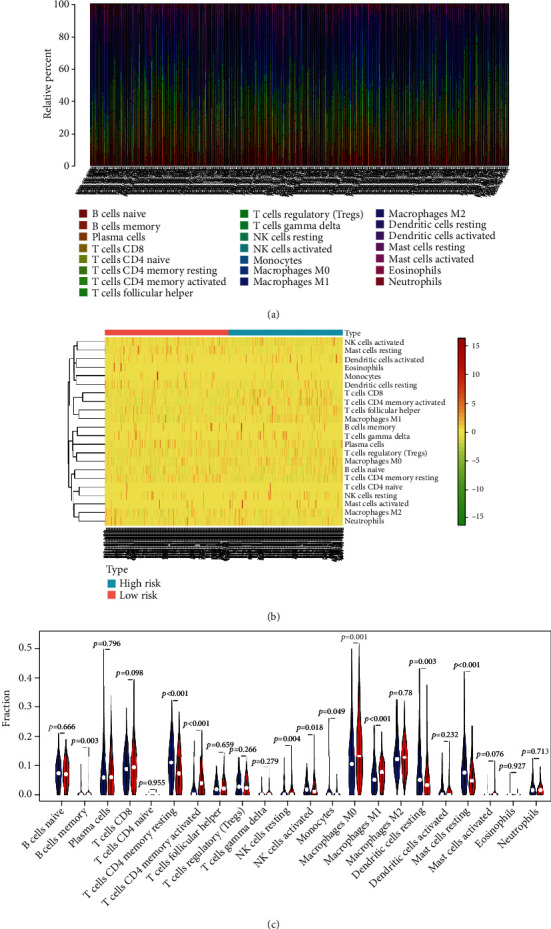
Riskscore was related to tumor immune infiltration. (a) Histogram of infiltration abundance ratio of each immune cells. (b) Heat map of infiltration abundance. (c) Violin plot of differences in each immune cell infiltration abundance (red: high-risk group and blue: low-risk group).

The infiltration abundance of each immune cells in samples was scored by using CIBERSORT algorithm. A total of 184 low-risk samples and 170 high-risk samples were obtained after screening the samples with *p* value<0.05 (Supplementary Table 4). Histogram and heat map based on CIBERSORT exhibited the degree of immunity in the high- and low-risk groups (Figures 5(a) and 5(b)). Infiltration abundance of T cells CD4 memory resting, NK cells activated, dendritic cells resting, and mast cells resting in the high-risk group was prominently downregulated relative to the low-risk group, while infiltration abundance of T cells CD4 memory activated, macrophages M0, and macrophages M1 was significantly upregulated (Figure 5(c)). The above results showed remarkable differences in immune cell infiltration between two risk groups.

### 3.6. GSEA Enrichment Analysis in High- and Low-Risk Groups

**Figure 6:**
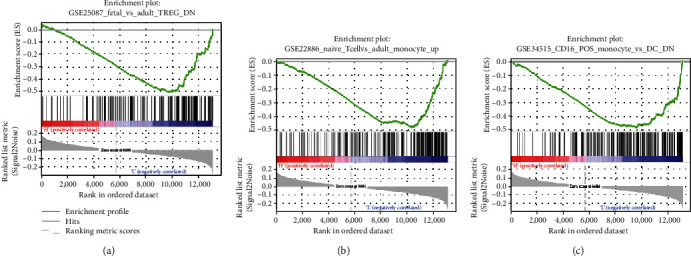
GSEA analysis of feature genes. Differential gene enrichment in high- and low-risk groups in (a) FETAL_VS_AUDULT_TREG_DN, (b) NAIVE_TCELL_VS_MONOCYTE_UP, and (c) CD16_POS_MONOCYTE_VS_DC_DN.

Lastly, to explore the differences in immune features between the groups, GSEA was performed in these two groups. As the results suggested, differential genes in the high- and low-risk groups were enriched in FETAL_VS_AUDULT_TREG_DN, NAIVE_TCELL_VS_MONOCYTE_UP, and CD16_POS_MONOCYTE_VS_DC_DN (Figures 6(a)–6(c)). It was showed that the two groups manifested statistical significances in immune features of immune cells including regulatory T cells and dendritic cells. These pathways may trigger significant differences in prognosis.

## 4. Discussion

Accumulating evidence demonstrated that LUAD patients with LNM often have a poor response to standard treatment and shorter survival time [19]. It is urgent to classify these patients in advance and predict their prognosis to help clinicians to better make targeted treatment plans. Nevertheless, a single biomarker cannot accurately or independently evaluate patient's prognosis and is often affected by other clinical factors [19]. Furthermore, clinical stages established by patient- and tumor-related factors are limited in accuracy and specificity, such as AJCC-TNM stage [20]. A study found that multiple-gene signature is a better choice for predicting patient's prognosis and survival [21]. Hence, our study was aimed at identifying molecular biomarkers related to LNM in LUAD for better prediction of patient's prognosis.

In the present study, DEGs were obtained via analyzing gene expression profiles of N+ and N- samples in TCGA-LUAD dataset. Enrichment analyses suggested that the expression of the DEGs was associated with tumor development. To further screen genes relevant to patient's prognosis, regression analyses were undertaken. Finally, 9 optimal feature genes were acquired, and a risk model was constructed. Riskscore = 0.066∗PITX3 + 0.087∗RHOV + 0.111∗MARCH4–0.033∗ZNF536–0.047∗SLC14A2–0.079∗CYP17A1 + 0.053∗IGFBP1 + 0.044∗KRT76–0.072∗GFI1B. Previous studies demonstrated that the above genes are relevant to patient's survival and prognosis. For instance, elevated RHOV expression level correlates with NSCLC patient's poor survival [22]. High KRT76 expression is associated with increased tumor susceptibility [23]. Subsequently, we evaluated the constructed model and found that the high-risk group showed short survival. N+ patients had shorter survival relative to N- patients. Moreover, ROC curve showed the favorable performance of the model. To help clinicians to predict patients with high mortality risk, we built a nomogram with riskscores and clinical factors. The calibration curve suggested the good performance of the nomogram.

A clinical study illustrated that immune activation in tumor cells is closely relevant to LNM [24]. To this end, we also scored infiltration abundance. It was exhibited that relative to the low-risk group, the infiltration abundance of NK cells activated, T cells CD4 memory resting, dendritic cells resting, and mast cells resting was remarkably downregulated, while the infiltration abundance of macrophages M0, T cells CD4 memory activated, and macrophages M1 was prominently upregulated. Melaiu et al. [25] presented that high density of tumor infiltrating NK cells is associated with the excellent prognosis of various solid tumors. Mast cells, known as mastocytes, are key regulators of immune effector cells [26–28]. The infiltration of mast cells positively pertains to the prognosis of gastric cancer [26–28]. Padoan *et al*. [29] indicated that memory CD4+ T cells generate interleukin to accelerate tumorigenesis, which explains potential factors for upregulation of activated CD4+ T cells in the high-risk group. Macrophages were considered as a major cell type to connect inflammation and cancer, among which M1 can activate inflammation to stimulate cancer progression [30]. In addition, Xiao et al. [31] confirmed that M1 macrophages initiated by exosome-delivered THBS1 exacerbate malignant progression of oral squamous cell carcinoma. Results of the above references were similar to the results here, further demonstrating that optimal feature genes related to LNM were closely associated with immune infiltration degree. GSEA enrichment analysis was conducted to better understand the difference between the two groups. They displayed statistical differences in immune degree of immune cells like regulatory T cells and dendritic cells. A reference elaborated that Treg cells abundantly infiltrate tumor tissue, relating cancer patient's poor prognosis [32]. Haak *et al*. [33] found that the OS of tumor patients with high infiltration of CD16+ cells is evidently longer. Dendritic cells are the main modulators of adaptive immune response and indispensable for T cell-manipulated cancer immunity [34]. This explains that differences in immune features may be one of the reasons for poor prognosis.

In sum, according to TCGA-LUAD data, we constructed an effective 9-gene risk prognostic model that could predict patient's prognosis independent of other clinical factors. Our model was able to divide LUAD patients into two groups and effectively distinguish patients with poor prognosis. Moreover, the identified feature genes may play a predictive role to a certain extent in immune treatment. However, limitations still exist here. In the future, we plan to analyze the expression of optimal feature genes and immune checkpoints to further validate underlying value of the optimal feature genes in predicting the efficacy of treatment with immune checkpoint inhibitors.

## Data Availability

The data used to support the findings of this study are available from the corresponding author upon request.

## Supplementary Materials

Supplementary 1
*Supplementary Table 1*. Clinical data of TCGA-LUAD samples.

Supplementary 2
*Supplementary Table 2*. Clinical data of LUAD samples in GSE31210 dataset.

Supplementary 3
*Supplementary Table 3*. The 19 prognosis-related genes obtained.

Supplementary 4
*Supplementary Table 4*. Screened samples after CIBERSORT score.
