# Supplementary material for: Construction of Lymph Node Metastasis-Related Prognostic Model and Analysis of Immune Infiltration Mode in Lung Adenocarcinoma
Source: Comput Math Methods Med. 2022 Jun 29;2022:3887857. doi: 10.1155/2022/3887857 (PMC9274234; doi:10.1155/2022/3887857)
Supplement: Supplementary 1 — Supplementary Table 1. Clinical data of TCGA-LUAD samples. [file 3887857.f1.pdf]

id	fustat	futime	age	gender	T	N	M	tumor_stage	smoking	tumor_grade
TCGA-55-A48Z	Alive	651	22056	female	T1a	N3	MX	stage iiib	40	not reported
TCGA-86-A4JF	Dead	737	20491	male	T3	N0	M0	stage iib	None	not reported
TCGA-55-6986	Alive	3261	None	female	T2	N0	M0	stage ib	None	not reported
TCGA-NJ-A4YP	Alive	50	19106	male	T2a	N0	M0	stage ib	None	not reported
TCGA-78-7148	Dead	626	25990	male	T2	N1	M0	stage iib	None	not reported
TCGA-97-8176	Dead	468	23170	male	T3	N1	M0	stage iiia	40	not reported
TCGA-86-8674	Alive	806	18479	male	T2a	N1	M0	stage iia	30	not reported
TCGA-L9-A8F4	Alive	476	23617	female	T2a	N0	MX	stage ib	None	not reported
TCGA-55-7576	Alive	670	19917	male	T2a	N0	M0	stage ib	None	not reported
TCGA-55-8507	Alive	418	19390	male	T1a	N0	MX	stage ia	None	not reported
TCGA-97-A4LX	Alive	614	29915	male	T2a	N0	M0	stage ib	40	not reported
TCGA-50-8457	Alive	1125	23055	female	T1a	N0	M0	stage ia	None	not reported
TCGA-80-5611	Alive	2595	None	male	T2	N0	M0	stage ib	None	not reported
TCGA-78-7145	Dead	826	19080	female	T4	N1	M1	stage iv	25	not reported
TCGA-62-A46P	Dead	594	23993	male	T2	N0	M0	stage ib	None	not reported
TCGA-64-5779	Alive	864	22305	male	T2	N2	M0	stage iiia	40	not reported
TCGA-55-7903	Alive	567	23509	male	T1b	N0	MX	stage ia	None	not reported
TCGA-50-5051	Dead	478	15585	female	T2	N2	M0	stage iiia	None	not reported
TCGA-55-8087	Alive	462	21655	female	T2a	N0	MX	stage ib	None	not reported
TCGA-55-7570	Alive	824	22218	male	T1a	N0	MX	stage ia	None	not reported
TCGA-86-A4P8	Alive	805	21738	female	T1b	N2	MX	stage iiia	None	not reported
TCGA-05-4389	Alive	1369	25660	male	T1	N0	M0	stage ia	None	not reported
TCGA-53-7813	Alive	424	18875	female	T4	N0	M0	stage iiib	35	not reported
TCGA-69-7978	Alive	134	21785	male	T2b	N1	MX	stage iib	None	not reported
TCGA-75-6207	Dead	134	None	male	T2	N2	M0	stage iiia	None	not reported
TCGA-75-6211	Dead	134	None	female	T2	N0	M0	stage ib	None	not reported
TCGA-62-A470	Dead	1194	30689	male	T2	N0	M0	stage ib	None	not reported
TCGA-NJ-A7XG	Alive	617	18127	male	T4	N1	M0	stage iiia	None	not reported
TCGA-05-5428	Alive	670	21061	male	T1b	N1	M0	stage iia	24	not reported
TCGA-MP-A4TK	Dead	582	20581	female	T2	N1	MX	stage iib	28	not reported
TCGA-L9-A443	Dead	193	23062	female	T1a	N0	MX	stage ia	45	not reported
TCGA-64-1680	Alive	1126	23267	male	T2a	N2	M1	stage iv	20	not reported
TCGA-69-7974	Alive	184	20021	female	T2a	N2	MX	stage iiia	30	not reported
TCGA-05-4415	Dead	91	20880	male	T4	N2	M0	stage iiib	10	not reported
TCGA-78-7156	Dead	976	22720	male	T4	N1	M1	stage iv	28	not reported
TCGA-55-8204	Alive	515	31818	female	T2a	N0	MX	stage ib	None	not reported
TCGA-05-4426	Alive	791	26084	male	T2	N0	M0	stage ib	None	not reported

TCGA-49-4490	Dead	385	16634	female	T3	N2	M0	stage iiia	None	not reported
TCGA-50-5935	Dead	653	31610	female	T1	N0	M0	stage ia	None	not reported
TCGA-50-6597	Dead	1268	29195	female	T2	N0	M0	stage ib	None	not reported
TCGA-75-5146	Alive	2368	None	male	T2	N0	M0	stage ib	None	not reported
TCGA-44-A47A	Alive	466	28782	female	T2a	N0	MX	stage ib	40	not reported
TCGA-62-A46S	Dead	1653	26825	male	T2	N0	M0	stage ib	None	not reported
TCGA-38-4625	Alive	2973	24241	female	T2a	N0	M0	stage ib	None	not reported
TCGA-91-A4BD	Alive	603	28544	male	T1b	N1	MX	stage iia	None	not reported
TCGA-67-3772	Alive	573	29989	female	T2	N0	M0	stage ib	None	not reported
TCGA-49-AAR4	Dead	879	18939	male	T2	N2	MX	stage iiia	None	not reported
TCGA-49-4486	Dead	2318	26415	male	T1	N0	M0	stage ia	35	not reported
TCGA-55-7995	Alive	889	26837	female	T1b	N0	M0	stage ia	None	not reported
TCGA-69-7979	Alive	408	26180	female	T2a	N0	MX	stage ib	None	not reported
TCGA-50-8459	Alive	1119	24859	male	T3	N0	M0	stage iib	None	not reported
TCGA-35-3615	Alive	14	21076	male	T2	N0	M0	stage ib	None	not reported
TCGA-55-7914	Dead	187	26105	female	T1b	N1	MX	stage iia	None	not reported
TCGA-50-5936	Dead	257	21238	male	T2	N2	M0	stage iiia	None	not reported
TCGA-78-7535	Dead	949	16571	male	T2	N0	M0	stage ib	None	not reported
TCGA-97-A4M7	Alive	629	27062	male	T1b	N0	M0	stage ia	20	not reported
TCGA-55-1596	Alive	2065	20381	male	T2	N1	M0	stage iib	None	not reported
TCGA-50-5931	Dead	434	27733	female	T2	N0	M0	stage ib	None	not reported
TCGA-97-A4M1	Alive	601	19272	female	T1a	N0	M0	stage ia	3	not reported
TCGA-NJ-A55O	Alive	13	20628	female	T1b	N1	M0	stage iia	None	not reported
TCGA-05-4425	Alive	669	25902	female	T2	N0	M1	stage iv	None	not reported
TCGA-93-A4JQ	Alive	526	17951	male	T1b	N0	MX	stage ia	20	not reported
TCGA-44-A47B	Alive	287	28869	male	T2a	N0	M0	stage ib	9	not reported
TCGA-50-5941	Alive	1474	20350	female	T2a	N2	M0	stage iiia	None	not reported
TCGA-73-4666	Alive	800	19074	female	T1	N0	M1	stage iv	20	not reported
TCGA-44-2664	Alive	1251	24468	female	T1	N0	M0	stage ia	None	not reported
TCGA-44-3396	Alive	1130	27073	female	T2	N2	M0	stage iiia	None	not reported
TCGA-97-8174	Dead	164	24725	male	T2b	N0	M0	stage iia	20	not reported
TCGA-50-5045	Dead	2174	20961	female	T2	N1	M0	not reported	None	not reported
TCGA-73-4658	Dead	1600	29508	female	T2	N0	M0	stage ib	None	not reported
TCGA-44-5644	Alive	863	18644	female	T2a	N0	none	stage ib	None	not reported
TCGA-75-5126	Alive	None	None	female	T3	N2	M0	stage iiia	None	not reported
TCGA-62-A472	Alive	910	25893	male	T3	N0	M0	stage iib	None	not reported
TCGA-J2-A4AG	Alive	988	24224	female	T1b	N0	MX	stage ia	25	not reported
TCGA-38-7271	Dead	800	26440	female	T1	N0	M0	stage ia	28	not reported

TCGA-78-8648	Dead	1209	21443	female	T3	N0	M0	stage iib	None	not reported
TCGA-44-3919	Dead	1026	26081	female	T1	N0	M0	stage ia	None	not reported
TCGA-MP-A4TF	Dead	336	21387	female	T2b	N0	M0	stage iia	40	not reported
TCGA-55-8094	Alive	541	18903	male	T2b	N0	M1b	stage iv	35	not reported
TCGA-44-6146	Alive	728	23378	male	T3	N0	M0	stage iib	None	not reported
TCGA-NJ-A55A	Alive	15	28022	female	T2	N0	M0	stage ib	None	not reported
TCGA-73-4675	Dead	922	21614	male	T3	N1	M0	stage iiia	41	not reported
TCGA-75-7030	Alive	None	None	male	T3	N0	M0	stage iib	None	not reported
TCGA-55-8096	Dead	719	24684	female	T2a	N0	MX	stage ib	None	not reported
TCGA-44-8119	Alive	285	27003	male	T3	N0	M0	stage iib	None	not reported
TCGA-97-A4M0	Alive	652	22206	female	T2a	N0	M0	stage ib	34	not reported
TCGA-86-8669	Alive	938	23443	male	T1b	N0	M0	stage ia	None	not reported
TCGA-05-5715	Alive	62	25383	female	T2a	N0	M0	stage ib	None	not reported
TCGA-55-8203	Alive	547	25477	female	T1b	N0	M0	stage ia	None	not reported
TCGA-50-6590	Dead	1288	26444	female	T2	N0	M0	stage ib	None	not reported
TCGA-93-A4JP	Alive	578	23620	male	TX	NX	M1b	stage iv	None	not reported
TCGA-97-A4M6	Alive	568	16764	female	T1a	N0	M0	stage ia	None	not reported
TCGA-55-8510	Alive	539	20410	female	T2a	N0	MX	stage ib	None	not reported
TCGA-73-7499	Dead	1531	29683	female	T2a	N0	M0	stage ib	None	not reported
TCGA-55-7574	Dead	995	23703	female	T2a	N0	M0	stage ib	40	not reported
TCGA-05-4403	Alive	578	27881	male	T2	N0	M0	stage ib	None	not reported
TCGA-55-6968	Dead	1293	22344	male	T1	N0	M1	stage iv	None	not reported
TCGA-49-4506	Dead	999	25132	female	T2	N1	M0	stage iib	None	not reported
TCGA-44-6777	Dead	987	31234	female	T2	NX	MX	stage ib	64	not reported
TCGA-05-4405	Alive	610	27241	female	T2	N0	M0	stage ib	43	not reported
TCGA-55-8207	Alive	977	26897	male	T2a	N0	MX	stage ib	None	not reported
TCGA-50-8460	Alive	829	27270	male	T1a	N0	M0	stage ia	None	not reported
TCGA-75-5125	Dead	2027	None	male	T2	N1	M0	stage iib	None	not reported
TCGA-05-5425	Alive	882	25020	male	T2b	N1	M0	stage iib	20	not reported
TCGA-J2-A4AE	Alive	1079	28163	female	T1a	N0	MX	stage ia	None	not reported
TCGA-78-8660	Dead	321	25422	male	T2	N1	M0	stage iib	51	not reported
TCGA-55-6642	Alive	2449	23107	male	T2	N0	MX	stage ib	None	not reported
TCGA-86-6851	Alive	179	26808	female	T1b	N1	M0	stage iia	None	not reported
TCGA-05-4432	Alive	761	24350	male	T2	N1	M0	stage iib	None	not reported
TCGA-62-8397	Alive	1289	25728	female	T3	N0	M0	stage iib	None	not reported
TCGA-64-5778	Alive	1305	21927	male	T2	N0	M0	stage ib	34	not reported
TCGA-78-7152	Dead	1215	23782	male	T2	N0	M0	stage ib	40	not reported
TCGA-49-AAR3	Alive	1893	25469	male	T2	N1	MX	stage iib	None	not reported

TCGA-49-AAQV	Dead	677	23370	female	T1	N1	MX	stage ii	None	not reported
TCGA-J2-8194	Alive	724	25441	female	T3	N0	MX	stage iib	26	not reported
TCGA-49-AAR9	Dead	260	22510	male	T3	N0	MX	stage iib	None	not reported
TCGA-55-8619	Alive	416	26616	female	T3	N0	MX	stage iib	None	not reported
TCGA-55-7913	Dead	561	22326	female	T1b	N0	MX	stage ia	None	not reported
TCGA-99-8028	Alive	1118	18542	female	T1a	N0	M0	stage ia	30	not reported
TCGA-55-6712	Dead	171	None	male	T2a	N1	MX	stage iia	33	not reported
TCGA-78-7162	Dead	3169	27593	male	T1	N0	M0	stage ia	None	not reported
TCGA-MP-A4T4	Dead	2617	25052	female	T2	N1	M0	stage iib	None	not reported
TCGA-50-5066	Alive	1442	26385	male	T2	N0	M0	stage ib	None	not reported
TCGA-50-6592	Dead	777	26191	female	T2	N0	M0	stage ib	None	not reported
TCGA-62-A46V	Alive	2199	28495	female	T2	N0	M0	stage ib	None	not reported
TCGA-83-5908	Alive	824	21760	female	T1	N0	M0	stage ia	45	not reported
TCGA-44-7672	Alive	719	19200	female	T1b	N0	M0	stage ia	None	not reported
TCGA-O1-A52J	Dead	1798	27223	female	T1	N0	MX	stage ia	30	not reported
TCGA-86-7953	Alive	997	25315	female	T1b	N0	M0	stage ia	None	not reported
TCGA-62-8394	Dead	139	23758	female	T4	N2	M0	stage iiib	None	not reported
TCGA-97-8172	Alive	545	27416	female	T2a	N0	M0	stage ib	20	not reported
TCGA-55-6987	Alive	2137	28382	male	T1	N0	M0	stage ia	None	not reported
TCGA-MP-A4T7	Dead	167	27603	female	T2	N0	M1	stage iv	50	not reported
TCGA-49-4488	Dead	869	27332	female	T1	N0	MX	stage ia	None	not reported
TCGA-73-4659	Dead	711	24253	male	T2	N2	M0	stage iiia	12	not reported
TCGA-78-7160	Dead	697	22385	male	T4	N2	M1	stage iv	35	not reported
TCGA-49-4507	Dead	268	26851	female	T3	N1	M0	stage iiia	None	not reported
TCGA-35-5375	Alive	264	22628	male	T2	N2	M0	stage iiia	35	not reported
TCGA-MP-A4T8	Dead	161	25069	male	T2	N2	M0	stage iiia	15	not reported
TCGA-62-8402	Dead	1498	26934	female	T2	N2	M0	stage iiia	None	not reported
TCGA-75-5147	Alive	1333	None	female	T2	N0	M0	stage ib	None	not reported
TCGA-55-6979	Dead	237	21780	female	T2	N1	M0	stage iib	None	not reported
TCGA-NJ-A55R	Alive	603	24738	male	T1b	N0	MX	stage ia	None	not reported
TCGA-55-8205	Alive	599	28063	female	T2b	N0	M0	stage iia	61	not reported
TCGA-49-6742	Dead	488	25839	male	T2a	N1	M0	stage iia	None	not reported
TCGA-49-AARR	Alive	4992	24942	male	T1	N0	MX	stage ia	None	not reported
TCGA-67-3770	Alive	610	25658	female	T1	N0	M0	stage ia	None	not reported
TCGA-MP-A4TD	Dead	307	25983	male	T2	N2	M0	stage iiia	None	not reported
TCGA-55-6970	Dead	464	24773	female	T2	N2	MX	stage iiia	None	not reported
TCGA-73-4670	Alive	131	25526	female	T2	N0	M1	stage iv	30	not reported
TCGA-97-8171	Alive	568	29627	male	T2a	N2	M1a	stage iv	None	not reported

TCGA-55-6969	Alive	1239	19270	male	T2	N0	M0	stage ib	None		not reported
TCGA-69-A59K	Alive	591	22093	female	T3	N0	M0	stage iib		13	not reported
TCGA-55-6980	Alive	2109	None	male	T1	N0	M0	stage ia	None		not reported
TCGA-55-6978	Dead	176	None	male	T2b	N0	MX	stage iia	None		not reported
TCGA-53-7624	Dead	1043	14794	female	T2	N0	M1	stage iv	None		not reported
TCGA-44-6776	Alive	2616	22150	female	T1	N0	MX	stage ia		45	not reported
TCGA-93-7347	Alive	683	27949	female	T1a	N0	MX	stage ia		15	not reported
TCGA-86-8280	Alive	701	19977	female	T2b	N0	M0	stage iia	None		not reported
TCGA-38-4632	Dead	1357	15418	male	T2	N1	M1	stage iv	None		not reported
TCGA-55-8301	Alive	534	21535	male	T2a	N0	MX	stage ib	None		not reported
TCGA-49-4487	Dead	855	26583	female	T1	N0	M0	stage ia	None		not reported
TCGA-55-7284	Dead	243	27087	male	T3	N0	MX	stage iib	None		not reported
TCGA-49-4510	Dead	896	18762	female	T2	N1	M0	stage iib	None		not reported
TCGA-05-4402	Dead	244	20819	female	T2	NX	M1	stage iv	None		not reported
TCGA-78-7149	Alive	3940	26285	male	T4	N0	M0	stage iiib	None		not reported
TCGA-55-8206	Alive	888	20690	male	T1b	N0	M0	stage ia	None		not reported
TCGA-86-8073	Alive	740	21214	male	T2a	N0	M0	stage ib	None		not reported
TCGA-55-A48Y	Alive	630	25384	male	T2b	N0	M0	stage iia	None		not reported
TCGA-69-7765	Alive	165	20625	male	T4	N0	MX	not reported		30	not reported
TCGA-62-8395	Alive	1216	29311	female	T3	N0	M0	stage iib	None		not reported
TCGA-05-4420	Alive	912	15159	male	T2	N0	M0	stage ib	None		not reported
TCGA-67-3774	Alive	385	26704	female	T2	N0	M0	stage ib	None		not reported
TCGA-05-4249	Alive	1523	24532	male	T2	N0	M0	stage ib	None		not reported
TCGA-MP-A4TA	Dead	950	27513	female	T1	N0	M0	stage ia		55	not reported
TCGA-78-7220	Dead	807	19480	female	T2	N2	M0	stage iiia	None		not reported
TCGA-44-2668	Dead	761	18856	male	T2	N0	M0	stage ib		30	not reported
TCGA-91-6849	Alive	35	27676	female	T2	N2	MX	stage iiia		30	not reported
TCGA-97-7552	Alive	1932	25578	male	T2	N0	MX	stage ib	None		not reported
TCGA-44-8117	Alive	385	19855	female	T2a	N0	M0	stage ib		36	not reported
TCGA-67-3776	Alive	61	20940	female	T2	N1	M0	stage iib	None		not reported
TCGA-55-7910	Alive	1040	18289	female	T2b	N0	M0	stage iia		15	not reported
TCGA-38-4628	Dead	1492	24057	female	T2	N1	M0	stage iib	None		not reported
TCGA-44-5645	Alive	852	22471	female	T1	NX	none	stage ia		10	not reported
TCGA-78-7540	Dead	1197	24440	female	T2	N0	M0	stage ib	None		not reported
TCGA-78-7536	Dead	244	25558	male	T2	N2	M0	stage iiia		44	not reported
TCGA-67-6215	Alive	174	19310	female	T2a	N0	M0	stage ib	None		not reported
TCGA-55-8089	Dead	702	20576	male	T1a	N0	M0	stage ia	None		not reported
TCGA-97-8552	Alive	626	20331	female	T1a	N0	MX	stage i	None		not reported

TCGA-44-A47G	Alive	351	26800	female	T1	N0	M0	stage ia	56	not reported
TCGA-53-A4EZ	Alive	1071	23201	male	T2a	N1	MX	stage iia	40	not reported
TCGA-97-7547	Alive	1965	24737	female	T2	N0	MX	stage ib	None	not reported
TCGA-73-A9RS	Dead	340	15162	male	T3	N0	M0	stage iib	None	not reported
TCGA-05-4434	Dead	457	24472	female	T4	N1	M1	stage iv	None	not reported
TCGA-55-6543	Alive	435	None	female	T1b	N0	MX	stage ia	None	not reported
TCGA-MP-A4SV	Dead	2620	24516	male	T2	N0	M0	stage ib	None	not reported
TCGA-55-8614	Alive	536	27924	male	T2a	N0	MX	stage ib	None	not reported
TCGA-80-5608	Alive	2832	None	female	T1	N0	M0	stage ia	None	not reported
TCGA-95-7944	Alive	377	26003	male	T1a	N0	M0	stage ia	None	not reported
TCGA-49-4494	Dead	1081	28387	male	T3	N2	M0	stage iiia	25	not reported
TCGA-78-8655	Alive	2360	28379	female	T1	N0	M0	stage ia	20	not reported
TCGA-55-8097	Alive	476	22260	female	T1a	N0	MX	stage ia	35	not reported
TCGA-55-8616	Alive	48	21225	female	T2a	N0	M0	stage ib	None	not reported
TCGA-44-3918	Alive	1036	22236	female	T1	N0	M0	stage ia	20	not reported
TCGA-50-5930	Dead	282	17238	male	T2	N2	M0	stage iiia	None	not reported
TCGA-91-6829	Dead	1258	28841	male	T2	N0	MX	stage ib	63	not reported
TCGA-55-8091	Alive	600	27385	male	T2	N0	MX	stage ib	None	not reported
TCGA-05-4390	Alive	1126	21430	female	T2	N0	M0	stage ib	30	not reported
TCGA-99-8025	Alive	1060	26634	female	T3	N2	M0	stage iiia	40	not reported
TCGA-05-4410	Alive	0	22888	male	T2	N0	M0	stage ib	49	not reported
TCGA-44-5643	Alive	1013	19426	male	T2b	N2	M0	stage iiia	None	not reported
TCGA-44-2662	Alive	1280	24014	male	T2	N0	M0	stage ib	28	not reported
TCGA-05-4397	Dead	731	23833	male	T2	N1	M0	stage iib	None	not reported
TCGA-38-4627	Dead	1147	23510	female	T1b	N1	M0	stage iia	None	not reported
TCGA-99-AA5R	Alive	658	25778	female	T1a	N0	M0	stage ia	None	not reported
TCGA-50-5944	Alive	1750	25440	female	T1	N0	M0	stage ia	None	not reported
TCGA-64-5775	Dead	62	26058	male	T4	N0	M0	stage iiia	None	not reported
TCGA-86-8673	Alive	862	22571	male	T2	N0	M0	stage ib	None	not reported
TCGA-78-7146	Dead	173	26272	female	T2	N2	M0	stage iiia	10	not reported
TCGA-50-5068	Dead	1499	21728	female	T2	N1	MX	stage iib	None	not reported
TCGA-97-7546	Alive	1285	27913	female	T1	N0	MX	stage ia	20	not reported
TCGA-78-7158	Dead	179	21742	female	T4	N2	M0	stage iiib	42	not reported
TCGA-50-6595	Dead	189	27197	female	T2	N2	M0	stage iiia	None	not reported
TCGA-MP-A4T6	Dead	1790	27993	female	T1	N2	MX	stage iiia	None	not reported
TCGA-55-7907	Dead	343	28201	male	T2a	N1	MX	stage iia	40	not reported
TCGA-44-A47F	Alive	337	27122	male	T2b	N1	M0	stage iib	32	not reported
TCGA-44-2661	Alive	1159	25313	female	T1	N0	M0	stage ia	None	not reported

TCGA-49-4512	Dead	905	25502	female	T2	N2	MX	stage iiia	None	not reported
TCGA-MP-A4TJ	Dead	339	22875	female	T1	N0	M0	stage ia	None	not reported
TCGA-78-7537	Dead	1622	26610	male	T2	N0	M0	stage ib	40	not reported
TCGA-NJ-A4YI	Dead	4	31867	female	T2	N2	M0	stage iiia	40	not reported
TCGA-86-8668	Alive	423	22383	female	T1b	N0	M0	stage ia	None	not reported
TCGA-55-8506	Alive	11	22850	female	T3	N0	MX	stage iib	None	not reported
TCGA-69-8255	Alive	129	26289	male	T1a	N0	M0	stage ia	None	not reported
TCGA-44-6145	Alive	595	22745	female	T1	N0	M0	stage ia	36	not reported
TCGA-93-A4JN	Alive	718	26273	male	T2a	N0	M1a	stage iv	20	not reported
TCGA-50-5049	Alive	3094	25577	male	T2	N0	M0	stage ia	None	not reported
TCGA-67-6217	Alive	422	26920	female	T2a	N1	M0	stage iia	None	not reported
TCGA-MP-A4TI	Dead	429	26565	male	T2a	N1	M0	stage iia	35	not reported
TCGA-91-A4BC	Alive	44	21565	male	T2b	N0	MX	stage iia	25	not reported
TCGA-86-7711	Dead	1046	25719	male	T2a	N1	M0	stage iia	None	not reported
TCGA-91-6836	Alive	417	19216	female	T2	N0	MX	stage ib	None	not reported
TCGA-49-6744	Alive	1683	23484	female	T2a	N1	MX	stage iia	None	not reported
TCGA-05-5423	Alive	151	23863	male	T2	N1	M0	stage iib	24	not reported
TCGA-95-8494	Alive	84	24773	male	T2a	N1	M0	stage iia	None	not reported
TCGA-99-7458	Alive	747	27359	female	T4	N0	M0	stage iiia	52	not reported
TCGA-97-A4M2	Alive	624	24313	male	T1a	N0	M0	stage ia	None	not reported
TCGA-L9-A50W	Dead	442	27414	male	T1b	N1	MX	stage iia	19	not reported
TCGA-97-A4M5	Alive	634	30665	male	T1b	N0	M0	stage ia	30	not reported
TCGA-69-8254	Alive	409	31134	male	T2b	none	none	not reported	None	not reported
TCGA-55-7573	Alive	487	26302	female	T1b	N0	MX	stage ia	None	not reported
TCGA-55-6971	Alive	1400	21734	female	T2	N0	MX	stage ib	40	not reported
TCGA-75-6212	Dead	1516	None	female	T2	N1	M0	stage iib	None	not reported
TCGA-71-6725	Alive	256	17793	female	T2	N0	M0	stage ib	None	not reported
TCGA-91-6830	Alive	60	23922	female	T1	N1	MX	stage iia	12	not reported
TCGA-78-7161	Dead	291	25294	female	T3	N0	M0	stage iib	37	not reported
TCGA-62-A471	Alive	1246	23689	male	T2b	N1	M0	stage iib	None	not reported
TCGA-05-4424	Alive	913	25689	male	T3	N0	M0	stage iib	50	not reported
TCGA-55-8621	Alive	515	27485	female	T1a	N0	MX	stage ia	30	not reported
TCGA-44-6774	Alive	658	20462	female	T1	N2	M0	stage iiia	None	not reported
TCGA-MP-A4T2	Dead	1136	26010	male	T1	N0	M0	stage ia	50	not reported
TCGA-38-6178	Alive	448	25593	female	T2b	N2	none	stage iiia	None	not reported
TCGA-49-AARQ	Alive	6732	15065	female	T2	N0	MX	stage i	None	not reported
TCGA-62-A46U	Alive	2067	25998	female	T2	N1	M0	stage iib	None	not reported
TCGA-55-1594	Alive	1178	25008	male	T2	N2	M0	stage iiia	None	not reported

TCGA-86-7714	Dead	625	22619	female	T1b	N2	M0	stage iiia	None		not reported
TCGA-44-3398	Alive	1163	28392	female	T1b	N0	M0	stage ia		50	not reported
TCGA-55-A491	Alive	626	29593	female	T1b	N0	MX	stage ia		30	not reported
TCGA-97-7554	Alive	775	30327	female	T2a	N2	M0	stage iiia		38	not reported
TCGA-35-4123	Alive	182	14064	male	T1	N0	M0	stage ia		20	not reported
TCGA-91-8497	Dead	434	27632	female	T1a	N0	MX	stage ia	None		not reported
TCGA-95-7948	Alive	476	15363	female	T2a	N0	M0	stage ib		2	not reported
TCGA-J2-A4AD	Dead	550	22414	female	T1a	N0	MX	stage ia	None		not reported
TCGA-73-4662	Alive	2515	23762	female	T1	N0	M0	stage ia		17	not reported
TCGA-44-6144	Alive	723	21283	male	T1a	N0	M0	stage ia	None		not reported
TCGA-44-2665	Alive	1301	20349	female	T2	N1	M0	stage iib	None		not reported
TCGA-55-5899	Alive	930	None	male	T1a	N1	M0	not reported	None		not reported
TCGA-80-5607	Alive	None	None	female	T2	N1	M0	stage iib		20	not reported
TCGA-86-8076	Alive	993	15605	male	T1	N0	M0	stage ia	None		not reported
TCGA-44-7671	Alive	889	23538	male	T2a	N0	M0	stage ib	None		not reported
TCGA-64-1676	Alive	1728	21428	male	T1a	N0	M0	stage ia	None		not reported
TCGA-97-7937	Alive	564	23919	male	T2a	N0	MX	stage ib	None		not reported
TCGA-MN-A4N1	Alive	827	21939	male	T2a	N1	M0	stage iia	None		not reported
TCGA-78-7542	Dead	321	20763	male	T2	N0	M0	stage ib	None		not reported
TCGA-50-6591	Dead	119	23067	female	T2	N0	M1	stage iv	None		not reported
TCGA-50-5946	Alive	1617	22852	male	T1	N0	MX	stage ia	None		not reported
TCGA-44-6775	Alive	705	26415	female	T2a	N0	MX	stage ib		50	not reported
TCGA-4B-A93V	Dead	300	19035	female	T1b	N0	M0	stage ia		29	not reported
TCGA-MP-A4SW	Dead	1778	19640	male	T2	N1	M0	stage iib		15	not reported
TCGA-55-8299	Dead	469	22626	female	T1b	N0	MX	stage ia		41	not reported
TCGA-35-4122	Alive	225	25491	male	T1	N0	M0	stage ia		45	not reported
TCGA-05-4430	Alive	761	21884	female	T2	N0	M0	stage ib	None		not reported
TCGA-MP-A4T9	Dead	1265	19767	female	T2	N2	MX	stage iiia		24	not reported
TCGA-50-5055	Dead	1830	28988	female	T1	N1	M0	stage iia	None		not reported
TCGA-91-6847	Alive	842	22862	female	T2	N0	MX	stage ib	None		not reported
TCGA-05-4433	Alive	730	30194	male	T2	N0	M0	stage ib		2	not reported
TCGA-55-7727	Alive	119	25929	male	T1a	N2	MX	stage iiia	None		not reported
TCGA-55-7283	Alive	609	27946	female	T3	N2	MX	stage iiia		11	not reported
TCGA-86-8672	Dead	19	21682	male	T3	N0	M0	stage iib	None		not reported
TCGA-86-A4D0	Dead	116	17607	male	T2b	N0	M0	stage iia	None		not reported
TCGA-55-8208	Alive	674	26716	female	T1b	N0	M0	stage ia	None		not reported
TCGA-44-7660	Alive	592	26594	male	T2	N0	MX	stage ib	None		not reported
TCGA-55-6981	Dead	1379	19497	female	T1	N2	M0	stage iiia	None		not reported

TCGA-64-1679	Alive	2488	21310	female	T1	N2	M0	stage iiia	None	not reported
TCGA-50-5072	Dead	250	27036	male	T2	N2	M0	stage iiia	None	not reported
TCGA-55-7816	Dead	468	18170	female	TX	NX	MX	stage iv	None	not reported
TCGA-73-4677	Dead	38	27381	male	T2a	N0	M0	not reported	None	not reported
TCGA-L9-A7SV	Alive	565	25298	male	T2a	N1	M0	stage iia	31	not reported
TCGA-78-8662	Dead	3361	19563	female	T2	N0	M0	stage ib	None	not reported
TCGA-62-A46Y	Dead	414	25905	female	T2	N2	M0	stage iiia	None	not reported
TCGA-L9-A5IP	Dead	58	14681	female	T3	N2	M1b	stage iv	None	not reported
TCGA-86-7954	Alive	605	25062	female	T2	N0	M0	stage ib	8	not reported
TCGA-49-AARN	Dead	1135	20605	female	T1	N0	MX	stage ia	None	not reported
TCGA-55-8512	Dead	607	15266	male	T1a	N1	M1b	stage iv	None	not reported
TCGA-L9-A743	Alive	664	20780	male	T2a	N1	M0	stage iia	None	not reported
TCGA-MN-A4N5	Alive	84	23257	male	T1a	N0	M0	stage ia	40	not reported
TCGA-64-1678	Alive	1189	25931	female	T2b	N0	M0	not reported	20	not reported
TCGA-49-6743	Alive	1621	29807	female	T1	N2	MX	stage iiia	None	not reported
TCGA-05-4396	Dead	303	28094	male	T4	N1	M0	stage iiib	37	not reported
TCGA-05-4250	Dead	121	29068	female	T3	N1	M0	stage iiia	None	not reported
TCGA-44-8120	Alive	260	21188	male	T2a	N0	M0	stage ib	None	not reported
TCGA-05-5420	Alive	457	24472	male	T2	N2	M0	stage iiia	40	not reported
TCGA-69-7973	Alive	230	15355	female	T2a	N0	M0	stage ib	None	not reported
TCGA-99-8032	Alive	44	22635	male	T1a	N0	M0	stage ia	None	not reported
TCGA-75-6214	Dead	1115	None	female	T2	N2	M0	stage iiia	None	not reported
TCGA-91-6831	Alive	310	24436	male	T2	N0	MX	stage ib	None	not reported
TCGA-95-A4VP	Alive	605	24130	female	T2b	N2	M0	stage iiia	40	not reported
TCGA-MP-A5C7	Alive	2248	28015	female	T2	N0	M0	stage ib	15	not reported
TCGA-86-7713	Alive	1157	25791	male	T2b	N0	M0	stage iia	None	not reported
TCGA-64-5774	Alive	2676	22059	male	T2	N0	M0	stage ib	45	not reported
TCGA-44-7659	Alive	691	25730	male	T1b	N0	MX	stage ia	None	not reported
TCGA-NJ-A4YQ	Alive	1432	25427	female	T1b	N0	M0	stage ia	None	not reported
TCGA-62-8398	Dead	444	20197	male	T2	N2	M0	stage iiia	29	not reported
TCGA-78-7154	Dead	593	26481	male	T3	N2	M0	stage iiia	45	not reported
TCGA-86-A4P7	Alive	415	23330	female	T2a	N0	M0	stage ib	None	not reported
TCGA-62-A46O	Dead	1454	24050	female	T2	N0	M0	stage ib	None	not reported
TCGA-MP-A4TC	Dead	74	28471	male	T1	N2	M0	stage iiia	32	not reported
TCGA-05-5429	Dead	275	22066	male	T3	N2	M0	stage iiia	None	not reported
TCGA-91-8496	Alive	505	23358	female	T2a	NX	MX	stage ib	None	not reported
TCGA-55-8620	Dead	375	22091	male	T1a	N1	M1b	stage iv	47	not reported
TCGA-55-7725	Alive	442	24848	female	T1a	N0	MX	stage ia	31	not reported

TCGA-55-1595	Alive	1479	None	female	T1	N0	M0	stage ia	29	not reported
TCGA-38-4631	Dead	354	26538	female	T2	N0	M0	stage ib	None	not reported
TCGA-49-AARO	Alive	3759	14527	female	T1a	N0	MX	stage ia	None	not reported
TCGA-55-8090	Dead	598	29235	male	T1a	N0	M0	stage ia	None	not reported
TCGA-75-6205	Dead	598	None	female	T2a	N0	M0	stage ib	None	not reported
TCGA-91-6835	Alive	79	29887	female	T1	N0	M0	stage ia	None	not reported
TCGA-55-8514	Alive	520	25608	female	T2a	N0	MX	stage ib	40	not reported
TCGA-NJ-A4YF	Alive	2161	18584	female	T1	N0	M0	stage ia	None	not reported
TCGA-75-7027	Alive	3059	None	male	T2	N0	M0	stage ib	None	not reported
TCGA-55-6972	Dead	1632	26625	male	T2	N0	M0	stage ib	None	not reported
TCGA-86-8278	Alive	944	23117	female	T2	N1	M0	stage iib	None	not reported
TCGA-86-8671	Alive	839	26448	female	T2b	N1	M0	stage iib	None	not reported
TCGA-MP-A4SY	Dead	1501	22448	male	T2	N1	M0	stage iib	None	not reported
TCGA-55-7815	Alive	773	28119	male	T2a	N0	MX	stage ib	None	not reported
TCGA-44-A479	Alive	486	27024	female	T2	N0	MX	stage ib	40	not reported
TCGA-55-8505	Alive	440	22676	male	T1a	N2	MX	stage iiia	None	not reported
TCGA-91-8499	Alive	36	27853	female	T1b	N0	MX	stage ia	None	not reported
TCGA-MP-A4TH	Alive	741	25759	female	T1a	N0	M0	stage ia	20	not reported
TCGA-93-7348	Alive	531	27456	female	T1a	N0	MX	stage ia	42	not reported
TCGA-91-7771	Alive	492	22926	male	T3	N0	MX	stage iib	None	not reported
TCGA-86-7701	Alive	947	24209	male	T2	N0	M1	stage iv	None	not reported
TCGA-05-4417	Alive	455	18780	female	T2	N0	M0	stage ib	37	not reported
TCGA-69-7980	Alive	411	25583	female	T1b	N0	M0	stage i	None	not reported
TCGA-05-4384	Alive	426	24411	male	T2	N2	M0	stage iiia	24	not reported
TCGA-95-8039	Alive	830	26317	male	T1	N0	MX	stage ia	None	not reported
TCGA-49-4505	Dead	428	22628	female	T2	N1	M0	stage iib	37	not reported
TCGA-93-8067	Alive	186	28453	male	T2a	N0	MX	stage ib	40	not reported
TCGA-78-7150	Dead	666	21891	male	T2	N1	M0	stage iib	37	not reported
TCGA-67-6216	Alive	141	20967	female	T1a	N0	M0	stage ia	None	not reported
TCGA-50-5939	Dead	460	31236	male	T2	N0	M0	stage ib	None	not reported
TCGA-49-6745	Alive	522	30133	male	T2a	N2	M0	stage iiia	None	not reported
TCGA-97-7938	Dead	18	27980	female	T1a	N0	MX	stage ia	None	not reported
TCGA-55-6982	Dead	995	None	female	T2	N1	M0	stage iib	None	not reported
TCGA-50-5044	Dead	624	26567	female	T4	N1	M0	stage iiib	None	not reported
TCGA-44-4112	Dead	808	22106	female	T2a	N0	M0	stage ib	20	not reported
TCGA-44-6147	Alive	845	24631	female	T1b	NX	M0	stage ia	4	not reported
TCGA-91-6828	Alive	323	25870	male	T1a	N0	M0	stage ia	None	not reported
TCGA-55-8302	Alive	478	20011	male	T2	N0	MX	stage ib	None	not reported

TCGA-05-4382	Alive	607	24868	male	T2	N0	M0	stage ib	None	not reported
TCGA-MP-A4TE	Dead	896	20627	male	T2b	N0	MX	stage iia	40	not reported
TCGA-55-8508	Alive	617	22159	female	T2a	N1	MX	stage iia	None	not reported
TCGA-S2-AA1A	Alive	513	24903	female	T1b	N0	M0	stage ia	38	not reported
TCGA-97-7553	Alive	1870	21518	female	T1	N0	MX	stage ia	None	not reported
TCGA-86-8074	Alive	24	22862	female	T1b	N1	M0	stage iia	None	not reported
TCGA-67-3771	Alive	610	28406	female	T1	N0	M0	stage ia	None	not reported
TCGA-44-A4SU	Dead	409	24820	female	T1a	N0	MX	stage ia	50	not reported
TCGA-97-8177	Alive	499	21648	female	T2a	N0	M0	stage ib	None	not reported
TCGA-86-8585	Alive	353	20918	male	T2a	N0	M0	stage ib	None	not reported
TCGA-78-7153	Alive	3635	23922	female	T2	N0	M0	stage ib	40	not reported
TCGA-49-AAR2	Alive	2224	23479	male	T2	N0	MX	stage ib	None	not reported
TCGA-86-8358	Alive	653	16409	male	T2a	N0	M0	stage ib	None	not reported
TCGA-L4-A4E5	Alive	578	17680	female	T1	N0	M0	stage i	33	not reported
TCGA-86-8359	Dead	444	19088	male	T3	N2	M0	stage iiia	None	not reported
TCGA-44-7661	Dead	557	25483	female	T2a	N0	M0	stage ib	15	not reported
TCGA-55-A57B	Alive	546	29452	female	T1b	N0	M0	stage ia	None	not reported
TCGA-75-7031	Alive	None	None	female	T2	N0	M0	stage ib	10	not reported
TCGA-55-7281	Alive	872	25870	female	T1b	N0	M0	stage ia	None	not reported
TCGA-86-8279	Alive	949	17032	male	T2a	N1	M0	stage iia	None	not reported
TCGA-78-7167	Dead	2681	28295	male	T2	N0	M1	stage iv	None	not reported
TCGA-55-8092	Dead	154	27597	male	T3	N0	MX	stage iib	40	not reported
TCGA-69-8253	Alive	426	21771	female	T1a	N1	MX	stage iia	39	not reported
TCGA-95-A4VN	Alive	553	22927	female	T2a	N1	M0	stage iia	42	not reported
TCGA-86-8075	Dead	694	24402	female	T2	N0	M0	stage ib	None	not reported
TCGA-55-A4DF	Dead	440	32432	male	T1b	N0	MX	stage ia	None	not reported
TCGA-95-7567	Alive	568	22437	male	T2b	N1	M0	stage iib	41	not reported
TCGA-78-7147	Dead	586	24809	female	T2	N1	M0	stage iib	None	not reported
TCGA-44-7670	Alive	882	17392	female	T1b	N1	M0	stage iia	None	not reported
TCGA-55-A493	Alive	28	20027	female	T2a	N0	M0	stage ib	None	not reported
TCGA-50-6594	Dead	370	28924	female	T3	N2	M0	stage iiia	None	not reported
TCGA-44-2656	Alive	1429	21766	male	T2	N0	M0	stage ib	15	not reported
TCGA-75-6206	Alive	2590	None	male	T2	N0	M0	stage ib	None	not reported
TCGA-44-3917	Alive	1183	12179	female	T2	N0	M0	stage ib	None	not reported
TCGA-49-6761	Alive	354	24849	female	T1	N2	MX	stage iiia	None	not reported
TCGA-78-7163	Alive	7248	22002	male	T2	N0	M0	stage ib	41	not reported
TCGA-78-7143	Dead	4961	22673	female	T2	N0	M0	stage ib	None	not reported
TCGA-05-4427	Alive	791	23893	female	T2	N1	M0	stage iib	15	not reported

TCGA-78-7155	Dead	1171	24863	male	T2	N0	M0	stage ib	48	not reported
TCGA-86-6562	Dead	376	19301	male	T2a	N1	M0	stage iia	None	not reported
TCGA-49-4514	Alive	1700	28908	female	T1	N0	M0	stage ia	None	not reported
TCGA-78-7633	Dead	1528	24713	male	T2	N0	M0	stage ib	47	not reported
TCGA-49-6767	Alive	677	17108	female	T3	N0	MX	stage iib	None	not reported
TCGA-55-6985	Alive	1233	21381	female	T2	N0	MX	stage ib	None	not reported
TCGA-J2-8192	Alive	739	23892	female	T2a	N1	MX	stage iia	None	not reported
TCGA-55-7994	Alive	603	29858	male	T3	N0	MX	stage iib	None	not reported
TCGA-49-AARE	Dead	1229	18893	female	T1	N0	MX	stage ia	15	not reported
TCGA-55-A490	Dead	99	28728	male	T2b	N0	MX	stage iia	34	not reported
TCGA-78-7539	Alive	791	27445	female	T2b	N0	M0	stage iia	28	not reported
TCGA-05-4244	Alive	0	25752	male	T2	N2	M1	stage iv	None	not reported
TCGA-95-A4VK	Alive	651	27040	female	T2b	N2	M0	stage iiia	30	not reported
TCGA-55-1592	Dead	701	None	male	T2	N0	M0	stage ia	40	not reported
TCGA-L9-A444	Alive	307	22202	female	T1a	N0	MX	stage ia	None	not reported
TCGA-38-A44F	Alive	133	29534	male	T2a	N0	M0	stage ib	12	not reported
TCGA-78-8640	Alive	7062	21832	male	T1	N1	M0	stage iia	None	not reported
TCGA-67-4679	Alive	448	25202	male	T3	N0	M0	not reported	None	not reported
TCGA-44-A4SS	Alive	415	26991	male	T1b	N0	M0	stage ia	60	not reported
TCGA-67-3773	Alive	427	30706	female	T2	N0	M0	stage ib	None	not reported
TCGA-50-5933	Dead	2393	26377	male	T4	N2	M0	stage iiib	None	not reported
TCGA-71-8520	Dead	210	21921	female	T2	N0	M0	stage ib	None	not reported
TCGA-69-7764	Alive	414	27693	male	T1b	N0	M0	stage ia	14	not reported
TCGA-64-1677	Dead	628	28482	female	T2	N2	M0	stage iiia	None	not reported
TCGA-91-6840	Alive	372	21854	female	T1b	N0	M0	stage ia	25	not reported
TCGA-55-7227	Dead	952	28458	male	T3	N1	MX	stage iiia	40	not reported
TCGA-05-4395	Dead	0	27971	male	T4	N2	M0	stage iiib	None	not reported
TCGA-78-7166	Dead	258	30869	male	T2	N1	M0	stage iib	38	not reported
TCGA-75-7025	Alive	3305	None	male	T2	N0	M0	stage ib	19	not reported
TCGA-44-7667	Alive	1097	18062	female	T3	N0	MX	stage iib	None	not reported
TCGA-05-4418	Dead	274	25417	male	T3	N2	M0	stage iiia	None	not reported
TCGA-55-6984	Dead	760	None	female	T2	N1	M0	stage iib	None	not reported
TCGA-69-7763	Alive	690	25316	male	T1b	N0	M0	stage ia	45	not reported
TCGA-73-4676	Dead	281	16746	male	T2a	N1	M0	stage iia	32	not reported
TCGA-38-4626	Alive	3674	20903	female	T2b	N0	M0	not reported	None	not reported
TCGA-86-8056	Alive	139	23369	female	T4	N0	M0	stage iiia	12	not reported
TCGA-64-5781	Alive	1559	20219	female	T2	N0	M0	stage ib	25	not reported
TCGA-50-6673	Dead	22	30689	female	T1	N0	M0	stage i	None	not reported

TCGA-95-7562	Dead	87	26000	male	T2a	N1	M0	stage iia	10	not reported
TCGA-55-7911	Alive	537	25746	female	T1a	N0	MX	stage ia	22	not reported
TCGA-55-7724	Alive	705	27891	female	T2a	N0	MX	stage ib	50	not reported
TCGA-99-8033	Dead	656	27342	female	TX	NX	M1	stage iv	None	not reported
TCGA-38-4630	Dead	1073	27667	female	T2	N0	M0	stage ib	None	not reported
TCGA-86-8281	Alive	0	27409	male	T1	NX	M0	stage ia	30	not reported
TCGA-69-7760	Alive	202	26777	male	T3	N0	M0	stage iib	None	not reported
TCGA-86-7955	Alive	1072	22772	male	T2a	N0	M0	stage ib	None	not reported
TCGA-55-8085	Alive	904	23596	male	T1b	N0	M0	stage ia	None	not reported
TCGA-55-A494	Alive	481	22540	female	T2a	N0	MX	stage ib	13	not reported
TCGA-69-7761	Alive	186	31041	male	T2a	N0	MX	stage ib	20	not reported
TCGA-44-6779	Dead	500	18469	female	T2	N1	MX	stage iib	30	not reported
TCGA-91-6848	Alive	224	21829	male	T2	N2	MX	stage iiia	None	not reported
TCGA-55-A48X	Alive	689	23341	female	T1b	N1	M0	stage iia	36	not reported
TCGA-44-7662	Alive	218	22543	male	T2a	N0	MX	stage ib	None	not reported
TCGA-86-8055	Dead	124	29124	male	T2a	N1	M0	stage iia	None	not reported
TCGA-55-8513	Alive	791	28284	female	T3	N0	MX	stage iib	None	not reported
TCGA-44-2659	Alive	1367	23808	female	T1	N1	M0	stage iib	38	not reported
TCGA-53-7626	Dead	929	28024	female	T1	N1	M0	stage iia	35	not reported
TCGA-55-8511	Alive	552	26986	female	T2a	N0	MX	stage ib	None	not reported
TCGA-55-A492	Alive	596	25719	female	T1a	N0	MX	stage ia	33	not reported
TCGA-55-7726	Alive	652	26451	female	T1b	N0	MX	stage ia	24	not reported
TCGA-NJ-A4YG	Alive	2261	23812	male	T2	N0	M0	stage ib	52	not reported
TCGA-97-8547	Alive	657	28801	female	T2a	N2	MX	stage iiia	None	not reported
TCGA-69-8453	Alive	813	28413	male	T3	N0	MX	stage iib	None	not reported
TCGA-50-5932	Dead	1235	27454	male	T2	N1	M0	stage iib	None	not reported
TCGA-50-5942	Alive	1847	24781	female	T1	N0	M0	stage ia	None	not reported
TCGA-78-7159	Alive	1974	22261	female	T1	NX	M0	stage ia	42	not reported
TCGA-MN-A4N4	Alive	1175	20888	male	T1b	N0	M0	stage ia	None	not reported
TCGA-95-7039	Alive	1272	19981	female	T3	N0	MX	stage iib	None	not reported
TCGA-49-AAR0	Alive	4765	20932	male	T1	N0	MX	stage ia	None	not reported
TCGA-55-6975	Dead	118	None	male	T2	N1	M0	stage iib	None	not reported
TCGA-55-6983	Alive	2823	None	male	T2	N1	M0	stage iib	None	not reported
TCGA-L4-A4E6	Alive	435	24631	male	T1	N0	M0	stage ia	30	not reported
TCGA-95-7043	Dead	503	23165	female	T1a	N0	MX	stage ia	39	not reported
TCGA-50-6593	Dead	336	18152	female	T1	N2	M0	stage iiia	None	not reported
TCGA-05-4398	Alive	1431	17471	female	T4	N3	M0	stage iiib	None	not reported
TCGA-97-8175	Alive	551	20206	female	T2a	N0	M0	stage ib	None	not reported

TCGA-75-6203	Alive	None	None	female	T2	N2	M0	stage iiia	None		not reported
TCGA-97-7941	Alive	484	26298	female	T1b	N0	MX	stage ia		25	not reported
TCGA-93-A4JO	Dead	33	25898	male	T1a	N0	MX	stage ia		50	not reported
TCGA-64-5815	Alive	866	27280	male	T2	N1	M0	stage iib	None		not reported
TCGA-95-7947	Alive	477	24752	male	T1a	N0	M0	stage ia		50	not reported
TCGA-44-7669	Dead	574	21893	male	T1b	N1	MX	stage iia	None		not reported
TCGA-86-A456	Alive	896	28533	female	T1a	N0	M0	stage ia		35	not reported
TCGA-55-7728	Alive	704	23505	female	T2a	N0	MX	stage ib		16	not reported
TCGA-44-6778	Alive	1864	21725	male	T1	N0	MX	stage ia		30	not reported
TCGA-97-8179	Alive	435	26305	male	T1a	N0	M0	stage ia		30	not reported
TCGA-73-4668	Alive	467	24255	female	T2	N1	M0	stage iib		37	not reported
TCGA-49-4501	Dead	1421	24817	female	T2	N0	M0	stage ib	None		not reported
TCGA-86-8054	Alive	1148	22584	male	T2b	N1	M0	stage iib	None		not reported
TCGA-55-A4DG	Alive	608	26044	male	T1b	N0	MX	stage ia	None		not reported
TCGA-05-4422	Alive	365	24837	male	T2	N0	M0	stage ib	None		not reported
TCGA-38-4629	Dead	864	25104	male	T3	N0	M0	stage iib	None		not reported
TCGA-64-1681	Dead	1167	22525	female	T1	N0	M0	stage ia		20	not reported
TCGA-75-5122	Dead	1167	None	male	T2	N0	M0	stage ib	None		not reported
TCGA-44-2666	Dead	97	15970	male	T2	N0	M0	stage ib	None		not reported
TCGA-50-7109	Dead	308	21979	male	T1	N0	M0	stage ia	None		not reported
TCGA-97-A4M3	Alive	540	25384	female	T1b	N0	M0	stage ia		25	not reported
TCGA-73-7498	Alive	1189	21527	female	T1b	N0	M0	stage ia		28	not reported
TCGA-62-A46R	Dead	1725	20063	female	T2	N0	M0	stage ib	None		not reported
TCGA-55-8615	Alive	446	24786	male	T3	N2	MX	stage iiia	None		not reported
TCGA-44-6148	Alive	704	22138	male	T1b	N0	M0	stage ia		31	not reported
TCGA-44-2655	Alive	1324	23854	female	T1	N0	M0	stage ia		30	not reported
TCGA-62-8399	Alive	2696	22952	male	T2	N2	M0	stage iiia	None		not reported
TCGA-44-2657	Alive	1351	27298	female	T2	NX	M0	stage ib		47	not reported
TCGA-05-4245	Alive	730	29647	male	T2	N2	M0	stage iiia	None		not reported
