# Supplementary material for: Construction of Lymph Node Metastasis-Related Prognostic Model and Analysis of Immune Infiltration Mode in Lung Adenocarcinoma
Source: Comput Math Methods Med. 2022 Jun 29;2022:3887857. doi: 10.1155/2022/3887857 (PMC9274234; doi:10.1155/2022/3887857)
Supplement: Supplementary 2 — Supplementary Table 2. Clinical data of LUAD samples in GSE31210 dataset. [file 3887857.f2.pdf]

Accession	Title	Source name	Tissue	Characteristics	Gender	Smoking status	Bi	Pathologic	Pstage iori	Gene alteration status	Myc	Myc_copy	Cluster	Relapse	Days befor	Months befc	Death	Days befor	Exclude for prognosis analysis due to incomplete resection or adjuvant therapy
GSM773540	KH024	frozen tissue of primary lung tumors	primary lung tumor	age (years): 55	female	Never-smoker	0	II	II	ALK-fusion +	Low	1.263	Cluster1	relapsed	253	8.4333333	dead	437	exclude
GSM773541	KH081	frozen tissue of primary lung tumors	primary lung tumor	age (years): 38	female	Ever-smoker	140	II	II	ALK-fusion +	Low	1.064	Cluster1	relapsed	57	1.9	alive	743	none
GSM773542	KH007	frozen tissue of primary lung tumors	primary lung tumor	age (years): 30	male	Ever-smoker	600	II	II	ALK-fusion +	Low	1.192	Cluster1	not relapsed	2601	86.7	alive	2601	none
GSM773543	KH066	frozen tissue of primary lung tumors	primary lung tumor	age (years): 64	female	Never-smoker	0	II	II	ALK-fusion +	nd	.	Cluster1	not relapsed	1173	39.1	alive	1173	exclude
GSM773544	KH012	frozen tissue of primary lung tumors	primary lung tumor	age (years): 63	female	Never-smoker	0	II	II	ALK-fusion +	Low	0.969	Cluster1	not relapsed	2369	78.966667	alive	2369	none
GSM773545	KH027	frozen tissue of primary lung tumors	primary lung tumor	age (years): 60	female	Never-smoker	0	II	II	ALK-fusion +	Low	1.188	Cluster1	not relapsed	1879	62.633333	alive	1879	none
GSM773546	KH083	frozen tissue of primary lung tumors	primary lung tumor	age (years): 68	female	Never-smoker	0	II	II	ALK-fusion +	Low	0.884	Cluster1	not relapsed	606	20.2	alive	606	exclude
GSM773547	KH119	frozen tissue of primary lung tumors	primary lung tumor	age (years): 58	male	Ever-smoker	780	II	II	ALK-fusion +	Low	1.403	Cluster1	not relapsed	143	4.7666667	dead	300	none
GSM773548	KH244	frozen tissue of primary lung tumors	primary lung tumor	age (years): 63	female	Never-smoker	0	IA	I	ALK-fusion +	Low	1.281	Cluster1	not relapsed	1919	63.966667	alive	1919	exclude
GSM773549	KH189	frozen tissue of primary lung tumors	primary lung tumor	age (years): 56	female	Never-smoker	0	IA	I	ALK-fusion +	Low	1.168	Cluster1	not relapsed	1835	61.166667	alive	1835	none
GSM773550	KH259	frozen tissue of primary lung tumors	primary lung tumor	age (years): 34	female	Ever-smoker	300	IA	I	ALK-fusion +	Low	1.322	Cluster1	not relapsed	1828	60.933333	alive	1828	none
GSM773551	KH020	frozen tissue of primary lung tumors	primary lung tumor	age (years): 75	female	Never-smoker	0	II	II	KRAS mutation +	Low	1.049		relapsed	1359	45.3	dead	1841	none
GSM773552	KH029	frozen tissue of primary lung tumors	primary lung tumor	age (years): 60	male	Ever-smoker	2000	II	II	KRAS mutation +	Low	1.454		relapsed	590	19.666667	dead	639	none
GSM773553	KH014	frozen tissue of primary lung tumors	primary lung tumor	age (years): 46	female	Never-smoker	0	IB	I	KRAS mutation +	Low	0.974		relapsed	438	14.6	dead	1311	none
GSM773554	KH067	frozen tissue of primary lung tumors	primary lung tumor	age (years): 60	female	Never-smoker	0	IB	I	KRAS mutation +	Low	1.149		relapsed	861	28.7	alive	1098	none
GSM773555	KH058	frozen tissue of primary lung tumors	primary lung tumor	age (years): 61	male	Ever-smoker	880	IA	I	KRAS mutation +	Low	0.944		relapsed	538	17.933333	alive	1189	none
GSM773556	KH085	frozen tissue of primary lung tumors	primary lung tumor	age (years): 63	male	Ever-smoker	2300	IA	I	KRAS mutation +	Low	1.074		relapsed	1462	48.733333	alive	3863	none
GSM773557	KH054	frozen tissue of primary lung tumors	primary lung tumor	age (years): 47	male	Ever-smoker	1200	II	II	KRAS mutation +	Low	1.28		not relapsed	1475	49.166667	alive	1475	exclude
GSM773558	KH004	frozen tissue of primary lung tumors	primary lung tumor	age (years): 59	male	Ever-smoker	1230	II	II	KRAS mutation +	Low	1.419		not relapsed	1856	61.866667	alive	1856	none
GSM773559	KH022	frozen tissue of primary lung tumors	primary lung tumor	age (years): 64	male	Ever-smoker	1380	II	II	KRAS mutation +	Low	1.175		not relapsed	3067	102.23333	alive	3067	none
GSM773560	KH068	frozen tissue of primary lung tumors	primary lung tumor	age (years): 60	male	Ever-smoker	820	II	II	KRAS mutation +	Low	0.987		not relapsed	1142	38.066667	alive	1142	none
GSM773561	KH139	frozen tissue of primary lung tumors	primary lung tumor	age (years): 61	male	Never-smoker	0	IB	I	KRAS mutation +	Low	1.184		not relapsed	1825	60.833333	alive	1825	none
GSM773562	KH154	frozen tissue of primary lung tumors	primary lung tumor	age (years): 53	female	Never-smoker	0	IB	I	KRAS mutation +	Low	1.069		not relapsed	1610	53.666667	alive	1610	none
GSM773563	KH129	frozen tissue of primary lung tumors	primary lung tumor	age (years): 53	male	Ever-smoker	360	IB	I	KRAS mutation +	Low	1.133		not relapsed	2014	67.133333	alive	2014	none
GSM773564	KH266	frozen tissue of primary lung tumors	primary lung tumor	age (years): 53	male	Ever-smoker	640	IB	I	KRAS mutation +	Low	1.037		not relapsed	1826	60.866667	alive	1826	none
GSM773565	KH232	frozen tissue of primary lung tumors	primary lung tumor	age (years): 67	male	Never-smoker	0	IB	I	KRAS mutation +	Low	1.148		not relapsed	2191	73.033333	alive	2191	none
GSM773566	KH222	frozen tissue of primary lung tumors	primary lung tumor	age (years): 69	female	Never-smoker	0	IB	I	KRAS mutation +	Low	1.03		not relapsed	3342	111.4	alive	3342	none
GSM773567	KH168	frozen tissue of primary lung tumors	primary lung tumor	age (years): 64	male	Ever-smoker	810	IA	I	KRAS mutation +	Low	0.943		not relapsed	1328	44.266667	alive	1328	none
GSM773568	KH183	frozen tissue of primary lung tumors	primary lung tumor	age (years): 69	female	Never-smoker	0	IA	I	KRAS mutation +	Low	1.029		not relapsed	2925	97.5	alive	2925	none
GSM773569	KH230	frozen tissue of primary lung tumors	primary lung tumor	age (years): 50	female	Never-smoker	0	IA	I	KRAS mutation +	Low	1.073		not relapsed	2880	96	alive	2880	none
GSM773570	KH237	frozen tissue of primary lung tumors	primary lung tumor	age (years): 70	female	Never-smoker	0	IA	I	KRAS mutation +	Low	1.174		not relapsed	1724	57.466667	alive	1724	none
GSM773571	KH026	frozen tissue of primary lung tumors	primary lung tumor	age (years): 57	male	Never-smoker	0	II	II	EGFR/KRAS/ALK -	Low	1.09	Cluster1	relapsed	1638	54.6	alive	2765	none
GSM773572	KH035	frozen tissue of primary lung tumors	primary lung tumor	age (years): 58	male	Ever-smoker	820	II	II	EGFR/KRAS/ALK -	Low	1.177	Cluster1	relapsed	315	10.5	alive	1529	none
GSM773573	KH043	frozen tissue of primary lung tumors	primary lung tumor	age (years): 69	male	Ever-smoker	2040	II	II	EGFR/KRAS/ALK -	Low	1.066	Cluster1	relapsed	99	3.3	dead	221	none
GSM773574	KH077	frozen tissue of primary lung tumors	primary lung tumor	age (years): 61	male	Ever-smoker	840	II	II	EGFR/KRAS/ALK -	Low	1.033	Cluster1	relapsed	100	3.3333333	dead	546	none
GSM773575	KH060	frozen tissue of primary lung tumors	primary lung tumor	age (years): 68	male	Ever-smoker	960	II	II	EGFR/KRAS/ALK -	Low	1.057	Cluster1	relapsed	163	5.4333333	dead	346	none
GSM773576	KH063	frozen tissue of primary lung tumors	primary lung tumor	age (years): 64	female	Never-smoker	0	II	II	EGFR/KRAS/ALK -	Low	0.925	Cluster2	relapsed	196	6.5333333	dead	853	exclude
GSM773577	KH006	frozen tissue of primary lung tumors	primary lung tumor	age (years): 69	female	Never-smoker	0	II	II	EGFR/KRAS/ALK -	Low	1.036	Cluster2	relapsed	723	24.1	dead	2661	none
GSM773578	KH080	frozen tissue of primary lung tumors	primary lung tumor	age (years): 66	female	Never-smoker	0	II	II	EGFR/KRAS/ALK -	Low	0.989	Cluster1	relapsed	676	22.533333	alive	817	exclude
GSM773579	KH036	frozen tissue of primary lung tumors	primary lung tumor	age (years): 68	male	Ever-smoker	980	II	II	EGFR/KRAS/ALK -	Low	1.133	Cluster1	relapsed	250	8.3333333	dead	425	none
GSM773580	KH059	frozen tissue of primary lung tumors	primary lung tumor	age (years): 69	male	Ever-smoker	2100	II	II	EGFR/KRAS/ALK -	Low	1.065	Cluster2	relapsed	1281	42.7	alive	1281	none
GSM773581	KH070	frozen tissue of primary lung tumors	primary lung tumor	age (years): 64	male	Ever-smoker	900	IB	I	EGFR/KRAS/ALK -	Low	0.965	Cluster1	relapsed	297	9.9	alive	1044	none
GSM773582	KH073	frozen tissue of primary lung tumors	primary lung tumor	age (years): 64	male	Ever-smoker	820	IB	I	EGFR/KRAS/ALK -	Low	1.45	Cluster1	relapsed	512	17.066667	alive	565	none
GSM773583	KH048	frozen tissue of primary lung tumors	primary lung tumor	age (years): 66	male	Ever-smoker	740	IB	I	EGFR/KRAS/ALK -	Low	1.092	Cluster1	relapsed	635	21.166667	dead	1059	exclude
GSM773584	KH148	frozen tissue of primary lung tumors	primary lung tumor	age (years): 64	male	Ever-smoker	1290	IB	I	EGFR/KRAS/ALK -	Low	1.157	Cluster1	relapsed	813	27.1	dead	1625	none
GSM773585	KH011	frozen tissue of primary lung tumors	primary lung tumor	age (years): 68	female	Never-smoker	0	IB	I	EGFR/KRAS/ALK -	Low	0.861	Cluster1	relapsed	1387	46.233333	dead	1695	none
GSM773586	KH001	frozen tissue of primary lung tumors	primary lung tumor	age (years): 68	male	Ever-smoker	1720	IB	I	EGFR/KRAS/ALK -	High	2.12	Cluster1	relapsed	1144	38.133333	alive	2976	none
GSM773587	KH049	frozen tissue of primary lung tumors	primary lung tumor	age (years): 63	male	Ever-smoker	800	IB	I	EGFR/KRAS/ALK -	Low	1.081	Cluster1	relapsed	258	8.6	dead	1143	none
GSM773588	KH094	frozen tissue of primary lung tumors	primary lung tumor	age (years): 58	female	Never-smoker	0	IB	I	EGFR/KRAS/ALK -	Low	0.988	Cluster2	relapsed	272	9.0666667	alive	1682	none
GSM773589	KH087	frozen tissue of primary lung tumors	primary lung tumor	age (years): 71	male	Ever-smoker	980	IB	I	EGFR/KRAS/ALK -	Low	1.168	Cluster1	relapsed	1052	35.066667	dead	1242	none
GSM773590	KH090	frozen tissue of primary lung tumors	primary lung tumor	age (years): 52	female	Never-smoker	0	IB	I	EGFR/KRAS/ALK -	Low	0.973	Cluster1	relapsed	780	26	alive	2252	none
GSM773591	KH045	frozen tissue of primary lung tumors	primary lung tumor	age (years): 66	male	Ever-smoker	900	IA	I	EGFR/KRAS/ALK -	Low	0.952	Cluster1	relapsed	622	20.733333	dead	943	none
GSM773592	KH018	frozen tissue of primary lung tumors	primary lung tumor	age (years): 66	female	Never-smoker	0	IA	I	EGFR/KRAS/ALK -	Low	1.121	Cluster1	relapsed	287	9.5666667	dead	1346	none
GSM773593	KH159	frozen tissue of primary lung tumors	primary lung tumor	age (years): 47	female	Never-smoker	0	IA	I	EGFR/KRAS/ALK -	Low	1.245	Cluster1	relapsed	1363	45.433333	dead	1482	none
GSM773594	KH092	frozen tissue of primary lung tumors	primary lung tumor	age (years): 64	male	Never-smoker	0	IA	I	EGFR/KRAS/ALK -	Low	1.007	Cluster2	relapsed	198	6.6	alive	2386	exclude
GSM773595	KH041	frozen tissue of primary lung tumors	primary lung tumor	age (years): 54	male	Never-smoker	0	IA	I	EGFR/KRAS/ALK -	Low	1.212	Cluster2	relapsed	997	33.233333	alive	1560	none
GSM773596	KH132	frozen tissue of primary lung tumors	primary lung tumor	age (years): 67	female	Never-smoker	0	IA	I	EGFR/KRAS/ALK -	Low	0.969	Cluster2	relapsed	656	21.866667	dead	1001	none
GSM773597	KH117	frozen tissue of primary lung tumors	primary lung tumor	age (years): 54	female	Never-smoker	0	II	II	EGFR/KRAS/ALK -	Low	0.849	Cluster1	not relapsed	259	8.6333333	dead	259	none
GSM773598	KH013	frozen tissue of primary lung tumors	primary lung tumor	age (years): 65	male	Ever-smoker	1840	II	II	EGFR/KRAS/ALK -	Low	1.021	Cluster1	not relapsed	2289	76.3	alive	2289	none
GSM773599	KH017	frozen tissue of primary lung tumors	primary lung tumor	age (years): 49	female	Ever-smoker	390	II	II	EGFR/KRAS/ALK -	Low	1.026	Cluster1	not relapsed	1916	63.866667	alive	1916	none
GSM773600	KH003	frozen tissue of primary lung tumors	primary lung tumor	age (years): 62	male	Ever-smoker	920	II	II	EGFR/KRAS/ALK -	Low	1.276	Cluster1	not relapsed	2048	68.266667	alive	2048	exclude
GSM773601	KH046	frozen tissue of primary lung tumors	primary lung tumor	age (years): 61	male	Ever-smoker	840	II	II	EGFR/KRAS/ALK -	Low	1.075	Cluster1	not relapsed	1475	49.166667	alive	1475	exclude
GSM773602	KH050	frozen tissue of primary lung tumors	primary lung tumor	age (years): 57	male	Ever-smoker	1520	II	II	EGFR/KRAS/ALK -	Low	1.02	Cluster2	not relapsed	1539	51.3	alive	1539	none
GSM773603	KH082	frozen tissue of primary lung tumors	primary lung tumor	age (years): 60	male	Ever-smoker	1200	II	II	EGFR/KRAS/ALK -	High	1.614	Cluster1	not relapsed	636	21.2	alive	636	none
GSM773604	KH118	frozen tissue of primary lung tumors	primary lung tumor	age (years): 46	male	Ever-smoker	540	II	II	EGFR/KRAS/ALK -	Low	0.932	Cluster1	not relapsed	318	10.6	alive	1275	none
GSM773605	KH015	frozen tissue of primary lung tumors	primary lung tumor	age (years): 52	male	Ever-smoker	1360	II	II	EGFR/KRAS/ALK -	Low	0.988	Cluster2	not relapsed	2005	66.833333	alive	2005	none
GSM773606	KH016	frozen tissue of primary lung tumors	primary lung tumor	age (years): 54	female	Never-smoker	0	II	II	EGFR/KRAS/ALK -	Low	1.075	Cluster2	not relapsed	1965	65.5	alive	1965	none
GSM773607	KH125	frozen tissue of primary lung tumors	primary lung tumor	age (years): 58	female	Never-smoker	0	IB	I	EGFR/KRAS/ALK -	Low	1.052	Cluster2	not relapsed	2425	80.833333	alive	2425	none
GSM773608	KH160	frozen tissue of primary lung tumors	primary lung tumor	age (years): 64	female	Never-smoker	0	IB	I	EGFR/KRAS/ALK -	Low	1.082	Cluster2	not relapsed	1566	52.2	alive	1566	none
GSM773609	KH171	frozen tissue of primary lung tumors	primary lung tumor	age (years): 65	female	Never-smoker	0	IB	I	EGFR/KRAS/ALK -	Low	1.009	Cluster2	not relapsed	990	33	alive	990	none
GSM773610	KH182	frozen tissue of primary lung tumors	primary lung tumor	age (years): 52	male	Ever-smoker	600	IB	I	EGFR/KRAS/ALK -	Low	1.101	Cluster2	not relapsed	729	24.3	alive	729	none
GSM773611	KH211	frozen tissue of primary lung tumors	primary lung tumor	age (years): 61	male	Never-smoker	0	IB	I	EGFR/KRAS/ALK -	Low	1.028	Cluster2	not relapsed	2354	78.466667	alive	2354	none
GSM773612	KH180	frozen tissue of primary lung tumors	primary lung tumor	age (years): 61	male	Ever-smoker	1260	IB	I	EGFR/KRAS/ALK -	Low	1.048	Cluster2	not relapsed	818	27.266667	alive	818	none
GSM773613	KH212	frozen tissue of primary lung tumors	primary lung tumor	age (years): 62	female	Ever-smoker	760	IB	I	EGFR/KRAS/ALK -	Low	0.984	Cluster1	not relapsed	2009	66.966667	alive	2009	none
GSM773614	KH219	frozen tissue of primary lung tumors	primary lung tumor	age (years): 47	female	Ever-smoker	55	IB	I	EGFR/KRAS/ALK -	Low	0.87	Cluster2	not relapsed	2549	84.966667	alive	2549	none
GSM773615	KH221	frozen tissue of primary lung tumors	primary lung tumor	age (years): 64	male	Ever-smoker	920	IB	I	EGFR/KRAS/ALK -	Low	1.12	Cluster1	not relapsed	3411	113.7	alive	3411	none
GSM773616	KH268	frozen tissue of primary lung tumors	primary lung tumor	age (years): 50	male	Ever-smoker	1085	IB	I	EGFR/KRAS/ALK -	High	2.268	Cluster1	not relapsed	1806	60.2	alive	1806	none
GSM773617	KH123	frozen tissue of primary lung tumors	primary lung tumor	age (years): 66	female	Never-smoker	0	IA	I	EGFR/KRAS/ALK -	Low	0.998	Cluster1	not relapsed	1937	64.566667	alive	1937	none
GSM773618	KH161	frozen tissue of primary lung tumors	primary lung tumor	age (years): 56	male	Never-smoker	0	IA	I	EGFR/KRAS/ALK -	Low	1.012	Cluster2	not relapsed	1520	50.666667	alive	1520	none
GSM773619	KH163	frozen tissue of primary lung tumors	primary lung tumor	age (years): 76	male	Ever-smoker	740	IA	I	EGFR/KRAS/ALK -	Low	0.966	Cluster2	not relapsed	1375	45.833333	alive	1375	none
GSM773620	KH145	frozen tissue of primary lung tumors	primary lung tumor	age (years): 56	female	Never-smoker	0	IA	I	EGFR/KRAS/ALK -	Low	1.09	Cluster2	not relapsed	1756	58.533333	alive	1756	none
GSM773621	KH152	frozen tissue of primary lung tumors	primary lung tumor	age (years): 53	male	Ever-smoker	1400	IA	I	EGFR/KRAS/ALK -	High	4.547	Cluster1	not relapsed	1597	53.233333	alive	1597	none
GSM773622	KH153	frozen tissue of primary lung tumors	primary lung tumor	age (years): 57	male	Never-smoker	0	IA	I	EGFR/KRAS/ALK -	High	1.762	Cluster2	not relapsed	1714	57.133333	alive	1714	none
GSM773623	KH155	frozen tissue of primary lung tumors	primary lung tumor	age (years): 61	female	Never-smoker	0	IA	I	EGFR/KRAS/ALK -	Low	1.138	Cluster2	not relapsed	1637	54.566667	alive	1637	none
GSM773624	KH156	frozen tissue of primary lung tumors	primary lung tumor	age (years): 66	female	Never-smoker	0	IA	I	EGFR/KRAS/ALK -	Low	1.056	Cluster2	not relapsed	1653	55.1	alive	1653	none
GSM773625	KH177	frozen tissue of primary lung tumors	primary lung tumor	age (years): 66	male	Ever-smoker	840	IA	I	EGFR/KRAS/ALK -	Low	0.979	Cluster2	not relapsed	1196	39.866667	alive	1196	none
GSM773626	KH147	frozen tissue of primary lung tumors	primary lung tumor	age (years): 57	male	Never-smoker	0	IA	I	EGFR/KRAS/ALK -	Low	1.188	Cluster2	not relapsed	1702	56.733333	alive	1702	none
GSM773627	KH121	frozen tissue of primary lung tumors	primary lung tumor	age (years): 61	female	Never-smoker	0	IA	I	EGFR/KRAS/ALK -	Low	1.045	Cluster1	not relapsed	2050	68.333333	alive	2050	none
GSM773628	KH172	frozen tissue of primary lung tumors	primary lung tumor	age (years): 63	male	Ever-smoker	720	IA	I	EGFR/KRAS/ALK -	Low	1.051	Cluster1	not relapsed	1256	41.866667	alive	1256	none
GSM773629	KH179	frozen tissue of primary lung tumors	primary lung tumor	age (years): 60	male	Ever-smoker	1230	IA	I	EGFR/KRAS/ALK -	Low	1.039	Cluster2	not relapsed	818	27.266667	alive	818	none
GSM773630	KH069	frozen tissue of primary lung tumors	primary lung tumor	age (years): 59	male	Ever-smoker	1200	IA	I	EGFR/KRAS/ALK -	Low	1.041	Cluster1	not relapsed	1154	38.466667	alive	1154	none
GSM773631	KH228	frozen tissue of primary lung tumors	primary lung tumor	age (years): 55	female	Never-smoker	0	IA	I	EGFR/KRAS/ALK -	Low	0.988	Cluster2	not relapsed	3058	101.93333	alive	3058	none
GSM773632	KH233	frozen tissue of primary lung tumors	primary lung tumor	age (years): 52	male	Ever-smoker	100	IA	I	EGFR/KRAS/ALK -	Low	1.296	Cluster2	not relapsed	1941	64.7	alive	1941	none
GSM773633	KH243	frozen tissue of primary lung tumors	primary lung tumor	age (years): 68	female	Never-smoker	0	IA	I	EGFR/KRAS/ALK -	High	2.13	Cluster1	not relapsed	1596	53.2	alive	1596	none
GSM773634	KH247	frozen tissue of primary lung tumors	primary lung tumor	age (years): 55	male	Ever-smoker	720	IA	I	EGFR/KRAS/ALK -	Low	1.09	Cluster2	not relapsed	2450	81.666667	alive	2450	none

GSM773635	KH256	frozen tissue of primary lung tumors	primary lung tumor	age (years): 64	female	Never-smoker	0	IA	I	EGFR/KRAS/ALK -	Low	1.122	Cluster2	not relapsed	1881	62.7	alive	1881	none
GSM773636	KH261	frozen tissue of primary lung tumors	primary lung tumor	age (years): 51	male	Ever-smoker	680	IA	I	EGFR/KRAS/ALK -	Low	1.308	Cluster1	not relapsed	1637	54.566667	alive	1637	none
GSM773637	KH264	frozen tissue of primary lung tumors	primary lung tumor	age (years): 55	female	Never-smoker	0	IA	I	EGFR/KRAS/ALK -	Low	1.033	Cluster2	not relapsed	2034	67.8	alive	2034	none
GSM773638	KH265	frozen tissue of primary lung tumors	primary lung tumor	age (years): 65	female	Never-smoker	0	IA	I	EGFR/KRAS/ALK -	Low	0.996	Cluster2	not relapsed	2134	71.133333	alive	2134	none
GSM773639	KH002	frozen tissue of primary lung tumors	primary lung tumor	age (years): 60	female	Never-smoker	0	II	II	EGFR mutation +	Low	1.027		relapsed	461	15.366667	dead	779	none
GSM773640	KH057	frozen tissue of primary lung tumors	primary lung tumor	age (years): 58	male	Ever-smoker	60	II	II	EGFR mutation +	Low	1.043		relapsed	313	10.433333	dead	540	none
GSM773641	KH061	frozen tissue of primary lung tumors	primary lung tumor	age (years): 68	male	Ever-smoker	190	II	II	EGFR mutation +	Low	1.483		relapsed	540	18	alive	1317	none
GSM773642	KH031	frozen tissue of primary lung tumors	primary lung tumor	age (years): 65	female	Never-smoker	0	II	II	EGFR mutation +	High	1.655		relapsed	341	11.366667	alive	1980	none
GSM773643	KH008	frozen tissue of primary lung tumors	primary lung tumor	age (years): 66	male	Ever-smoker	940	II	II	EGFR mutation +	Low	1.222		relapsed	631	21.033333	dead	702	none
GSM773644	KH056	frozen tissue of primary lung tumors	primary lung tumor	age (years): 66	male	Ever-smoker	800	II	II	EGFR mutation +	Low	1.176		relapsed	253	8.4333333	dead	564	none
GSM773645	KH021	frozen tissue of primary lung tumors	primary lung tumor	age (years): 62	male	Ever-smoker	1720	II	II	EGFR mutation +	Low	0.966		relapsed	392	13.066667	dead	788	exclude
GSM773646	KH047	frozen tissue of primary lung tumors	primary lung tumor	age (years): 58	female	Ever-smoker	720	II	II	EGFR mutation +	Low	0.851		relapsed	480	16	alive	1463	exclude
GSM773647	KH038	frozen tissue of primary lung tumors	primary lung tumor	age (years): 49	male	Ever-smoker	750	II	II	EGFR mutation +	Low	0.732		relapsed	613	20.433333	dead	1345	none
GSM773648	KH078	frozen tissue of primary lung tumors	primary lung tumor	age (years): 61	male	Ever-smoker	340	II	II	EGFR mutation +	Low	0.999		relapsed	790	26.333333	alive	832	exclude
GSM773649	KH025	frozen tissue of primary lung tumors	primary lung tumor	age (years): 59	female	Never-smoker	0	II	II	EGFR mutation +	Low	1.15		relapsed	825	27.5	alive	2687	none
GSM773650	KH033	frozen tissue of primary lung tumors	primary lung tumor	age (years): 53	female	Never-smoker	0	II	II	EGFR mutation +	Low	1.296		relapsed	538	17.933333	dead	1122	exclude
GSM773651	KH009	frozen tissue of primary lung tumors	primary lung tumor	age (years): 64	male	Ever-smoker	320	II	II	EGFR mutation +	Low	0.791		relapsed	1725	57.5	alive	2668	none
GSM773652	KH032	frozen tissue of primary lung tumors	primary lung tumor	age (years): 67	female	Never-smoker	0	IB	I	EGFR mutation +	High	2.431		relapsed	384	12.8	alive	1936	none
GSM773653	KH074	frozen tissue of primary lung tumors	primary lung tumor	age (years): 69	male	Ever-smoker	690	IB	I	EGFR mutation +	Low	1.034		relapsed	587	19.566667	alive	871	none
GSM773654	KH213	frozen tissue of primary lung tumors	primary lung tumor	age (years): 61	female	Never-smoker	0	IB	I	EGFR mutation +	Low	1.373		relapsed	2282	76.066667	alive	2671	none
GSM773655	KH052	frozen tissue of primary lung tumors	primary lung tumor	age (years): 64	female	Ever-smoker	260	IB	I	EGFR mutation +	Low	0.998		relapsed	832	27.733333	alive	1482	none
GSM773656	KH062	frozen tissue of primary lung tumors	primary lung tumor	age (years): 53	female	Never-smoker	0	IB	I	EGFR mutation +	Low	0.994		relapsed	1126	37.533333	alive	1128	none
GSM773657	KH089	frozen tissue of primary lung tumors	primary lung tumor	age (years): 71	female	Never-smoker	0	IB	I	EGFR mutation +	Low	1.157		relapsed	772	25.733333	dead	1042	none
GSM773658	KH010	frozen tissue of primary lung tumors	primary lung tumor	age (years): 52	male	Never-smoker	0	IB	I	EGFR mutation +	Low	1.07		relapsed	495	16.5	dead	1229	none
GSM773659	KH023	frozen tissue of primary lung tumors	primary lung tumor	age (years): 52	female	Never-smoker	0	IB	I	EGFR mutation +	Low	0.974		relapsed	1242	41.4	dead	2263	none
GSM773660	KH030	frozen tissue of primary lung tumors	primary lung tumor	age (years): 54	female	Never-smoker	0	IA	I	EGFR mutation +	Low	0.93		relapsed	987	32.9	alive	1770	none
GSM773661	KH005	frozen tissue of primary lung tumors	primary lung tumor	age (years): 49	male	Ever-smoker	640	IA	I	EGFR mutation +	Low	1.009		relapsed	827	27.566667	dead	1433	none
GSM773662	KH037	frozen tissue of primary lung tumors	primary lung tumor	age (years): 68	female	Never-smoker	0	IA	I	EGFR mutation +	Low	0.871		relapsed	1047	34.9	alive	1823	none
GSM773663	KH093	frozen tissue of primary lung tumors	primary lung tumor	age (years): 62	female	Never-smoker	0	IA	I	EGFR mutation +	Low	0.906		relapsed	1706	56.866667	alive	1988	none
GSM773664	KH051	frozen tissue of primary lung tumors	primary lung tumor	age (years): 63	female	Ever-smoker	20	IA	I	EGFR mutation +	Low	0.958		relapsed	356	11.866667	alive	1434	exclude
GSM773665	KH053	frozen tissue of primary lung tumors	primary lung tumor	age (years): 63	male	Ever-smoker	880	IA	I	EGFR mutation +	Low	1.172		relapsed	463	15.433333	dead	630	none
GSM773666	KH055	frozen tissue of primary lung tumors	primary lung tumor	age (years): 62	female	Never-smoker	0	IA	I	EGFR mutation +	Low	1.009		relapsed	547	18.233333	alive	1537	none
GSM773667	KH084	frozen tissue of primary lung tumors	primary lung tumor	age (years): 57	female	Ever-smoker	340	IA	I	EGFR mutation +	High	1.653		relapsed	1252	41.733333	dead	1429	none
GSM773668	KH086	frozen tissue of primary lung tumors	primary lung tumor	age (years): 39	female	Ever-smoker	100	IA	I	EGFR mutation +	High	1.745		relapsed	567	18.9	dead	1038	none
GSM773669	KH028	frozen tissue of primary lung tumors	primary lung tumor	age (years): 55	female	Never-smoker	0	II	II	EGFR mutation +	Low	1.005		not relapsed	2486	82.866667	alive	2486	none
GSM773670	KH075	frozen tissue of primary lung tumors	primary lung tumor	age (years): 47	male	Ever-smoker	270	II	II	EGFR mutation +	High	1.665		not relapsed	740	24.666667	alive	740	exclude
GSM773671	KH065	frozen tissue of primary lung tumors	primary lung tumor	age (years): 64	female	Never-smoker	0	II	II	EGFR mutation +	nd	.		not relapsed	1042	34.733333	alive	1042	none
GSM773672	KH064	frozen tissue of primary lung tumors	primary lung tumor	age (years): 60	female	Ever-smoker	540	II	II	EGFR mutation +	Low	1		not relapsed	1267	42.233333	alive	1267	exclude
GSM773673	KH072	frozen tissue of primary lung tumors	primary lung tumor	age (years): 48	female	Never-smoker	0	II	II	EGFR mutation +	Low	1.005		not relapsed	1017	33.9	alive	1017	none
GSM773674	KH071	frozen tissue of primary lung tumors	primary lung tumor	age (years): 60	male	Ever-smoker	860	II	II	EGFR mutation +	Low	1.108		not relapsed	1041	34.7	alive	1041	exclude
GSM773675	KH034	frozen tissue of primary lung tumors	primary lung tumor	age (years): 52	male	Ever-smoker	260	II	II	EGFR mutation +	Low	1.087		not relapsed	1859	61.966667	alive	1859	exclude
GSM773676	KH039	frozen tissue of primary lung tumors	primary lung tumor	age (years): 48	female	Never-smoker	0	II	II	EGFR mutation +	Low	1.019		not relapsed	1717	57.233333	alive	1717	none
GSM773677	KH044	frozen tissue of primary lung tumors	primary lung tumor	age (years): 60	female	Never-smoker	0	II	II	EGFR mutation +	Low	1.128		not relapsed	1494	49.8	alive	1494	none
GSM773678	KH079	frozen tissue of primary lung tumors	primary lung tumor	age (years): 49	male	Ever-smoker	1500	II	II	EGFR mutation +	Low	1.005		not relapsed	754	25.133333	alive	754	none
GSM773679	KH116	frozen tissue of primary lung tumors	primary lung tumor	age (years): 63	male	Ever-smoker	880	II	II	EGFR mutation +	Low	1.003		not relapsed	403	13.433333	alive	2364	none
GSM773680	KH120	frozen tissue of primary lung tumors	primary lung tumor	age (years): 68	male	Ever-smoker	290	IB	I	EGFR mutation +	Low	1.26		not relapsed	1733	57.766667	alive	1733	none
GSM773681	KH133	frozen tissue of primary lung tumors	primary lung tumor	age (years): 58	female	Never-smoker	0	IB	I	EGFR mutation +	Low	1.047		not relapsed	1941	64.7	alive	1941	none
GSM773682	KH181	frozen tissue of primary lung tumors	primary lung tumor	age (years): 68	male	Ever-smoker	260	IB	I	EGFR mutation +	Low	1.121		not relapsed	774	25.8	alive	774	none
GSM773683	KH185	frozen tissue of primary lung tumors	primary lung tumor	age (years): 64	female	Never-smoker	0	IB	I	EGFR mutation +	Low	1.181		not relapsed	2913	97.1	alive	2913	none
GSM773684	KH143	frozen tissue of primary lung tumors	primary lung tumor	age (years): 58	male	Ever-smoker	110	IB	I	EGFR mutation +	Low	1.051		not relapsed	1803	60.1	alive	1803	none
GSM773685	KH135	frozen tissue of primary lung tumors	primary lung tumor	age (years): 62	male	Ever-smoker	90	IB	I	EGFR mutation +	Low	1.108		not relapsed	1912	63.733333	alive	1912	none
GSM773686	KH162	frozen tissue of primary lung tumors	primary lung tumor	age (years): 66	female	Never-smoker	0	IB	I	EGFR mutation +	Low	1.028		not relapsed	1481	49.366667	alive	1481	none
GSM773687	KH173	frozen tissue of primary lung tumors	primary lung tumor	age (years): 68	female	Ever-smoker	700	IB	I	EGFR mutation +	Low	1.079		not relapsed	1231	41.033333	alive	1231	none
GSM773688	KH136	frozen tissue of primary lung tumors	primary lung tumor	age (years): 68	male	Never-smoker	0	IB	I	EGFR mutation +	Low	0.97		not relapsed	1964	65.466667	alive	1964	none
GSM773689	KH215	frozen tissue of primary lung tumors	primary lung tumor	age (years): 72	male	Ever-smoker	280	IB	I	EGFR mutation +	Low	0.949		not relapsed	3352	111.73333	alive	3352	none
GSM773690	KH216	frozen tissue of primary lung tumors	primary lung tumor	age (years): 62	male	Ever-smoker	860	IB	I	EGFR mutation +	Low	1.088		not relapsed	2158	71.933333	alive	2158	none
GSM773691	KH217	frozen tissue of primary lung tumors	primary lung tumor	age (years): 67	female	Never-smoker	0	IB	I	EGFR mutation +	Low	0.915		not relapsed	3466	115.53333	alive	3466	none
GSM773692	KH218	frozen tissue of primary lung tumors	primary lung tumor	age (years): 52	male	Never-smoker	0	IB	I	EGFR mutation +	Low	1.104		not relapsed	3263	108.76667	alive	3263	none
GSM773693	KH122	frozen tissue of primary lung tumors	primary lung tumor	age (years): 67	female	Never-smoker	0	IB	I	EGFR mutation +	Low	0.968		not relapsed	1823	60.766667	alive	1823	none
GSM773694	KH130	frozen tissue of primary lung tumors	primary lung tumor	age (years): 59	female	Never-smoker	0	IB	I	EGFR mutation +	Low	1.088		not relapsed	1856	61.866667	alive	1856	none
GSM773695	KH231	frozen tissue of primary lung tumors	primary lung tumor	age (years): 70	female	Never-smoker	0	IB	I	EGFR mutation +	Low	1.148		not relapsed	2449	81.633333	alive	2449	none
GSM773696	KH257	frozen tissue of primary lung tumors	primary lung tumor	age (years): 59	male	Ever-smoker	840	IB	I	EGFR mutation +	Low	1.356		not relapsed	1859	61.966667	alive	1859	none
GSM773697	KH234	frozen tissue of primary lung tumors	primary lung tumor	age (years): 56	male	Ever-smoker	220	IB	I	EGFR mutation +	Low	1.09		not relapsed	1998	66.6	alive	1998	none
GSM773698	KH140	frozen tissue of primary lung tumors	primary lung tumor	age (years): 69	female	Ever-smoker	170	IA	I	EGFR mutation +	High	2.004		not relapsed	1852	61.733333	alive	1852	none
GSM773699	KH141	frozen tissue of primary lung tumors	primary lung tumor	age (years): 61	female	Ever-smoker	310	IA	I	EGFR mutation +	Low	1.075		not relapsed	1894	63.133333	alive	1894	none
GSM773700	KH142	frozen tissue of primary lung tumors	primary lung tumor	age (years): 54	female	Never-smoker	0	IA	I	EGFR mutation +	Low	1.048		not relapsed	1861	62.033333	alive	1861	none
GSM773701	KH144	frozen tissue of primary lung tumors	primary lung tumor	age (years): 64	female	Never-smoker	0	IA	I	EGFR mutation +	Low	1.005		not relapsed	1669	55.633333	alive	1669	exclude
GSM773702	KH095	frozen tissue of primary lung tumors	primary lung tumor	age (years): 45	female	Never-smoker	0	IA	I	EGFR mutation +	High	1.687		not relapsed	3189	106.3	alive	3189	none
GSM773703	KH126	frozen tissue of primary lung tumors	primary lung tumor	age (years): 55	female	Never-smoker	0	IA	I	EGFR mutation +	Low	1.166		not relapsed	1822	60.733333	alive	1822	none
GSM773704	KH137	frozen tissue of primary lung tumors	primary lung tumor	age (years): 61	female	Never-smoker	0	IA	I	EGFR mutation +	Low	0.973		not relapsed	1809	60.3	alive	1809	none
GSM773705	KH190	frozen tissue of primary lung tumors	primary lung tumor	age (years): 65	female	Never-smoker	0	IA	I	EGFR mutation +	Low	1.09		not relapsed	2552	85.066667	alive	2552	none
GSM773706	KH227	frozen tissue of primary lung tumors	primary lung tumor	age (years): 65	male	Never-smoker	0	IA	I	EGFR mutation +	Low	1.18		not relapsed	2968	98.933333	alive	2968	none
GSM773707	KH271	frozen tissue of primary lung tumors	primary lung tumor	age (years): 47	male	Ever-smoker	170	IA	I	EGFR mutation +	Low	1.004		not relapsed	3066	102.2	alive	3066	none
GSM773708	KH167	frozen tissue of primary lung tumors	primary lung tumor	age (years): 60	female	Never-smoker	0	IA	I	EGFR mutation +	Low	0.706		not relapsed	1412	47.066667	alive	1412	none
GSM773709	KH170	frozen tissue of primary lung tumors	primary lung tumor	age (years): 55	male	Ever-smoker	1400	IA	I	EGFR mutation +	Low	1.024		not relapsed	1317	43.9	alive	1317	none
GSM773710	KH174	frozen tissue of primary lung tumors	primary lung tumor	age (years): 61	female	Never-smoker	0	IA	I	EGFR mutation +	Low	1.073		not relapsed	1180	39.333333	alive	1180	none
GSM773711	KH150	frozen tissue of primary lung tumors	primary lung tumor	age (years): 67	male	Ever-smoker	1470	IA	I	EGFR mutation +	Low	0.911		not relapsed	1688	56.266667	alive	1688	none
GSM773712	KH165	frozen tissue of primary lung tumors	primary lung tumor	age (years): 69	male	Ever-smoker	820	IA	I	EGFR mutation +	Low	1.017		not relapsed	1417	47.233333	alive	1417	none
GSM773713	KH166	frozen tissue of primary lung tumors	primary lung tumor	age (years): 65	female	Never-smoker	0	IA	I	EGFR mutation +	Low	0.987		not relapsed	1426	47.533333	alive	1426	none
GSM773714	KH042	frozen tissue of primary lung tumors	primary lung tumor	age (years): 65	male	Ever-smoker	50	IA	I	EGFR mutation +	Low	1.063		not relapsed	1489	49.633333	alive	1674	none
GSM773715	KH124	frozen tissue of primary lung tumors	primary lung tumor	age (years): 56	female	Never-smoker	0	IA	I	EGFR mutation +	Low	1.024		not relapsed	1848	61.6	alive	1848	none
GSM773716	KH127	frozen tissue of primary lung tumors	primary lung tumor	age (years): 65	female	Ever-smoker	70	IA	I	EGFR mutation +	Low	1.29		not relapsed	1856	61.866667	alive	1856	none
GSM773717	KH131	frozen tissue of primary lung tumors	primary lung tumor	age (years): 56	female	Never-smoker	0	IA	I	EGFR mutation +	Low	0.778		not relapsed	1780	59.333333	alive	1780	none
GSM773718	KH128	frozen tissue of primary lung tumors	primary lung tumor	age (years): 59	male	Never-smoker	0	IA	I	EGFR mutation +	Low	1.454		not relapsed	2084	69.466667	alive	2084	none
GSM773719	KH134	frozen tissue of primary lung tumors	primary lung tumor	age (years): 56	male	Ever-smoker	740	IA	I	EGFR mutation +	High	1.616		not relapsed	1879	62.633333	alive	1879	none
GSM773720	KH138	frozen tissue of primary lung tumors	primary lung tumor	age (years): 63	female	Ever-smoker	860	IA	I	EGFR mutation +	Low	1.028		not relapsed	1114	37.133333	alive	1114	none
GSM773721	KH270	frozen tissue of primary lung tumors	primary lung tumor	age (years): 49	male	Ever-smoker	750	IA	I	EGFR mutation +	Low	0.94		not relapsed	2806	93.533333	alive	2806	none
GSM773722	KH149	frozen tissue of primary lung tumors	primary lung tumor	age (years): 58	female	Never-smoker	0	IA	I	EGFR mutation +	Low	1.049		not relapsed	1663	55.433333	alive	1663	none
GSM773723	KH151	frozen tissue of primary lung tumors	primary lung tumor	age (years): 62	male	Ever-smoker	210	IA	I	EGFR mutation +	Low	1.032		not relapsed	1726	57.533333	alive	1726	none
GSM773724	KH157	frozen tissue of primary lung tumors	primary lung tumor	age (years): 68	female	Never-smoker	0	IA	I	EGFR mutation +	Low	1.052		not relapsed	1629	54.3	alive	1629	none
GSM773725	KH186	frozen tissue of primary lung tumors	primary lung tumor	age (years): 66	female	Never-smoker	0	IA	I	EGFR mutation +	Low	1.004		not relapsed	2179	72.633333	alive	2179	none
GSM773726	KH188	frozen tissue of primary lung tumors	primary lung tumor	age (years): 57	female	Ever-smoker	240	IA	I	EGFR mutation +	Low	1.022		not relapsed	1917	63.9	alive	1917	none
GSM773727	KH164	frozen tissue of primary lung tumors	primary lung tumor	age (years): 57	female	Never-smoker	0	IA	I	EGFR mutation +	Low	0.998		not relapsed	1446	48.2	alive	1446	none
GSM773728	KH175	frozen tissue of primary lung tumors	primary lung tumor	age (years): 52	female	Ever-smoker	135	IA	I	EGFR mutation +	Low	0.857		not relapsed	1162	38.733333	alive	1162	none
GSM773729	KH176	frozen tissue of primary lung tumors	primary lung tumor	age (years): 65	female	Never-smoker	0	IA	I	EGFR mutation +	Low	1.431		not relapsed	1231	41.033333	alive	1231	none
GSM773730	KH178	frozen tissue of primary lung tumors	primary lung tumor	age (years): 65	male	Ever-smoker	820	IA	I	EGFR mutation +	Low	1.172		not relapsed	941	31.366667	alive	941	none

GSM773731	KH158	frozen tissue of primary lung tumors	primary lung tumor	age (years): 50	female	Never-smoker	0	IA	I	EGFR mutation +	Low	0.961	not relapsed	1623	54.1	alive	1623	none
GSM773732	KH184	frozen tissue of primary lung tumors	primary lung tumor	age (years): 53	female	Never-smoker	0	IA	I	EGFR mutation +	Low	1.177	not relapsed	1855	61.833333	alive	1855	none
GSM773733	KH169	frozen tissue of primary lung tumors	primary lung tumor	age (years): 53	female	Never-smoker	0	IA	I	EGFR mutation +	Low	1.196	not relapsed	1404	46.8	alive	1404	none
GSM773734	KH187	frozen tissue of primary lung tumors	primary lung tumor	age (years): 66	female	Ever-smoker	620	IA	I	EGFR mutation +	Low	1.125	not relapsed	2221	74.033333	alive	2221	none
GSM773735	KH146	frozen tissue of primary lung tumors	primary lung tumor	age (years): 62	female	Never-smoker	0	IA	I	EGFR mutation +	Low	1.161	not relapsed	849	28.3	alive	849	none
GSM773736	KH076	frozen tissue of primary lung tumors	primary lung tumor	age (years): 66	female	Never-smoker	0	IA	I	EGFR mutation +	Low	0.955	not relapsed	831	27.7	alive	831	none
GSM773737	KH214	frozen tissue of primary lung tumors	primary lung tumor	age (years): 35	male	Ever-smoker	390	IA	I	EGFR mutation +	Low	0.999	not relapsed	3219	107.3	alive	3219	none
GSM773738	KH220	frozen tissue of primary lung tumors	primary lung tumor	age (years): 59	female	Never-smoker	0	IA	I	EGFR mutation +	Low	1.027	not relapsed	2585	86.166667	alive	2585	none
GSM773739	KH223	frozen tissue of primary lung tumors	primary lung tumor	age (years): 67	female	Never-smoker	0	IA	I	EGFR mutation +	Low	1.141	not relapsed	2442	81.4	alive	2442	none
GSM773740	KH224	frozen tissue of primary lung tumors	primary lung tumor	age (years): 56	female	Ever-smoker	259	IA	I	EGFR mutation +	Low	1.148	not relapsed	1910	63.666667	alive	1910	none
GSM773741	KH225	frozen tissue of primary lung tumors	primary lung tumor	age (years): 62	female	Never-smoker	0	IA	I	EGFR mutation +	Low	1.179	not relapsed	1834	61.133333	alive	1834	none
GSM773742	KH226	frozen tissue of primary lung tumors	primary lung tumor	age (years): 46	female	Never-smoker	0	IA	I	EGFR mutation +	Low	1.267	not relapsed	2918	97.266667	alive	2918	exclude
GSM773743	KH229	frozen tissue of primary lung tumors	primary lung tumor	age (years): 67	female	Never-smoker	0	IA	I	EGFR mutation +	Low	0.944	not relapsed	2285	76.166667	alive	2285	none
GSM773744	KH235	frozen tissue of primary lung tumors	primary lung tumor	age (years): 62	male	Ever-smoker	1680	IA	I	EGFR mutation +	Low	1.111	not relapsed	2149	71.633333	alive	2149	none
GSM773745	KH236	frozen tissue of primary lung tumors	primary lung tumor	age (years): 63	male	Ever-smoker	680	IA	I	EGFR mutation +	Low	1.029	not relapsed	2561	85.366667	alive	2561	none
GSM773746	KH238	frozen tissue of primary lung tumors	primary lung tumor	age (years): 52	female	Ever-smoker	700	IA	I	EGFR mutation +	Low	1.15	not relapsed	1947	64.9	alive	1947	none
GSM773747	KH239	frozen tissue of primary lung tumors	primary lung tumor	age (years): 60	female	Never-smoker	0	IA	I	EGFR mutation +	Low	1.026	not relapsed	1841	61.366667	alive	1841	none
GSM773748	KH240	frozen tissue of primary lung tumors	primary lung tumor	age (years): 47	female	Never-smoker	0	IA	I	EGFR mutation +	Low	1.245	not relapsed	1971	65.7	alive	1971	none
GSM773749	KH241	frozen tissue of primary lung tumors	primary lung tumor	age (years): 56	male	Ever-smoker	760	IA	I	EGFR mutation +	Low	1.079	not relapsed	2409	80.3	alive	2409	none
GSM773750	KH245	frozen tissue of primary lung tumors	primary lung tumor	age (years): 56	female	Never-smoker	0	IA	I	EGFR mutation +	Low	1.155	not relapsed	1624	54.133333	alive	1624	none
GSM773751	KH246	frozen tissue of primary lung tumors	primary lung tumor	age (years): 63	female	Ever-smoker	390	IA	I	EGFR mutation +	Low	1.203	not relapsed	2430	81	alive	2430	none
GSM773752	KH248	frozen tissue of primary lung tumors	primary lung tumor	age (years): 59	male	Ever-smoker	800	IA	I	EGFR mutation +	High	2.173	not relapsed	1856	61.866667	alive	1856	none
GSM773753	KH249	frozen tissue of primary lung tumors	primary lung tumor	age (years): 62	female	Never-smoker	0	IA	I	EGFR mutation +	Low	0.939	not relapsed	1516	50.533333	alive	1516	none
GSM773754	KH250	frozen tissue of primary lung tumors	primary lung tumor	age (years): 50	male	Ever-smoker	700	IA	I	EGFR mutation +	Low	1.224	not relapsed	1819	60.633333	alive	1819	none
GSM773755	KH251	frozen tissue of primary lung tumors	primary lung tumor	age (years): 55	male	Never-smoker	0	IA	I	EGFR mutation +	High	2.889	not relapsed	2413	80.433333	alive	2413	none
GSM773756	KH252	frozen tissue of primary lung tumors	primary lung tumor	age (years): 54	female	Ever-smoker	460	IA	I	EGFR mutation +	Low	1.414	not relapsed	1849	61.633333	alive	1849	none
GSM773757	KH253	frozen tissue of primary lung tumors	primary lung tumor	age (years): 65	male	Never-smoker	0	IA	I	EGFR mutation +	Low	1.082	not relapsed	2181	72.7	alive	2181	none
GSM773758	KH254	frozen tissue of primary lung tumors	primary lung tumor	age (years): 66	male	Ever-smoker	1120	IA	I	EGFR mutation +	Low	1.046	not relapsed	2168	72.266667	alive	2168	none
GSM773759	KH255	frozen tissue of primary lung tumors	primary lung tumor	age (years): 62	female	Never-smoker	0	IA	I	EGFR mutation +	Low	1.418	not relapsed	2154	71.8	alive	2154	none
GSM773760	KH258	frozen tissue of primary lung tumors	primary lung tumor	age (years): 64	female	Never-smoker	0	IA	I	EGFR mutation +	Low	1.035	not relapsed	2073	69.1	alive	2073	none
GSM773761	KH260	frozen tissue of primary lung tumors	primary lung tumor	age (years): 64	female	Never-smoker	0	IA	I	EGFR mutation +	Low	1.19	not relapsed	1994	66.466667	alive	1994	none
GSM773762	KH262	frozen tissue of primary lung tumors	primary lung tumor	age (years): 61	male	Never-smoker	0	IA	I	EGFR mutation +	High	2.335	not relapsed	2137	71.233333	alive	2137	none
GSM773763	KH263	frozen tissue of primary lung tumors	primary lung tumor	age (years): 58	female	Never-smoker	0	IA	I	EGFR mutation +	Low	0.877	not relapsed	1998	66.6	alive	1998	none
GSM773764	KH267	frozen tissue of primary lung tumors	primary lung tumor	age (years): 49	male	Ever-smoker	300	IA	I	EGFR mutation +	Low	1.32	not relapsed	1908	63.6	alive	1908	none
GSM773765	KH269	frozen tissue of primary lung tumors	primary lung tumor	age (years): 58	male	Ever-smoker	620	IA	I	EGFR mutation +	Low	1.11	not relapsed	1660	55.333333	alive	1660	none
