# Supplementary material for: Construction of Lymph Node Metastasis-Related Prognostic Model and Analysis of Immune Infiltration Mode in Lung Adenocarcinoma
Source: Comput Math Methods Med. 2022 Jun 29;2022:3887857. doi: 10.1155/2022/3887857 (PMC9274234; doi:10.1155/2022/3887857)
Supplement: Supplementary 3 — Supplementary Table 3. The 19 prognosis-related genes obtained. [file 3887857.f3.pdf]

gene	HR	z	pvalue
PITX3	1.182906	5.5431667	2.97E-08
RHOV	1.206998	4.8975696	9.70E-07
ABCC12	0.91545	-4.27055	1.95E-05
CYP4B1	0.89528	-4.235778	2.28E-05
IGFBP1	1.088823	4.0325644	5.52E-05
ABCC2	1.101994	3.9775669	6.96E-05
CYP17A1	0.87801	-3.934191	8.35E-05
IGFALS	0.862907	-3.768709	0.0001641
4-Mar	1.146795	3.7669234	0.0001653
SLC14A2	0.917566	-3.70068	0.000215
C10orf99	1.076054	3.6885708	0.0002255
FAIM2	0.869197	-3.675019	0.0002378
GFI1B	0.902716	-3.654268	0.0002579
KRT9	1.08507	3.5424001	0.0003965
KRT76	1.090118	3.4527006	0.000555
ZNF536	0.924442	-3.381236	0.0007216
STC1	1.178354	3.3577408	0.0007858
PAQR9	1.072971	3.3204399	0.0008988
C20orf141	1.069278	3.2938456	0.0009883
