# Supplementary material for: Construction of Lymph Node Metastasis-Related Prognostic Model and Analysis of Immune Infiltration Mode in Lung Adenocarcinoma
Source: Comput Math Methods Med. 2022 Jun 29;2022:3887857. doi: 10.1155/2022/3887857 (PMC9274234; doi:10.1155/2022/3887857)
Supplement: Supplementary 4 — Supplementary Table 4. Screened samples after CIBERSORT score. [file 3887857.f4.pdf]

id	B cells naive	B cells memory	Plasma cells	T cells CD8	T cells CD4 naive	T cells CD4 memory resting	T cells CD4 memory activated	T cells follicular helper	T cells regulatory (Tregs)	T cells gamma delta	NK cells resting	NK cells activated	Monocytes	Macrophages M0	Macrophages M1	Macrophages M2	Dendritic cells resting	Dendritic cells activated	Mast cells resting	Mast cells activated	Eosinophils	Neutrophils
TCGA-05-4249	0.055673951	0	0.07654106	0.01132146	0	0.03567509	0	0.027221203	0.011663742	0	0	0.023499982	0	4.14E-05	0.029279123	0.094206028	0.370603901	0	0.217210758	0	0	0.047062249
TCGA-05-4382	0.032121079	0	0.16438446	0.0358435	0	0.030129358	0.019299963	0	0.060279171	0	0.060971353	0	0	0.133824464	0.061042162	0.202461712	0.107833197	0	0	0.026386311	0	0.06542327
TCGA-05-4384	0.072520427	0	0.03262342	0.03246976	0	0.214513737	0	0	0	0	0.020079468	0.02342665	0.0053194	0.114390198	0.024234638	0.318204868	0.063045709	0.016115125	0.024700906	0	0	0.038355694
TCGA-05-4389	0	0.018983589	0	0.25955751	0	0	0.035769465	0.029338971	0.032738555	0	0	0.070785199	0	0.045586158	0.09118399	0.14949118	0.129100408	0	0.118076773	0	0	0.019388206
TCGA-05-4390	0.08801133	0	0.12971087	0.02976598	0	0.080867452	0.077156912	0	0.00945268	0	0.003131763	0	0	0.366816385	0.028376129	0.109865829	0	0	0.0192639	0	0	0.057580776
TCGA-05-4403	0.091945777	0	0	0.05616672	0	0.042649455	0	0	0.055166645	0.001970608	0.022985561	0	0	0.198562792	0.06962231	0.204143755	0.134277219	0	0.10743672	0	0	0.015072439
TCGA-05-4417	0	0.020291206	0.0945319	0.11339979	0	0.102615427	0.084342505	0.024491616	0.031422671	0.011352013	0	0.010531209	0	0.139655789	0.070879933	0.140803461	0.096116883	0	0.030028304	0	0	0.029537293
TCGA-05-4422	0.09815707	0	0.24403417	0.24413153	0	0.055052476	0.000800607	0.042648323	0.04751993	0	0	0.022991487	0	0.038625164	0.048900599	0.113886857	0.021925517	0	0.006700996	0	0	0.01462528
TCGA-05-4424	0.056740037	0	0.02115689	0.09872657	0	0.127129182	0.002186928	0.022503009	0.021781335	0	0	0.009720194	0	0.147605261	0.086023366	0.218253545	0.038436002	0	0.115567928	0	0	0.034169748
TCGA-05-4426	0.089533755	0	0	0.01374778	0	0.210050225	0	0	0.06018303	0	0	0.014921076	0	0.285567473	0.023187649	0.198119275	0	0	0.012403755	0.018127225	0	0.074158763
TCGA-05-4427	0.072547701	0	0.07057139	0.10252199	0	0.106288081	0.048218483	0.010739962	0	0	0	0.021492426	0.0254502	0.004869528	0.070491183	0.132562781	0.262922884	0	0.047769781	0	0	0.02355358
TCGA-05-4430	0.002047942	0	0.02593941	0.04450426	0	0.047815046	0.037301027	0.031721325	0	0	0	0.030935245	0	0.195300765	0.046824315	0.140594082	0.119119799	0.034307827	0.210844122	0	0	0.032744828
TCGA-05-4433	0.066329266	0	0	0.1005039	0	0.114546595	0.011650007	0.006774924	0.020942315	0	0.008114618	0.002425244	0	0.307098264	0.093466649	0.100237556	0.045327737	0	0.091061986	0	0	0.031520941
TCGA-05-5423	0.025099215	0	0.01482984	0.03849593	0	0.110070818	0.000281698	0.022165954	0.042248043	0.023906577	0	0.036261189	0	0	0.00735811	0.211283547	0.220121084	0.057960346	0.091319039	0	0.03321084	0.065387769
TCGA-38-4625	0.070881933	0	0.07304114	0.12366923	0	0.019029623	0.049659447	0.023079129	0	0	0	0.013860278	0	0.180823544	0.100528977	0.207054568	0	0.002120605	0.103094682	0	0	0.033156842
TCGA-38-4626	0.061740744	0	0.01703578	0.04399672	0	0.147731695	0	0	0	0	0.010244262	0.033127065	0	0.166841077	0	0.26514062	0.004696462	0.039232263	0.086187514	0	0	0.124025798
TCGA-38-4629	0.027188854	0	0.0575146	0.13595248	0	0.102349405	0.067240962	0.00011412	0.00079972	0	0.027294977	0	0	0.10209572	0.083268213	0.266274948	0.050407263	0	0.029645367	0	0	0.049853372
TCGA-38-A44F	0.128888606	0	0.11468268	0.08663981	0	0.071110841	0	0.056766337	0.041939974	0	0.001910682	0.010042638	0	0.061436444	0.102064946	0.073091143	0.081855651	0	0.157433948	0	0	0.012136301
TCGA-44-2655	0.143138539	0	0.11089425	0.02513284	0	0.220275987	0	0.014421522	0.004914086	0	0	0.014013032	0.0003805	0	0.020553076	0.173605804	0.191461615	0.005956098	0.059084315	0	0	0.016168357
TCGA-44-2656	0.056492879	0	0.10250202	0.17425701	0	0.083074074	0.081861685	0.004132508	0	0.031044207	0	0.002912708	0	0.021766323	0.058986845	0.178432526	0.035287977	0.089757566	0.062043669	0	0.00096255	0.016485458
TCGA-44-2659	0.066537575	0	0.12325896	0.14719116	0	0.196595949	0.004965528	0.024724767	0.057120503	0.038154481	0	0.010076394	0	0.027619677	0.058820831	0.089808677	0.027304	0.005976861	0.113339071	0	0	0.008505568
TCGA-44-2661	0.035755938	0	0.093395	0.09400564	0	0.110982606	0	0.043369735	0	0.028914005	0	0.031687396	0.0232849	0	0.059883651	0.167116483	0.116335433	0.009755954	0.166107434	0	0	0.019405806
TCGA-44-2665	0	0	0	0.00137212	0	0.231561408	0.013868397	0	0	0	0	0.01439202	0	0.202450934	0.001077748	0.205458798	0.139567937	0.052021019	0.067307007	0	0	0.070922609
TCGA-44-2666	0.087406127	0	0.06310085	0	0	0.229603634	0	0	0.05661194	0	0	0.050832413	0	0.006853776	0	0.02897502	0.010664742	0.077000442	0.312693706	0	0.07625735	0
TCGA-44-3398	0	0.043610042	0.08972683	0.02216159	0	0.06767482	0.001972788	0	0	0	0.061990115	0	0	0	0.002465157	0.107986575	0.394971999	0.048215396	0.086587669	0.002098607	0.01337544	0.057162972
TCGA-44-3918	0	0	0.1084291	0.10372507	0	0.148246153	0.093368474	0.059986326	0	0	0	0.065863977	0	0.049777897	0.06653125	0.200733135	0.017376812	0	0.075259699	0	0	0.010702113
TCGA-44-3919	0.058909182	0	0.06634109	0.09394491	0	0.138553211	0	0.020903845	0.062012876	0	0.020070651	0	0	0.06207526	0.120980227	0.11167921	0.107393794	0	0.090785182	0.006523172	0	0.039827387
TCGA-44-4112	0.093035853	0	0.08890644	0.00774413	0	0.252727353	0.025370301	0	0	0.019510708	0	0.016187695	0	0.049424428	0.000143146	0.136759295	0.013586778	0.144222081	0.126530755	0	0	0.025851036
TCGA-44-6148	0.086849692	0	0.10504438	0.06871063	0	0.053333799	0	0.032355631	0	0	0	0.027083127	0.0216961	0.003996764	0.001350193	0.159571794	0.030262763	0.003015747	0.390955513	0	0	0.0157739
TCGA-44-6775	0.078461073	0	0.05001426	0	0	0.110594627	0.02032778	0.007614358	0	0	0	0.027787412	0.0026586	0.266725156	0.022484209	0.102465227	0.187568242	0.034647038	0.072524821	0	0.00418688	0.011940316
TCGA-44-6778	0.100962961	0	0.04831268	0.10646028	0	0.205262284	0.003949656	0.085433161	0	0	0	0.018878692	0.0131336	0.071509722	0.116309683	0.113795292	0.074637991	0	0.036848889	0	0.00450514	0
TCGA-44-7660	0.047995246	0	0.0082108	0.10144004	0	0	0	0.066177664	0.059523013	0.007790098	0.122078578	0	0	0.094765255	0.088239901	0.244814695	0.119410427	0	0.039554278	0	0	0
TCGA-44-7667	0.070067814	0	0.06988483	0.04061019	0.010014315	0	0.086535776	0	0	0	0	0.057068795	0	0.414597389	0.054489682	0.155623774	0	0	0.026726787	0	0	0.014380657
TCGA-44-7669	0.06922496	0	0.05296109	0.03021661	0	0.163310676	0.081223305	0.007991008	0	0	0.019202624	0	0	0.051624098	0.106158916	0.175776196	0.161359105	0	0.071354672	0	0	0.009596741
TCGA-44-A479	0.092769142	0	0.22590345	0.07820123	0	0.161221513	0.054042911	0.009564211	0.012448728	0.035851773	0	0	0	0.057525186	0.100377996	0.080786377	0.055658818	0	0.032356905	0	0	0.003291765
TCGA-44-A47A	0.022023934	0	0	0.01902492	0	0.072169648	0	0.049379438	0.06789223	0	0	0.016959307	0	0.12345883	0.011091979	0.036333484	0.382172847	0.107737584	0.084806505	0	0	0.006949296
TCGA-44-A47B	0.098669087	0	0	0.131545	0	0.086779253	0.065257506	0.051854886	0.005710304	0	0	0.028214138	0	0.156571091	0.037534982	0.125110651	0.073073225	0	0.131559956	0	0	0.008119927
TCGA-49-4487	0.135843011	0	0.0271115	0.19723823	0	0.084495127	0.088658175	0	0.050136936	0.019703668	0	0	0	0.225454727	0.084807007	0.059018552	0.003242221	0	0.024290839	0	0	0
TCGA-49-4501	0.122453772	0	0.03501122	0.07988831	0	0.127328949	0	0	0.076383442	0.014071155	0	0.026824553	0	0.033391952	0.042601685	0.145902431	0.064922345	0.018355509	0.191727012	0	0	0.021137668
TCGA-49-6744	0.021293841	0	0.08287592	0.14763275	0	0.153750224	0	0.009177081	0.052089021	0	0	0	0.0042483	0.100505191	0.049076717	0.122528643	0.101651949	0.05467962	0.083487568	0	0	0.017003194
TCGA-49-AAR0	0.109682176	0	0.03362381	0.18390344	0	0.030527194	0.009707584	0.059488104	0.072667808	0	0	0.057913414	0.0105329	0.120071599	0.065902891	0.136273209	0.042317816	0	0.062405291	0	0	0.004982732
TCGA-49-AAR2	0.102229699	0	0.35229046	0.09409922	0	0.090653469	0	0.013915373	0.022467972	0	0	0.081152548	0.0047436	0.076823473	0.038189954	0.04708638	0.013072258	0	0.063275557	0	0	0
TCGA-49-AARQ	0.092748637	0	0.03899287	0.21196997	0	0	0.038650309	0.09599903	0.000871484	0	0.001641133	0.057944471	0	0.136538601	0.197875784	0.026345528	0.018944441	0	0.081477749	0	0	0
TCGA-49-AARR	0.135148847	0	0.04597048	0.17040344	0	0.082786419	0.002119887	0.032043984	0.011071725	0	0	0.042435493	0.0032443	0.030027965	0.007532132	0.212221101	0	0	0.196008263	0	0	0.028985953
TCGA-50-5055	0.046320163	0.101837049	0.12655726	0.16181099	0	0.049063895	0.031533593	0.04320717	0.073646781	0.015519963	0	0	0	0.138591205	0.044394619	0.04955118	0.072909251	0.007033154	0.034531886	0	0	0.003491846
TCGA-50-5066	0.029733567	0	0.18346251	0.12598943	0	0.066139141	0.107811664	0.024302441	0	0.057794837	0	0.014806902	0	0.014599685	0.052762298	0.143288614	0.116090016	0	0.04069986	0	0	0.022519045
TCGA-50-5068	0.022182016	0.028324822	0.1300791	0.10555968	0	0.091673144	0.002149647	0.06930327	0	0.054723697	0	0.04358256	0.0019676	0.014508867	0.10658561	0.126999337	0.156827723	0	0.041342606	0	0	0.004190316
TCGA-50-5935	0.052659391	0	0.07590982	0.04242506	0	0.224727542	0	0.001735363	0.084447919	0.032760408	0	0	0	0.184710404	0.036559201	0.10128152	0.070034579	0.067632029	0.025116768	0	0	0
TCGA-50-5941	0.059286967	0	0.0497735	0.14309729	0	0.054094534	0.018459495	0.083558448	0.041873439	0	0	0.021708758	0	0.113754954	0.129696807	0.10128971	0.020723451	0	0.134584607	0	0	0.02809804
TCGA-50-5942	0.07407132	0	0.15305617	0.0639072	0	0.143364353	0	0.005041203	0.0014756	0	0	0.032822213	0.0051323	0	0.026834828	0.028520874	0.093317677	0	0.372456254	0	0	0
TCGA-50-5944	0.073466377	0	0	0.03127838	0	0.094554814	0	0	0.01615936	0	0	0.021350146	0.0038299	0.099624761	0.019280125	0.261088492	0.047448317	0.004542549	0.313323168	0	0	0.014053612
TCGA-50-6597	0.048801748	0	0.13576284	0.12393962	0	0.081745132	0	0.029169468	0.124678026	0.050573106	0	0.048179323	0	0.019057332	0.098723919	0.060263128	0.106446103	0.011584787	0.051576977	0	0	0.009498492
TCGA-50-7109	0.073309666	0	0.19774039	0.13915171	0	0.073288341	0.044264752	0.036167242	0.014869179	0	0	0.083851287	0.0003572	0.060175829	0.094556313	0.087270085	0.03504083	0	0.059957171	0	0	0
TCGA-50-8457	0.037833884	0	0.23792922	0.10483385	0	0.025292409	0	0.051770725	0.077740103	0.018682377	0	0.003149876	0.0364981	0.038808723	0.053092199	0.096357935	0.064894458	0	0.153116149	0	0	0
TCGA-53-7626	0.045985647	0	0.07531582	0.1512252	0	0.188018112	0	0.074334132	0.025580121	0	0	0.011194773	0.0296608	0.072221105	0.063852718	0.105804615	0.032211343	0	0.122762385	0.001833216	0	0
TCGA-53-7813	0.102427046	0	0.34198685	0.15412307	0	0	0.183843231	0.035542449	0	0	0	0.03978675	0	0.037690831	0.021972919	0	0.005537512	0.011701249	0.049138017	0	0	0.016250079
TCGA-55-1592	0.012083128	0	0.07019217	0.02762938	0	0.128171078	0	0.002031241	0.03085128	0	0.044773609	0.000918679	0.0618555	0.237585072	0.053471578	0.153904486	0.096828125	0	0.079704639	0	0	0
TCGA-55-1594	0.137154648	0	0.12069413	0.18791786	0	0.032075999	0.016349643	0.008040552	0.023932405	0	0.009023916	0.01801336	0	0.071832155	0.087645283	0.069717541	0.158407421	0	0.035201347	0	0	0.023993736
TCGA-55-6543	0.059453007	0	0.06937591	0.01221589	0	0.133283766	0	0	0.003879442	0	0	0.014111903	0	0.188053066	0.041275451	0.273944559	0	0.042252356	0.101901535	0	0	0.060253112
TCGA-55-6642	0.097370171	0	0.35368385	0.03461892	0	0.031092062	0.117875112	0	0.00480216	0.01524956	0.028404068	0	0	0.150222772	0.031734405	0.076753004	0.018644814	0	0.039549094	0	0	0
TCGA-55-6971	0.042574247	0	0.12430566	0.16746435	0	0.059837139	0.078460413	0.029778342	0.023708301	0	0	0.035504998	0	0.174526812	0.079868755	0.078633031	0.006871295	0	0.074390478	0	0	0.02407618
TCGA-55-6983	0.139972519	0.031929977	0.04882458	0.10339987	0	0.190371025	0.007956751	0.05631441	0.024829169	0.014825049	0	0.006503676	0	0.177487017	0.025733456	0.066336071	0.025618068	0	0.079619906	0	0	0.000278456
TCGA-55-6986	0.178371889	0	0	0.08307892	0	0.164615001	0.021609667	0	0.054793299	0	0.020759071	0	0.0229498	0.036022288	0.010083099	0.251217609	0.092912458	0.008790787	0.054796128	0	0	0
TCGA-55-6987	0	0.020759115	0.04095676	0.21708018	0	0.064070319	0.097348699	0.056878115	0	0	0.056323765	0.022140618	0	0.096376313	0.118347498	0.083709681	0.033732298	0	0.063780837	0	0	0.028495802
TCGA-55-7283	0.056166565	0	0	0.13943292	0	0.181044	0.013851747	0.043001184	0	0.002500325	0	0.029363494	0	0.009052321	0.055611433	0.125461484	0.100690411	0.027563443	0.189244082	0	0	0.027016588
TCGA-55-7574	0.15811854	0	0.09562375	0.10943288	0	0.272455865	0.049415503	0	0	0	0.011555573	0	0	0.090390003	0.037576458	0.075484328	0	0.010678681	0.082841657	0	0	0.006426767
TCGA-55-7576	0.105107691	0	0.00446738	0.12089631	0	0.081761475	3.05E-05	0.002012974	0.072632006	0	0	0.038544633	0.0160945	0.100381213	0.053511008	0.161315083	0.104477567	0.027906926	0.108965816	0	0	0.001894893
TCGA-55-7725	0	0.002462356	0	0.01263781	0	0.299606735	0.042315637	0.092408065	0	0.025140202	0.009628642	0	0	0.0016358	0.133052268	0.106032153	0.075775518	0	0.176330867	0	0	0.022973946
TCGA-55-7728	0.014442009	0.038389502	0	0.04471304	0	0.017845096	0	0.042398075	0.049963246	0	0	0.021921038	0	0.4982371	0.023516697	0.16947187	0.0309325	0	0.048169832	0	0	0
TCGA-55-7903	0.115265398	0	0	0.11854689	0	0.035684919	0.077539146	0.031961199	0.050938986	0	0.074836902	0	0	0.125973211	0.073591949	0.079921677	0.03102629	0.031226298	0.147981775	0	0	0.005505361
TCGA-55-7911	0.107025415	0	0.00743521	0.15000496	0	0.102443358	0.142095508	0.066416581	0	0	0.061126867	0	0.0043981	0.022112711	0.056012417	0.119148234	0	0.134792168	0.022871851	0	0	0.004116661
TCGA-55-7994	0.065481556	0	0.01257798	0.16260539	0	0	0.021545711	0.041983581	0.047963892	0	0.087638762	0.013591027	0	0.183893248	0.135486733	0.099585046	0.074426815	0.001751368	0.051058154	0	0	0.000410735
TCGA-55-8085	0.130613503	0	0.07494995	0.19615611	0	0	0.068129564	0.03951308	0.05094282	0	0	0.027122648	0.0075	0.155800591	0.06887502	0.082382218	0.01488455	0.033549992	0.023004865	0	0	0.026575111
TCGA-55-8087	0.079662302	0	0	0.00602827	0	0.130429743	0	0	0.050420891	0	0	0.02814826	0	0	0.01304271	0.051227294	0.426235098	0	0.187204176	0	0	0.027601259
TCGA-55-8092	0.002050719	0.002327235	0.39229985	0.21132034	0	0	0.096781678	0.002119169	0.05525104	0	0	0.008887403	0	0.048767315	0.0853983	0.056964206	0	0	0.029534017	0	0	0.008298727
TCGA-55-8096	0.1287934	0	0.16128857	0.02794062	0	0.015891735	0	0	0.012439332	0	0	0.006883262	0	0.297960322	0.026288995	0.155053832	0.09927191	0	0.038796433	0	0	0.02939159
TCGA-55-8097	0.068032389	0	0.07116098	0.05178453	0	0.174652904	0	0.004229524	0.031138508	0	0	0.053451876	0.0010546	0	0.009272961	0.126180954	0.108808737	0.011697173	0.282432172	0	0	0.006102687
TCGA-55-8203	0.051756246	0.003396733	0.28748867	0.0944703	0	0.075586867	0.047126065	0.043591325	0.044335798	0	0.00455462	0.002370774	0	0.133843783	0.069878804	0.071191961	0.017216391	0	0.025433663	0	0	0.027758001
TCGA-55-8206	0.035753564	0	0.0348281	0.05145894	0	0.075084605	0.004519071	0.009786138	0.011251237	0	0	0.031239426	0.2624237	0	0	0.056300138	0.227858336	0.010675887	0.182458119	0	0.00636272	0
TCGA-55-8207	0.004419738	0.007202237	0	0.08933641	0	0.122340108	0	0	0.044525436	0	0	0.012169317	0.0221597	0.166193556	0.001665332	0.172025137	0.144444681	0.028158561	0.164411947	0	0	0.02094781
TCGA-55-8301	0.036952262	0	0.13947136	0.14242109	0	0	0.052305513	0.016629621	0.075084802	0	0.02913589	0.018918012	0	0.142973975	0.218355236	0.066588239	0.000605715	0	0.053941299	0	0	0.00661698
TCGA-55-8507	0.049761267	0	0.03332158	0.09090302	0	0.079600159	0	0.039519418	0	0	0	0.039925249	0	0.291442094	0.018033111	0.108137829	0	0.022163382	0.104917124	0.068851602	0	0.053424169
TCGA-55-8508	0.048284854	0	0.49589481	0.06083515	0	0	0.004964487	0.01330254	0.044454851	0	0.001103283	0.011470175	0	0.241851113	0.008690296	0.029278208	0	0	0.012593588	0	0	0.027276645
TCGA-55-8510	0.066039148	0	0.01581375	0.12499072	0	0.088602909	0.027334431	0.043836591	0.052845551	0	0.000675759	0.008954987	0	0.092514439	0.085867237	0.202543738	0.054661728	0	0.113382195	0	0	0.021936813
TCGA-55-8513	0.070291769	0	0.05642475	0.00549738	0	0.046978355	0	0.042578109	0.005260345	0	0.012306655	0	0	0.169720744	0.018360502	0.326261321	0.110013787	0.027218396	0.079563012	0	0	0.029524869
TCGA-55-8619	0.070470279	0.02689286	0.03596627	0.17975501	0	0.026565909	0	0.00413325	0.093848371	0	0	0.009960455	0.002649	0.129384418	0.028852621	0.128398834	0.058975797	0.006647124	0.161421181	0	0	0.036078579
TCGA-55-8621	0.016537778	0	0.09530692	0.09070842	0	0.138753753	0.006894519	0.002873185	0	0	0.053377835	0	0.0131314	0.147547702	0.096316244	0.076185314	0.09326921	0	0.158751721	0	0	0.010346005
TCGA-55-A48X	0.187778357	0.052650612	0.09685291	0.04661678	0	0.153529445	0	0.014881998	0.052301808	0	0	0.031330125	0	0.111979748	0.019371006	0.042764135	0.02623986	0	0.158364268	0	0	0.005338954
TCGA-55-A57B	0.081267243	0	0	0.00059371	0	0.216282999	0	0	0.023436491	0	0	0.034887785	0	0.065060476	0.01243422	0.088353554	0.270282097	0.055692105	0.089949695	0	0	0.061759625
TCGA-62-8395	0.182527329	0	0.1008131	0.04876747	0	0.11170336	0.00216483	0.019407006	0.02677301	0	0	0.026098215	0	0.124052159	0.032634349	0.143779166	0.10632222	0	0.074957782	0	0	0
TCGA-62-8402	0	0	0	0.13501646	0	0.018484583	0.097902255	0.028739707	0.054766139	0	0.0199661	0	0	0.251181357	0.237433352	0.059897718	0.041522403	0	0.055089929	0	0	0
TCGA-62-A46V	0.077940585	0	0.00458756	0	0	0.122407475	0	0.004993255	0	0	0	0.05173468	0	0.10276222	0	0.158259981	0.207202254	0.082937031	0.181269953	0	0	0.00590501
TCGA-62-A472	0.079251053	0	0.01646852	0.13374151	0	0.025721348	0.034058164	0.064513666	0	0.000733923	0	0.075839983	0	0.01666616	0.067539612	0.097658074	0.087195178	0.002635479	0.26986766	0	0	0.028109679
TCGA-64-1681	0.013926723	0.015408022	0	0.02260915	0	0.158092631	0	0.003435395	0.111100611	0.015375638	0	0	0.0168928	0.078673935	0.147365087	0.064800751	0.324886601	0.027432632	0	0	0	0
TCGA-64-5779	0.094308709	0	0.46435319	0.06734809	0	0.192867106	0.058018804	0	0.010664771	0.004725017	0.000155989	0	0	0.078197745	0.023404165	0	0	0	0.005956413	0	0	0
TCGA-67-3770	0.036888684	0	0.02943428	0.07981595	0	0.169033102	0.035415956	0	0	0.016022883	0	0.035034898	0.0070523	0	0.03782722	0.247746136	0.055913991	0.028581093	0.178006217	0	0	0.043227283
TCGA-67-3772	0.01747279	0	0.24834948	0.05959676	0	0.093585892	0	0	0.024921948	0.013074764	0.021726256	0	0	0.030076935	0.019919122	0.102784352	0.222055549	0.02477188	0.081699408	0	0	0.039964869
TCGA-67-3774	0.102407005	0	0.11073249	0.0830566	0	0.209259585	0	0.009902899	0.012252532	0	0	0.056378483	0.0083903	0.040494202	0.068039715	0.13744999	0.019171114	0.022946612	0.089797132	0	0	0.029721353
TCGA-67-4679	0.086391754	0	0	0.06148053	0	0.114345355	0	0	0.039856318	0	0	0.019627664	0.013471	0	0.02077291	0.064546849	0.140366392	0	0.421343834	0	0	0.017797398
TCGA-67-6217	0.092134303	0	0.12935875	0.17236639	0	0.15465474	0.012586205	0.002637845	0.025376293	0	0	0.019144138	0	0.010748928	0.043038141	0.061966832	0.137395934	0.019736661	0.090554403	0	0	0.028300443
TCGA-69-7763	0.069826313	0	0.19685197	0.06803192	0	0.192637467	0.005131621	0.008082078	0	0	0	0.026199272	0	0.073528855	0.010055303	0.20635343	0.02313722	0.033392174	0.061741077	0	0	0.025031308
TCGA-71-8520	0.071566333	0	0.00410751	0	0	0.201392307	0	0.024249172	0.036017276	0	0.006064599	0.009013819	0	0.30894966	0.04330256	0.131319804	0.017419171	0.028737218	0.059771619	0	0	0.058088957
TCGA-73-4659	0.096635536	0	0.04605228	0.01623775	0	0.118896773	0.021684699	0	0.006287767	0	0.053160098	0	0	0.01708006	0	0.314020561	0.110979911	0.0287669	0.088248671	0	0	0.081949
TCGA-73-4662	0.042307744	0	0.21292324	0.1199837	0	0.094344558	0.010776246	0.053817824	0.00651253	0	0	0.033667178	0.0113509	0.128448598	0.011065836	0.135992399	0.05705041	0.032623059	0.049135785	0	0	0
TCGA-75-5146	0.0982413	0	0.26562794	0.06464702	0	0.207191294	0	0.015695614	0	0	0	0.038102186	0	0	0.042319665	0.140086656	0.049777541	0.006512726	0.04177013	0	0	0.030027931
TCGA-75-5147	0.086173315	0	0.20465505	0.0732656	0	0.073333269	0	0	0.038304196	0.022021346	0.011677751	0	0	0.027793003	0.062085439	0.118958159	0.139209306	0.001289857	0.083097174	0	0	0.058136542
TCGA-75-6206	0.14745755	0	0.02483609	0.03882975	0	0.049767808	0	0.021601286	0.047726904	0	0.051984155	0	0	0.349498075	0.008748111	0.189656353	0	0.011704442	0.041505506	0	0	0.016683972
TCGA-75-6212	0.119095091	0	0.08791468	0.02947599	0	0.093409958	0	0.014188087	0.02303193	0	0	0.009431629	0	0.0329887	0.007725583	0.087909992	0.174746907	0.009607206	0.278316868	0	0	0.032157387
TCGA-75-7025	0.121912238	0	0.05574703	0.10061173	0	0.12580713	0	0.030389458	0.045195226	0	0	0.05471979	0.0326356	0	0	0.034745528	0.189232221	0.056082781	0.14297367	0	0	0.009947568
TCGA-78-7143	0.070291356	0	0	0.02148395	0	0.153639401	0	0.01898747	0.056893337	0	0	0.064203382	0	0.113703478	0.006011506	0.106247891	0.155313001	0.122907749	0.093231167	0	0	0.017086312
TCGA-78-7147	0.062469844	0	0	0.15309544	0	0	0	0.041181508	0.071758298	0.0019814	0	0.073496153	0	0.080760919	0.219778574	0.096367704	0.031714075	0.013568881	0.153827206	0	0	0
TCGA-78-7152	0.160578861	0	0.02721915	0.16527627	0	0.147895232	0.05390059	0	0.053327761	0	0	0.026860491	0	0.13725985	0.05804638	0.095225691	0.001116016	0	0.073293717	0	0	0
TCGA-78-7162	0.050786823	0	0.04732208	0.13271137	0	0.156956972	0	0.079187981	0.021003053	0	0.021445788	0.020444932	0	0.058396122	0.068597842	0.113328598	0.030372224	0.045650573	0.153795644	0	0	0
TCGA-78-7539	0.072084929	0	0.03022843	0.19846608	0	0.099099239	0.036012006	0.038011053	0.054277941	0	0	0.047307031	0.0045182	0.053537653	0.063626027	0.098540649	0.085771522	0.026538392	0.066287898	0	0	0.025692933

TCGA-78-8640	0.054135508	0	0.13612668	0.15884937	0	0.062326628	0	0.037929993	0.017974579	0	0.065148161	0	0	0.256116098	0.063845167	0.147547812	0	0	0	0	0	0
TCGA-78-8648	0	0.039481756	0.11445067	0.1293637	0	0	0	0.044101615	0.023566852	0.017761838	0	0.03649141	0	0.384824826	0.022840855	0.122322217	0	0	0.057274332	0	0	0.007519929
TCGA-78-8655	0.044418816	0	0.00447404	0.11109712	0	0.039995245	0.020706078	0.020263811	0.062030676	0	0	0.011899371	0	0.298003629	0.075905172	0.186721446	0.019187685	0	0.095753821	0	0	0.009543094
TCGA-78-8660	0.118229306	0	0.04537329	0.13956163	0	0.001154407	0.050622194	0.088926727	0.045152214	0	0.054949432	0.001505483	0	0.197425981	0.064962927	0.131000884	0.004544158	0	0.049125026	0	0	0.007466343
TCGA-80-5611	0	0.036286485	0	0.03728239	0	0.067369971	0	0.020909985	0.065657674	0	0.029195012	0	0.0714676	0.408639901	0.074641717	0.132419	0.012685255	0	0.040016794	0	0.00342819	0
TCGA-86-6851	0.014769587	0	0.14944797	0.11993128	0	0.150404843	0.02561212	0.055951562	0.021344036	0	0.03829411	0.037959301	0	0.090299377	0.099633756	0.110307216	0.033893918	0	0.052150925	0	0	0
TCGA-86-7714	0.062612624	0	0	0.00638209	0	0.137000378	0	0	0.009295108	0	0	0.047257026	0	0	0	0.056463644	0.431893971	0.060069138	0.13938581	0	0	0.049640211
TCGA-86-7953	0.104136162	0	0	0.06304478	0	0.188189172	0.144962268	0.026792393	0.01117121	0	0	0	0.0800376	0.09837239	0.015352774	0.153352237	0.030275588	0.031133467	0	0.031960216	0.00866007	0.012559693
TCGA-86-8055	0.092321136	0	0.12208185	0.01952008	0	0.127782662	0.006169925	0	0.031480076	0	0.006091278	0	0	0.263650645	0.0784883	0.109776938	0.064144033	0	0.031792245	0	0	0.046700833
TCGA-86-8075	0.071743056	0	0.08216333	0	0	0.157169264	0.007869412	0.000757814	0.040759728	0	0.007504962	0	0	0.048761773	0.012350787	0.203190384	0.132983202	0.098715499	0	0.031426786	0	0.104604006
TCGA-86-8076	0.059717429	0	0.17609678	0.19254983	0	0.014224743	0.050771376	0.033585319	0.074962668	0	0	0	0	0.195438728	0.053470251	0.039769613	0.046272074	0	0.056344694	0	0	0.006796496
TCGA-86-8280	0.004185619	0	0.09364677	0.06716671	0	0.072852324	0	0.072448836	0.040909483	0.017227148	0	0.038734522	0	0.119164328	0.116402892	0.11234337	0.082352571	0	0.1410247	0	0	0.021540735
TCGA-86-8668	0.056095641	0	0.15844008	0.0392887	0	0.121871094	0	0.060859731	0.029320476	0	0	0.03852996	0	0.136578884	0.061752036	0.114111813	0.029332566	0	0.145345009	0	0	0.008474009
TCGA-86-8669	0.088843372	0	0.20478988	0.07227055	0	0.125440225	0.011727429	0.015068446	0.047194662	0	0	0.042419343	0.0257227	0.105972665	0.027265959	0.07136754	0.122213702	0.021296769	0.010449788	0	0	0.007956951
TCGA-86-8671	0.033283558	0.030153211	0.0032116	0.13575513	0	0.099347099	0.002270812	0.050511982	0.103424694	0.016510117	0.016642643	0	0	0.206515466	0.119014928	0.07772458	0.030992624	0	0.062871856	0	0	0.011769696
TCGA-86-A456	0.091174366	0	0	0.04455532	0	0.157954643	0.020587091	0.029766247	0	0.010431519	0.006358649	0	0	0.127000907	0.05883106	0.296612796	0	0.114627717	0.000627346	0.029289152	0	0.012183186
TCGA-86-A4JF	0.163846799	0	0.06581745	0.07739356	0	0.104943512	0	0.069802296	0	0	0	0.104904688	0	0.113364284	0.053798499	0.102818731	0.004089857	0.035032269	0.087165917	0	0	0.017022143
TCGA-86-A4P7	0.109041852	0	0.11238943	0.12922186	0	0.094611819	0.004821931	0.042483595	0.015660588	0	0	0.024586733	0	0.083376313	0.017832991	0.196051487	0.011688771	0.001962279	0.139194517	0	0	0.01707583
TCGA-86-A4P8	0.050964383	0	0	0.08490635	0	0.30923615	0	0.003737494	0	0	0.027401	0.022181859	0.0093102	0.143441627	0.052185399	0.174862471	0.022115467	0.001554035	0.092286904	0	0	0.005816666
TCGA-91-6828	0.071585122	0	0.06491052	0.09023924	0	0.21661922	0.032697253	0	0	0	0	0.018927625	0	0	0.060197235	0.22171249	0.165143907	0	0.022424207	0	0	0.035543183
TCGA-91-6829	0.122727769	0	0.02097424	0.05523648	0	0.07374838	0.011604651	0	0.018236013	0	0	0.034126145	0	0.166997927	0.008212205	0.158127845	0.00818153	0	0.287219799	0	0	0.034607019
TCGA-91-6831	0.05361669	0	0.15321219	0.09473737	0	0.099991148	0.04217616	0	0.010280935	0	0.023398369	0.001891912	0	0.266045613	0.050737233	0.146142259	0.001658557	0	0.010899426	0	0	0.045212134
TCGA-91-6835	0.110172596	0	0	0.06658494	0	0.17920316	0.005248813	0.007023145	0.08972512	0.059995951	0	0	0	0.195902615	0.078575487	0.156475674	0.011269619	0	0.03982288	0	0	0
TCGA-91-6840	0.210310485	0	0.05662854	0.13397692	0	0.095835463	0	0.040942405	0.027255098	0	0	0.047254491	0	0.149194378	0.123420123	0.078474294	0.000612424	0	0.03609538	0	0	0
TCGA-91-6849	0.109094654	0	0.18836583	0.080837	0	0	0.003044357	0.026380924	0.016050811	0	0.012551636	0.001105545	0	0.374163603	0	0.108482274	0.005244123	0.046211225	0.002578115	0	0	0.0258899
TCGA-91-7771	0.130695399	0	0.04880973	0.15426394	0	0.266451362	0.004655516	0.00292911	0	0	0	0.038653824	0.0058322	0.020768305	0.08213778	0.134232199	0.057125634	0	0.053445055	0	0	0
TCGA-91-8497	0.085151304	0	0.21280409	0.13719163	0	0.079256751	0	0.036586757	0.054427909	0	0	0.043904667	0.0040393	0.039788042	0.00589794	0.068466852	0.090583523	0.003586134	0.122416875	0	0	0.015898214
TCGA-91-8499	0.046828532	0	0.05987922	0.13905461	0	0.061805615	0.07620515	0.047382713	0	0.021194436	0	0.005300768	0	0.099666269	0.092873831	0.109749068	0.198601102	0	0.039726498	0	0	0.001732188
TCGA-91-A4BD	0	0.047959717	0.0582056	0.02714602	0	0.130423089	0	0	0.061148952	0	0	0.045122656	0.1402204	0.009461455	0	0.203874851	0.042800453	0.03866316	0.194973634	0	0	0
TCGA-93-7347	0.085016677	0	0.01972905	0.16594283	0	0.029546625	0.025176161	0.034909051	0.088244428	0.031269709	0	0.016532368	0	0.203030833	0.054264749	0.143311937	0.023080049	0	0.079945542	0	0	0
TCGA-93-7348	0.131869066	0	0.22178972	0.00444589	0	0.140931043	0.009229618	0.007065747	0.044076439	0	0	0.014625412	0	0.26204032	0.030709219	0.056360392	0.00987459	0.003746947	0.054878705	0	0	0.008356888
TCGA-93-8067	0.132660988	0	0.08921317	0.03283	0	0.105984822	0	0.06176478	0.01434838	0	0.011453865	0.065630421	0	0.146487379	0.07001182	0.132643326	0.081861462	0	0.027990098	0	0	0.027119493
TCGA-93-A4JN	0.157897538	0	0.15442479	0.01067143	0	0.12832051	0	0.009377078	0.078511434	0	0	0.023782041	0	0.121401305	0.019031504	0.097780936	0	0.016921947	0.143488056	0	0	0.038391433
TCGA-93-A4JO	0.101583608	0	0.04804666	0.13645034	0	0.166710288	0	0.011211837	0.047913058	0	0.056820384	0	0	0.129380903	0.097901529	0.151919008	0.02927319	0	0	0.022789205	0	0
TCGA-95-7567	0.024545096	0	0.23065933	0.12353623	0	0.023986581	0.083789308	0	0.049860535	0.001335494	0	0.024693019	0	0.054376116	0.08064652	0.199605293	0.060752582	0.017003262	0.018712475	0	0	0.006498164
TCGA-95-7947	0.136605186	0	0.0724638	0.09615875	0	0.127358247	0.013161678	0.017055355	0.031622277	0	0	0.011393733	0	0.135691018	0.144907208	0.103353779	0.027660558	0	0.071394197	0	0	0.011174217
TCGA-95-8039	0.042657892	0	0	0.01288365	0	0.251669123	0.002970595	0.023941266	0.049722261	0	0	0.041788108	0.0067356	0	0.03717156	0.085432698	0.173653887	0.068692315	0.152885252	0	0	0.049795762
TCGA-97-7546	0.101202791	0	0.05966691	0.07367884	0	0.322007679	0.011644666	0	0	0	0.004217152	0.00570016	0.021363	0.035627813	0.030891013	0.107286636	0.038593906	0.025206611	0.128449019	0	0	0.034463786
TCGA-97-7552	0.070091433	0.033289981	0.11702966	0.29596878	0	0.072522852	0.00012524	0.003468424	0.041563552	0.01205835	0	0.008409178	0	0.13805638	0.105810778	0.039581998	0.018432927	0	0.04359047	0	0	0
TCGA-97-7553	0.082014845	0	0.0077655	0.0805461	0	0.174479925	0.010277359	0.009757291	0	0	0.01201338	0.015491071	0	0.18739943	0.046734519	0.152429581	0.059963744	0	0.097662754	0	0	0.063464494
TCGA-97-7554	0.067406986	0	0.22636306	0.04431539	0	0.037219781	0	0.005803221	0.058781968	0.005405617	0	0	0	0.218545195	0.07060107	0.17021543	0.031543408	0	0.042244532	0	0	0.021554342
TCGA-97-7941	0.198309873	0	0	0.09919262	0	0.049129669	0	0.016877418	0.070216818	0	0.023956173	0	0.0001815	0.060653006	0.020571605	0.180309695	0.100468696	0.00374766	0.176385283	0	0	0
TCGA-97-8171	0.170387252	0	0	0	0	0.094564112	0	0	0	0	0	0.050512677	0	0.323993477	0.003134063	0.296706082	0	0	0.060702338	0	0	0
TCGA-97-8172	0.078616968	0.044273303	0.01226541	0.08651433	0	0.182779122	0	0	0.05539119	0	0	0.018787113	0.0046584	0.08449463	0.02649164	0.126049742	0.028852036	0	0.250826108	0	0	0
TCGA-97-8177	0	0.079423792	0	0.08509997	0	0.061892111	0	0.009436971	0.068402897	0	0.02476533	0	0	0.099018124	0.022284934	0.14663253	0.174990589	0.02461416	0.158484331	0	0	0.044954261
TCGA-97-8552	0.024916716	0.036975194	0	0.07117335	0	0.16765818	0	0.017310489	0.033767788	0	0	0.064724704	0.0510763	0.026740436	0.006401712	0.158984317	0.165162243	0.035564475	0.124945717	0	0	0.014598353
TCGA-97-A4LX	0.066773985	0	0.06976294	0.10006903	0	0.169086904	0.017113545	0.05911499	0.03977699	0	0.008993303	0.001375915	0	0.055990468	0.124140366	0.119278186	0.071863699	0	0.068714383	0	0	0.027945288
TCGA-97-A4M0	0.033430931	0	0.00364717	0.10528776	0	0.27884303	0	0.023385483	0.064142381	0	0	0.056293855	0.0240124	0	0.061122207	0.060343408	0.147965583	0.018874198	0.100268853	0	0	0.022382704
TCGA-97-A4M1	0.049403242	0	0	0.06583165	0	0.056120296	0	0.015678673	0.073226989	0	0.004555654	0.012914221	0.0527142	0.020318643	0.012262591	0.256095141	0.036809417	0.018835695	0.315971764	0	0	0.009261798
TCGA-97-A4M2	0.040321182	0	0.04092837	0.10717253	0	0.078718925	0	0.065817494	0.018874682	0	0	0.03144783	0.0100401	0.187706104	0.032496673	0.128388767	0.049538252	0.002125745	0.185091441	0	0	0.021331932
TCGA-97-A4M5	0.100401726	0	0	0.0761309	0	0.137764601	0	0.014353636	0	0	0.017897882	0.036120381	0	0.118721699	0.044310089	0.233482558	0.009414724	0.009710334	0.175687232	0	0	0.02600424
TCGA-97-A4M6	0.097261674	0	0.00593392	0.06434796	0	0.230989666	0	0.035716525	0.055351673	0	0	0.011451773	0	0.090894551	0.100328235	0.078929257	0.086366154	0.015801809	0.081716871	0	0	0.044909929
TCGA-97-A4M7	0.085160512	0	0.068203	0.04278275	0	0.169802871	0	0.048171105	0.048844013	0	0	0.026308342	0.0021903	0.019642286	0.057431815	0.069825275	0.160664983	0.014444718	0.1577134	0	0	0.028814654
TCGA-99-7458	0.032501386	0	0	0.15873949	0	0.194060607	0.009085402	0.0164195	0.051450338	0	0	0.041232351	0	0.09932597	0.060979157	0.114296459	0.115575456	0.017903891	0.069409932	0	0	0.019020065
TCGA-99-8028	0	0.013187765	0.05713214	0.15508994	0	0.159659553	0.064177696	0	0.025281368	0.047518169	0	0.011013097	0	0.154973603	0.109899349	0.123222793	0.029439175	0	0.048425245	0	0	0.000980104
TCGA-99-8032	0.023275775	0	0.32968674	0.06052636	0	0.114750849	0.021952265	0.010648162	0.110347192	0	0	0	0	0.130414776	0.005863535	0.109744969	0	0.002978097	0.042090067	0	0	0.037721213
TCGA-99-AA5R	0.060109209	0.04933548	0.00846793	0.17483908	0	0.192910148	0	0.062015713	0	0	0	0.083655706	0.0151285	0.149637363	0.063533119	0.088460981	0.027016766	0	0.0207389	0.004151075	0	0
TCGA-J2-8192	0.053804313	0	0.00619329	0.01134111	0	0.245822257	0	0	0	0	0.004868218	0.00131153	0	0.212931402	0.020279978	0.14208589	0.107078367	0.028833369	0.131413084	0	0	0.0340372
TCGA-J2-A4AG	0.205741234	0	0.07113897	0.05494299	0	0.228591825	0.040320927	0	0.032308665	0	0	0.012362795	0.0028404	0.051362966	0.018923831	0.078713172	0.110387532	0.062066784	0.019036174	0.001651412	0	0.009610275
TCGA-L4-A4E6	0.075968791	0	0	0.06517021	0	0.135126849	0	0.023399273	0	0	0.00599844	0.011063855	0	0.155480171	0.037564816	0.274491651	0.101247703	0	0.090510649	0	0	0.023977588
TCGA-L9-A50W	0.119062658	0	0.08004099	0.0181947	0	0.110482457	0	0.05030141	0.026105295	0	0	0.057318673	0	0.018512491	0.040460589	0.013135249	0.140385843	0	0.295441947	0	0	0.030557698
TCGA-MP-A4SY	0.116650078	0	0	0	0	0.078007521	0	0	0.034885149	0	0	0.033856034	0	0.404797366	0.014464805	0.147750799	0.071335475	0	0.070234048	0	0	0.028018726
TCGA-MP-A4T4	0.012056122	0	0.08075067	0.17120102	0	0.138626367	0.017109343	0.07080867	0.012802309	0	0.047277075	0	0	0.132133737	0.127728819	0.140695014	0.020508861	0	0.028302002	0	0	0
TCGA-MP-A4T6	0.135248117	0	0	0.08745664	0	0.10866632	0	0.039256278	0.119159464	0	0.007826203	0	0	0.327253029	0.060551065	0.073680139	0.031105994	0	0.00979675	0	0	0
TCGA-MP-A4T9	0.10232094	0	0	0.1144498	0	0.03062694	0.010323231	0.023596404	0	0	0	0.03775144	0	0.145348958	0.050802317	0.22148735	0.01772092	0.022572539	0.185851192	0	0	0.037147971
TCGA-MP-A4TD	0.140130912	0	0.21968484	0.03405202	0	0.124218556	0	0.019457221	0.039865369	0.00589994	0	0.04076807	0	0.234354745	0	0.095336051	0	0.008880099	0.037352181	0	0	0
TCGA-MP-A4TH	0.11149044	0.069696419	0.15277611	0.15286278	0	0.156415191	0	0.039448594	0.068079198	0	0	0.003766291	0	0.054972569	0.024722442	0.099632679	0.041932984	0.003628882	0.012427122	0	0	0.008148306
TCGA-NJ-A4YF	0.108546545	0	0.09608193	0.07980999	0	0.080685875	0.047499777	0.027131886	0.055666365	0	0.007034503	0.012530107	0	0.364838451	0.041695922	0.065023101	0	0	0	0	0	0.013455546
TCGA-NJ-A4YG	0.043489202	0	0.04016484	0.02165808	0	0.166704089	0	0.078374429	0.030451666	0	0	0.061963288	0.0060922	0.017727234	0.032507374	0.126052146	0.211238315	0.02439869	0.112067081	0	0	0.02711139
TCGA-NJ-A4YQ	0.072669724	0	0.13466979	0.16003677	0	0.065334315	0	0.025671715	0.062120046	0.02840153	0	0.024190524	0	0.24023462	0.077452643	0.084671312	0	0	0.02454701	0	0	0
TCGA-05-4250	0.116561703	0	0.01739961	0.23325212	0	0.079620579	0.10785401	0.035950387	0	0	0	0.009445702	0	0.005901347	0.079811641	0.120474809	0.152747443	0	0.025304801	0	0	0.01567585
TCGA-05-4397	0.095003517	0	0.02103796	0.02396497	0	0.055852687	0.046507185	0.019099111	0	0	0	0.034918894	0	0.035074281	0.092577959	0.236839414	0.09933191	0.101631733	0.068662052	0	0	0.069498336
TCGA-05-4398	0.046976107	0	0.11908502	0.1006701	0	0.052591025	0.065988395	0.034951798	0.02916227	0	0.038115903	0	0	0.060397334	0.092896005	0.21075293	0.074900601	0	0	0.05048169	0.00141696	0.021613865
TCGA-05-4405	0.056540209	0	0.11764307	0.05944412	0	0.013918152	0	0.00883013	0.043242184	0	0	0.006090045	0	0.386143432	0.01672963	0.12815553	0.019076201	0	0.119149829	0	0	0.025037461
TCGA-05-4418	0.094099787	0	0.19763153	0.11741415	0	0.033631583	0.089425802	0.01974942	0.033747514	0	0.021116376	0	0	0.104013301	0.090468948	0.12833307	0.002964409	0.004267965	0.047323923	0	0	0.015812229
TCGA-05-4425	0.021564679	0	0	0.03589908	0	0.149297082	0	0	0.066009247	0	0.062465775	0	0	0.193235618	0.066080189	0.23060247	0.138328687	0	0	0.036517172	0	0
TCGA-05-4432	0.037205356	0	0.16632067	0.13042849	0	0.074275696	0	0.036565575	0	0	0.012067865	0.042352856	0	0.259824985	0.08287276	0.135946094	0.00028032	0	0.011365757	0	0	0.010493577
TCGA-05-4434	0.081226112	0	0.01694077	0.01929113	0	0.175431233	0.052030735	0	0.03087779	0	0.049283879	0	0	0.130838993	0.153095434	0.160846016	0.067245246	0	0.057061831	0	0	0.005830827
TCGA-05-5420	0.02679218	0	0.1553156	0.08400553	0	0.048355873	0.059205783	0.039146201	0	0	0.007841229	9.40E-05	0	0.112356041	0.120894883	0.155344848	0.118943747	0	0.033478829	0	0	0.038225216
TCGA-05-5425	0.125828397	0	0.07092245	0.16141185	0	0.011537218	0.136857176	0.026596326	0	0	0.010980923	0	0	0.043289934	0.107392565	0.124778167	0.051079013	0.010670461	0.072289906	0	0	0.046365615
TCGA-05-5428	0.088354346	0	0.00064247	0.0857714	0	0.035489126	0	0.016859098	0.002979793	0	0.012932811	0	0	0.058446033	0.026484909	0.321931897	0.141719312	0	0.156852059	0	0	0.051536744
TCGA-05-5715	0.038278117	0	0	0.01396871	0	0	0	0.034982778	0.054090249	0.016286827	0.031713713	0	0	0.400993708	0.017902689	0.16820011	0.024346482	0	0.174535332	0	0	0.024701285
TCGA-35-4122	0.072389758	0	0.14037933	0.0914326	0	0.051597319	0.067879406	0.040162825	0	0.031113144	0	0.010991917	0	0.03374389	0.058580867	0.174316067	0.137034529	0.005942492	0.04994365	0	0.00655694	0.02793527
TCGA-35-4123	0.008397687	0	0.01455389	0.37204179	0	0.003272374	0.072557101	0.081519567	0.00697965	0	0	0	0	0.125938532	0.047415411	0.144473769	0.062641821	0	0.038530884	0.013139629	0	0.008537896
TCGA-35-5375	0.08120074	0	0.1502897	0.14234279	0	0.013896343	0.027800698	0.056232819	0.047677386	0.049703579	0	0	0	0.032125004	0.102472072	0.119738021	0.064265801	0.005360537	0.088050401	0	0	0.018844105
TCGA-38-4627	0.028535186	0	0.07964986	0.03791005	0	0.17220025	0.000257162	0	0.00919291	0	0	0.009886791	0.0100069	0.08297178	0.043601203	0.261422861	0.077350478	0	0.124794013	0	0	0.062220562
TCGA-38-4628	0.141451419	0	0	0.00110809	0	0.095615047	0	0.00495993	0.023732918	0	0	0.049482608	0.0126516	0.104123368	0	0.287451433	0.09209341	0.013490706	0.114794948	0	0	0.0590445
TCGA-38-4630	0	0	0	0.14325045	0	0.221652241	0	0.009150468	0.068403045	0	0	0.03010797	0	0.197195609	0.071184082	0.201945885	0	0.046604277	0	0.010505971	0	0
TCGA-38-4632	0.064157992	0	0.02178533	0.07135256	0	0.070996128	0.070446817	0.039352247	0	0.024462086	0.004905933	0	0	0	0.057263894	0.17963259	0.250261832	0.022432567	0.079282714	0	0	0.043667311
TCGA-38-7271	0	0.16284718	0.05610777	0.13769424	0	0.145063298	0.036972432	0	0.074309445	0.051595053	0	0	0	0.115673167	0.022871854	0.083870821	0.022729783	0.014500761	0.072730165	0	0	0.003034026
TCGA-44-2668	0.05956099	0	0	0.07397832	0	0.187166447	0.091278119	0	0	0	0.002945173	0.017808412	0	0.230236981	0.063147475	0.05289902	0	0.010850051	0	0.115380124	0	0.09474889
TCGA-44-3396	0.094610215	0	0.17067736	0.0736479	0	0.093730826	0.03864335	0.005615555	0	0.00063858	0.002753697	0	0	0.222391167	0.061057023	0.115598355	0	0.007834364	0	0.050650753	0	0.062150858
TCGA-44-5643	0.123667111	0	0.01535117	0.13267607	0	0.097622748	0.128087079	0.025243844	0.036190402	0	0.014898644	0	0	0.123293304	0.081382554	0.109733114	0.068273381	0.015634008	0.027946568	0	0	0
TCGA-44-6145	0.094276751	0	0.12809695	0.22982854	0	0.031586447	0.113974001	0	0.007945213	0	0	0.015847195	0	0.058999954	0.088548823	0.063330915	0.011694546	0.02404134	0.102192328	0	0	0.029637003
TCGA-44-6774	0.050436206	0	0.04248477	0.01148606	0	0.234294085	0.02292125	0	0.040628381	0	0.054132356	0	0	0.220128213	0.052688216	0.176762748	0.010375169	0	0.021015567	0.004013704	0	0.058633268
TCGA-44-6779	0	0	0	0.1914018	0	0.152933416	0.049869429	0.001480914	0.041776086	0	0.08200413	0	0	0.074672621	0.152194416	0.158218629	0.011371537	0	0.075711626	0	0	0.0083654
TCGA-44-7661	0.057369385	0	0	0.06285673	0	0.10281955	0.121025385	0.054746129	0.040819603	0	0	0	0.0126027	0.028890308	0.04419402	0.196781842	0.062028416	0.160109341	0.029187086	0	0	0.026569506
TCGA-44-7662	0.049595528	0	0.05764467	0.11211318	0	0.126435795	0.056082641	0	0.007590391	0	0.046763365	0	0	0.208034252	0.048168192	0.231505026	0	0	0.036097745	0	0	0.019969215
TCGA-44-7672	0.030458698	0	0.12629735	0.0937636	0	0.102736636	0.038140899	0	0.062180578	0	0	0.049265195	0	0.126887302	0.078268273	0.127263594	0.072166267	0	0.055689868	0	0	0.036881733
TCGA-44-8117	0.087625597	0	0.0151998	0.05393371	0	0.104779313	0.044564916	0.035714651	0.004892582	0.001635057	0	0.017098412	0	0.301599447	0.062376617	0.149153874	0	0	0.107771563	0	0	0.013654466
TCGA-44-8119	0.087480348	0	0.18726881	0.08724268	0	0.017146112	0.004680275	0.02729646	0	0	0.021664389	0.003796649	0	0.314919164	0.051133141	0.163858138	0	0	0	0.014855771	0	0.018658067
TCGA-44-A47G	0.105021348	0	0.11909224	0.14818377	0	0.083752269	0.087845016	0	0.036628097	0.027212872	0	0	0	0.086015962	0.054420859	0.141642417	0.057294915	0	0.028756687	0	0	0.024133542
TCGA-44-A4SS	0.097264089	0	0	0.14988142	0	0.228712942	0.067418452	0.007477569	0	0	0.006362484	0.028316019	0	0.052979093	0.076269113	0.158010809	0.03442227	0.000292483	0.057338914	0	0	0.035254345
TCGA-44-A4SU	0.114926153	0	0.33967971	0.02721618	0	0.082937344	0	0.058246555	0.054600312	0	0	0.011275148	0	0.12738354	0.057746848	0.007849418	0.04570063	0.016968467	0.043630237	0	0	0.011839462
TCGA-49-4488	0.059511036	0	0.08113503	0.16342514	0	0.077611616	0.045061242	0.009710608	0.045754223	0	0.00641514	0	0	0.159091283	0.075738126	0.09152426	0.145625815	0	0.0333129	0	0	0.006083587
TCGA-49-4494	0.070455455	0	0	0.17032886	0	0	0	0.077184839	0.019047182	0.002749002	0	0.066670271	0.0013438	0.142757158	0.075662962	0.136962091	0.147054511	0.013226697	0.048351486	0	0	0.028205707
TCGA-49-4505	0.030256829	0	0.02430348	0.15934866	0	0.009655791	0	0.090542817	0.041991865	0	0	0.020988301	0.0061418	0.026882711	0.100051505	0.103280334	0.129402372	0.002438921	0.235578652	0	0	0.019135925
TCGA-49-4506	0.09858622	0	0.06095172	0.30041637	0	0	0.180333242	0.012722781	0	0	0.040870019	0	0	0.075836895	0.074312924	0.067148163	0.070094652	0	0.000854775	0	0	0.017872248
TCGA-49-4507	0.04314699	0	0.06291383	0.31394597	0	0	0.123379426	0.024850123	0.037770539	0.012697999	0.063911446	0.05167444	0	0.068727513	0.049876164	0.079234548	5.25E-05	0	0.065358022	0	0	0.002460473
TCGA-49-4512	0.084534084	0	0	0	0	0.136109751	0	0	0.051418746	0	0	0.028296071	0.0396149	0.157961119	0.018918249	0.283671197	0.074997301	0.042720897	0.081757671	0	0	0
TCGA-49-4514	0.12579628	0	0.13261958	0.16242521	0	0	0.128559836	0.05467421	0	0	0	0.067745044	0	0.085225545	0.055842783	0.08889712	0	0.010018478	0.066451828	0	0	0.021744082
TCGA-49-6743	0.123084068	0	0.12117266	0.11273799	0	0.103498081	0.02991219	0	0.015870027	0	0.025256726	0.001938208	0	0.21166504	0.075940494	0.099668444	0	0	0.069122052	0	0	0.010134021
TCGA-49-6745	0.042587625	0	0.00590091	0.1452807	0	0.142406297	0.040937379	0.011614754	0.01470465	0	0	0.015426793	0.0160631	0.126661776	0.09616662	0.112467355	0.06208971	0.049208245	0.08742844	0	0	0.031055622
TCGA-49-6761	0.075845276	0	0	0.18037932	0	0	0.189577174	0.036375675	0	0	0.048523674	0.052245028	0	0.122279559	0.096508822	0.153073803	0.020641414	0	0.02351744	0	0	0.001032815
TCGA-49-6767	0.053344096	0	0	0.15868374	0	0.080174316	0.132986827	0	0.015279953	0.037746372	0.029227438	0	0	0.323701839	0.067918822	0.079316704	0	0	0.021619892	0	0	0

TCGA-49-AAQV	0.17079598	0	0.08731404	0.04600632	0	0.144186344	0.007929393	0.038642379	0.100250218	0	0	0.016867564	0	0.226903296	0.052158738	0.032969639	0.03733713	0.002365436	0.03353287	0	0	0.002740652
TCGA-49-AAR3	0.051800583	0	0.05499527	0.1848256	0	0.046026732	0.07558939	0.043949747	0.025678953	0	0	0.037621681	0	0.200163015	0.084715853	0.124054555	0.057428319	0	0.013150304	0	0	0
TCGA-49-AAR4	0.02694953	0	0.10937443	0.39236496	0	0	0.034019071	0.042501172	0.04276076	0.092050126	0	0	0	0.112277709	0.041931395	0.071545143	0.004043507	0	0.011753593	0	0	0.018428604
TCGA-49-AAR9	0.110734119	0	0.03685509	0.14101662	0	0	0	0.088908955	0.046938863	0	0	0.088110047	0	0.402353881	0.015646283	0	0	0.069436139	0	0	0	0
TCGA-49-AARE	0.129349525	0	0.08169262	0.08726303	0	0.031809715	0.061791099	0.025323585	0.064896843	0	0	0.010313237	0	0.293616526	0.106549645	0.071816332	0	0	0.035577847	0	0	0
TCGA-49-AARO	0.124706035	0	0.05861992	0.04408309	0	0.118223517	0.011542613	0.025246224	0.040570292	0	0.013056571	0.009093323	0	0.24692508	0.023763864	0.084492732	0.089617302	0.048866699	0.015620111	0	0	0.045572625
TCGA-50-5044	0	0	0.07563289	0.17742164	0	0.000492182	0.07780621	0	0.025791516	0	0	0.105953897	0	0.081223765	0.061372981	0.157630594	0.082321051	0.020223542	0.11067502	0	0	0.023454705
TCGA-50-5045	0	0.067227064	0.13421764	0.10532238	0	0.036505463	0.048222427	0.030209504	0.006695809	0.048928962	0	0.033770796	0	0.021463902	0.071316964	0.181825007	0.134317118	0	0.044793631	0	0	0.03518333
TCGA-50-5049	0	0	0.14260561	0.20133627	0	0.056548825	0.062042266	0.009317591	0.006888137	0.000890706	0	0.007566849	0.018666	0.162086607	0.084524413	0.159947288	0.031425015	0	0.048120704	0	0.00803374	0
TCGA-50-5072	0.173014985	0	0.02380252	0.05019694	0	0.109507467	0.005858156	0.038172514	0.08099018	0	0.03848323	0	0.0131455	0.19508142	0.05078475	0.146013335	0	0.045865767	0.019823524	0	0	0.009259742
TCGA-50-5930	0.075781925	0	0.22339224	0.15136957	0	0.042912449	0.059228239	0.018280021	0.027097932	0	0.012083459	0	0	0.17676447	0.06992195	0.108470768	0.020821022	0	0.013875956	0	0	0
TCGA-50-5933	0.034951709	0	0.01553937	0.03238307	0	0.156480479	0.013152339	0	0	0	0.014663412	0	0	0.316482886	0.042001446	0.284764024	0	0	0.029218961	9.77E-05	0	0.060264624
TCGA-50-5936	0.074889841	0	0.0363105	0.02212824	0	0.092056109	0	0.022866915	0.050781858	0	0.010750566	0	0	0.485378522	0	0.108443493	0	0.056195211	0.021778148	0	0	0.018420596
TCGA-50-5939	0.097071083	0	0.19764081	0.00158833	0	0.144842425	0.021043076	0	0.004083791	0	0.006619808	0.004864751	0	0.245164919	0.055831091	0.148906212	0	0	0.017607252	0.009207582	0	0.045528867
TCGA-50-6590	0.038976284	0	0.12924045	0.08682054	0	0.078061597	0.109996404	0	0.010750336	0	0.006176724	0.022720717	0	0.25287861	0.10602908	0.104548557	0.00252422	0	0.040336085	0	0	0.010940394
TCGA-50-6592	0.105446694	0	0.03544981	0.14696468	0	0.03624022	0.182736604	0	0.004405309	0	0.113015984	0	0	0.196227815	0.083929527	0.060742051	0	0	0.023520773	0	0	0.011320535
TCGA-50-6593	0.081067833	0	0.02594903	0.00728452	0	0.026320888	0	0.017003602	0.041278325	0	0	0.013156999	0	0.347368631	0.00406249	0.127870873	0.123811024	0.028308342	0.102366742	0	0	0.05415071
TCGA-50-6594	0.042321576	0	0.06712618	0	0.018174521	0.004482207	0.090267166	0	0	0.005386025	0.168947237	0	0	0.187189052	0	0.2019439	0.067070141	0.095545563	0.016164728	0	0	0.035381702
TCGA-50-6595	0.0413024	0	0.0232226	0.06041238	0	0.084474035	0.106875713	0	0	0	0.05718622	0.012940283	0	0.205712846	0.157550027	0.201429011	0	0.006228926	0.018220556	0	0	0.024445013
TCGA-50-8459	0.030258575	0.006958211	0.07253561	0.04779942	0	0.087159932	0.011542045	0	0.053405377	0	0	0	0.0487865	0.081671415	0.023280571	0.193966457	0.216949765	0	0.084890491	0	0	0.040795595
TCGA-53-7624	0	0	0	0.11973381	0	0	0	0.132315204	0.02427062	0.032072711	0.02405317	0	0	0.211083629	0.215504538	0.15759801	0	0.075293218	0	0	0.00807509	0
TCGA-55-1596	0.131015122	0	0.07060195	0.07779331	0	0.043603015	0.039276216	0.044751221	0.061306285	0	0	0.015701805	0	0.171848548	0.07965043	0.078040515	0	0.10196502	0.072195773	0	0	0.012250791
TCGA-55-5899	0.046944786	0	0.05121862	0.10389799	0	0.062235381	0.034256493	0.043061734	0	0.021208659	0	0.020168792	0	0.298979841	0.066637294	0.195985792	0	0	0.047590304	0	0	0.007814311
TCGA-55-6712	0.14221808	0	0.03825541	0.12046339	0	0.175317016	0.012763444	0	0.022871798	0.034703926	0.003900459	0	0	0.131555535	0.104472983	0.138727796	0	0	0.074750164	0	0	0
TCGA-55-6968	0.04341491	0	0.1258778	0.07358173	0	0.014069013	0	0.04966674	0.013075563	0.032977804	0	0.061054248	0	0.349602554	0.099591413	0.080334457	0.013314631	0	0.043439141	0	0	0
TCGA-55-6970	0.007933921	0	0.1486423	0.06563151	0	0.180166514	0.007605414	0.069984723	0.000795417	0.001205645	0	0	0.0238485	0.122675021	0.093709803	0.157190055	0	0.052610056	0.068001104	0	0	0
TCGA-55-6975	0.094219242	0	0.2576764	0.0502869	0	0.063437898	0	0.050185698	0.009006221	0	0	0.047387227	0	0.100183328	0	0.107724093	0	0.128480923	0.073877727	0	0	0.017534337
TCGA-55-6978	0.054015626	0	0.00241569	0.23373682	0	0.131145833	0.084947331	0.024781393	0.01634315	0	0.031895621	0	0	0.085027328	0.097019192	0.116016622	0.087221853	0	0.025834611	0	0	0.009598933
TCGA-55-6979	0.025020323	0	0.10161555	0.09188382	0	0.070944855	0.048960884	0.04031616	0.055576245	0	0.000178862	0.009465327	0	0.183702347	0.143599919	0.154929051	0.017568776	0	0.041047769	0	0	0.015190105
TCGA-55-6980	0.082888007	0	0.01982376	0.02054667	0	0.267568315	0	0.05590849	0.0324704	0	0	0.021708957	0.0390633	0.020834203	0.037606855	0.032288861	0.231019998	0.072494988	0.050046789	0	0	0.015730404
TCGA-55-6981	0.094459327	0	0.04290352	0	0	0.119057799	0	0.010345419	0.018827015	0	0	0.056555956	0	0.172312611	0	0.057712371	0.11811968	0.063551061	0.229492119	0	0	0.016663119
TCGA-55-6982	0.108041873	0	0.08920352	0.04617927	0	0.056184666	0	0.013115221	0.034498754	0	0.001634153	0.007639571	0	0.214547743	0.131710692	0.133989058	0.108399358	0.012108299	0.020754858	0	0	0.021992962
TCGA-55-6985	0.107340509	0	0.05884128	0.06563723	0	0.135961697	0.06497074	0.029090223	0	0	0.003493995	0.021960151	0	0.18600123	0.084044017	0.085496449	0.015070144	0	0.112491414	0	0	0.029600931
TCGA-55-7227	0.07837095	0	0	0.07070954	0	0.155192907	0.006303266	0	0.087761176	0	0	0.023700314	0	0.130374152	0.088482877	0.113888793	0.182048172	0	0.061977875	0	0	0.001189979
TCGA-55-7281	0.035457249	0	0	0.08745856	0	0.100497212	0	0	0.0416907	0	0	0.062323442	0.0085419	0.058402313	0.099340235	0.13350964	0.31611138	0	0.049048099	0	0	0.007619292
TCGA-55-7284	0.121530445	0	0	0.11487121	0	0.104213957	0	0	0.086531892	0	0.037428169	0	0.0180819	0.173933745	0.035605866	0.177712425	0.036790867	0	0.093299511	0	0	0
TCGA-55-7724	0.066560519	0	0.06721709	0.06139283	0	0.017248693	0.043788963	0.00849864	0	0.057000201	0.008864044	0	0	0.087152138	0.100327687	0.106157896	0.217099636	0.059005949	0.050782756	0	0	0.048902962
TCGA-55-7726	0.083976646	0	0.10788336	0	0	0.283681411	0	0	0	0	0.02929491	0	0	0.088230802	0.043978544	0.046211835	0.260362546	0.000862793	0.00235784	0.03702976	0	0.016129551
TCGA-55-7727	0.154646704	0	0	0.09953097	0	0.047413531	0.100040812	0.040622354	0.008651703	0.051056845	0	0	0	0	0.097890831	0.204071332	0.038025081	0	0.125328018	0	0	0.032721818
TCGA-55-7907	0.115124356	0	0.02378013	0.14960208	0	0.046509561	0	0.045268839	0.06943358	0	0	0.053084691	0	0.129353452	0.120999964	0.133466522	0.032569868	0	0.080806959	0	0	0
TCGA-55-7914	0.063600843	0	0.2452229	0.12717932	0	0.123490444	0.017992021	0.011760484	0.027089038	0.034308633	0	0.012977007	0	0.061169801	0.091348243	0.062548625	0	0	0.12131264	0	0	0
TCGA-55-7995	0.055782768	0	0.00074123	0.15704111	0	0.039791026	0.005849885	0.041921316	0.070647983	0.051261866	0.027543314	0.007557494	0.0002152	0.350631241	0.117394835	0.053012478	0	0	0.020608282	0	0	0
TCGA-55-8089	0.088700024	0	0.05658191	0.17572697	0	0	0.108635928	0.025933836	0	0	0.010166018	0.0484795	0	0.073339312	0.143696687	0.120362664	0.064783282	0	0.073872379	0	0	0.00972149
TCGA-55-8091	0.051348485	0	0.06371586	0.00554888	0	0.201939662	0	0	0.001670383	0	0.002784867	0.036620737	0	0.081256581	0.065056328	0.146319584	0.131983361	0.039406284	0.135934119	0	0	0.036414867
TCGA-55-8204	0.06026907	0	0.27441483	0.06445935	0	0.017302242	0.141741644	0	0	0	0	0.02254937	0.0010268	0.03019265	0.116056047	0.025326108	0.09118502	0	0.12870674	0	0	0.026770157
TCGA-55-8205	0.080546753	0	0.03535587	0.18041222	0	0.052959391	0.111688494	0.006693801	0	0	0.004750137	0.008305785	0.0167497	0.025620123	0.096586013	0.197064836	0.128641543	0	0.028161982	0	0	0.026463313
TCGA-55-8208	0.040994146	0	0.11862406	0.07999231	0	0.038310213	0.039718726	0.015465588	0.066762169	0	0	0.019291387	0	0.218046913	0.115160206	0.141295847	0.011706834	0	0.065326985	0	0	0.029304613
TCGA-55-8299	0.065116761	0	0	0.15848338	0	0	0.076033323	0.057510363	0.030046603	0	0.067124766	0.01398664	0.000962	0.032586082	0.07840858	0.06430977	0	0.181980652	0.160044198	0	0	0.013406881
TCGA-55-8302	0.109697372	0	0.05141792	0.12538124	0	0	0.031317232	0.05068316	0.013996171	0	0	0.042807781	0	0.099278618	0.151799941	0.142628573	0.064636248	0	0.099365126	0	0	0.016990617
TCGA-55-8505	0.116740815	0	0.1576699	0	0	0.018462409	0.042629634	0.0003124	0	0	0.024831904	0	0	0.41595189	0.016804534	0.074817826	0	0	0.130740635	0	0	0.001038048
TCGA-55-8511	0.163881569	0	0.08860357	0.13373528	0	0.067336943	0.047885188	0.040354629	0.03506173	0.009572313	0	0.021318582	0	0.097047901	0.119845297	0.139451968	0.00979329	0	0.024548986	0	0	0.001562759
TCGA-55-8620	0.085070402	0	0.1857411	0.1377335	0	0	0.024929453	0.031331324	0.018182203	0.00188243	0	0.039156367	0	0.195393723	0.103606098	0.090901158	0.018396144	0	0.067676098	0	0	0
TCGA-55-A48Y	0.123646575	0	0.18319541	0.03026738	0	0.072723845	0.029222426	0	0.048489886	0.0147608	0	0.031467622	0	0.312329263	0.012385946	0.066356595	0	0.012096722	0.05122577	0	0	0.011831763
TCGA-55-A48Z	0.103881987	0	0.2262529	0.0530654	0	0.038895229	0.004370141	0.012324645	0.051429955	0	0	0.025788375	0	0.232782937	0.060188848	0.112345352	0.005017745	0	0.052656849	0	0	0.020999638
TCGA-55-A490	0.054555442	0	0.07035169	0.17384155	0	0.020779935	0.015536326	0	0.132606503	0	0	0.010409388	0.0147947	0.216098597	0.011879679	0.149306451	0.093788115	0	0.000552481	0	0	0.035499151
TCGA-55-A491	0.104075513	0	0.18162801	0.09496517	0	0.017969194	0	0.00888421	0.09682721	0	0.020621108	0	0.0148554	0.234133215	0.114360218	0.05304132	0.033004154	0	0.0256353	0	0	0
TCGA-55-A4DF	0	0	0.05900311	0.1158094	0	0.188896512	0.018148384	0.039224603	0	0	0.020163554	0	0	0.215568121	0.181405898	0.089339127	0.018410628	0	0.031164705	0	0	0.022865962
TCGA-62-8394	0	0.022527599	0	0.06118563	0	0.03951892	0	0.075710553	0.102448668	0.002240639	0.010074102	0	0	0.318521875	0.117096989	0.118359584	0	0.068449574	0	0.044061694	0.01980417	0
TCGA-62-8398	0.121451256	0	0.02317503	0.04954388	0	0.150623233	0.063642467	0.015767449	0.072582509	0	0.047602621	0	0	0.206256055	0.08595089	0.077342027	0.045857358	0	0.022512028	0	0	0.017693204
TCGA-64-1676	0.051526467	0	0.09217363	0.16591029	0	0.031376149	0.03902403	0	0	0.040712111	0	0.032160432	0.0673772	0.068142688	0.050766004	0.211108444	0.102685282	0	0.045331245	0	0	0.001706041
TCGA-64-1677	0.085867264	0	0.20579392	0.0955816	0	0.083664171	0.138869119	0.050755956	0	0.002713493	0.027605738	0	0	0.087084443	0.130695149	0.040353419	0.009182814	0.004484189	0.026262529	0	0	0.011086194
TCGA-64-1679	0.059808539	0	0.0659667	0	0	0.078100396	0	0	0.030124574	0	0.032987269	0	0	0.356032156	0.036439754	0.085465644	0.133043709	0.002631576	0.030200653	0.035640905	0	0.053558121
TCGA-64-5778	0	0.062759603	0.06111871	0.22844562	0	0.026962639	0.055079699	0.067366412	0	0.089688011	0	0.036656817	0	0.081371695	0.146897138	0.094635572	0.009252052	0	0.039766033	0	0	0
TCGA-64-5781	0.034999594	0	0.12405492	0.34315178	0	0	0.206265048	0.036900922	0.02052539	0	0.005177047	0.040633847	0	0.071609217	0.030103218	0.039555105	0.015222815	0.013908772	0.017656698	0	0	0.000235627
TCGA-64-5815	0.028971952	0	0.04547828	0.01439027	0	0.11572806	0.024704681	0.020525638	0.031555122	0	0.014348508	0	0	0.340030378	0.00893416	0.113934239	0.052219569	0.097789031	0.072295922	0	0	0.019094191
TCGA-67-3771	0	0	0.1789378	0.1166961	0	0.047440086	0	0	0.05127589	0	0	0.00239114	0	0.194686594	0.143347122	0.182103217	0.063703873	0	0.015642246	0	0	0.003775933
TCGA-67-3773	0.064337832	0	0.03817203	0.07341023	0	0.16946497	0	0.060013752	0	0	0	0.024147613	0	0	0.062994332	0.144201914	0.137852493	0	0.201171985	0	0	0.024232851
TCGA-69-7761	0.013094718	0	0.19167575	0.11670866	0	0.203230491	0.045697383	0.01043866	0.001312634	0	0	0.004429111	0	0.126684028	0.088775682	0.058561011	0.033016625	0	0.080055616	0	0	0.026319626
TCGA-69-7765	0.045941593	0	0.18038359	0.07838951	0	0.077766744	0.058738273	0	0.003604006	0	0.096071581	0	0	0.096365522	0.062742694	0.108357929	0.149433841	0	0	0.007089312	0	0.035115403
TCGA-69-7974	0.049974239	0	0	0.08368209	0	0.090967764	0.113714351	0.010544084	0	0	0	0.049190905	0.0039795	0	0.053284523	0.259527681	0.19484076	0.024485151	0.041164844	0	0	0.02464411
TCGA-69-7978	0.043053015	0	0.07184358	0.14832428	0	0.118139736	0.036564239	0	0.035976067	0	0	0.029516567	0	0.061789693	0.078948521	0.158817165	0.144201826	0	0.063295325	0	0	0.009529986
TCGA-69-7980	0.188762189	0	0	0.14944838	0	0.09969019	0	0.044295543	0.060647333	0	0	0.019488811	0.0065617	0.057567768	0.11465125	0.158914238	0.027583922	0	0.069057116	0	0.00333156	0
TCGA-69-8255	0.120173564	0	0.07208261	0.25446925	0	0	0.212008448	0.017855259	0.026589187	0	0.060146617	0.010333006	0	0.03976451	0.047051935	0.06125767	0.013212463	0	0.05935224	0	0	0.005703247
TCGA-69-8453	0	0.022545071	0	0.08445287	0	0.042433936	0	0.073950642	0.058651512	0.010421242	0.025688176	0	0	0.348414655	0.078796711	0.124094867	0.045207415	0	0.047900897	0.006326733	0	0.03111527
TCGA-73-4658	0.033507352	0	0	0.0885605	0	0.078463614	0.099934671	0.058111855	0	0.012954647	0	0	0.0137141	0	0.062227525	0.130748013	0.375016198	0.00382168	0	0.023720453	0	0.019219376
TCGA-73-4666	0.050087991	0	0	0.21812118	0	0	0.192711837	0.027048731	0	0	0.043881041	0.022600117	0	0.039234438	0.07529348	0.173062926	0.051117087	0	0.085514296	0	0	0.021326879
TCGA-73-4668	0.057008003	0	0.21776871	0.05442717	0	0.097575335	0.034183757	0	0.007408656	0	0.010617224	0	0	0.225580283	0.008205399	0.089778614	0.010727886	0.034459893	0.136873315	0	0	0.01538576
TCGA-73-4675	0.137703774	0	0.00554776	0.03268255	0	0.056177832	0	0.0037456	0.083607427	0	0	0.033552914	0	0.315050131	0	0.150164757	0.078396328	0.033117333	0.051326866	0	0	0.018926732
TCGA-73-4676	0	0	0.01076486	0.13587087	0	0.011339733	0.131396218	0.004826523	0	0	0	0	0.0469454	0.061741796	0	0.213213117	0.069311284	0.065427786	0.223755478	0	0	0.025406901
TCGA-73-7499	0.067515039	0	0.11938573	0.21150138	0	0	0.091757247	0.015018878	0.062938836	0.039433199	0	0	0	0.068039095	0.140353808	0.076054822	0.060841096	0	0.042458895	0	0	0.004701977
TCGA-75-5122	0	0	0.01113867	0.348412	0	0.016390868	0.045474692	0.051325302	0	0	0	0.028825512	0.026922	0	0.045976825	0.276020729	0.123886828	0	0	0.006672082	0	0.018954511
TCGA-75-5125	0.082840694	0	0.02280371	0.1002955	0	0.06769277	0.058040904	0.073091705	0	0.007457143	0	0.046277996	0.0051652	0.024782264	0.119112539	0.175288294	0.076515291	0.021921604	0.102435155	0	0	0.016279234
TCGA-75-6205	0	0.050121326	0	0.1265114	0	0.162165794	0.005035798	0.003951176	0.037845914	0	0.025034165	0	0	0.091831228	0.106489151	0.252828203	0.031314516	0	0.07477591	0.00683283	0	0.025262591
TCGA-78-7154	0.165872584	0	0	0.10126573	0	0.156980874	0	0.03868423	0.068589087	0	0	0.026964087	0.0235388	0.004398608	0.120425782	0.153163036	0.006313077	0	0.133804145	0	0	0
TCGA-78-7160	0	0	0.02210286	0.26662202	0	0.065666204	0.040880091	4.07E-05	0.063548341	0	0	0.024693829	0.0207455	0.076543856	0.104224027	0.17501685	0.053366815	0	0.083716051	0	0.00283281	0
TCGA-78-7535	0.054102473	0	0.02494508	0.05849272	0	0.031649344	0	0	0.090100194	0	0.029586028	0	0	0.199640932	0.012192327	0.087541778	0.35360214	0	0	0.050045063	0	0.00810192
TCGA-78-7536	0.078303824	0	0.06151438	0.13795682	0	0.04275746	0.114037743	0.051574982	0	0.000786776	0	0.054202135	0.0072028	0.015389688	0.119973122	0.166550269	0.04133627	0	0.082792903	0	0	0.025620867
TCGA-83-5908	0.08438173	0	0	0.24954306	0	0.09978167	0.158111828	0.056029039	0	0.033259258	0.035174936	0	0	0.031358369	0.09655107	0.034002105	0.087115144	0	0.026799594	0	0	0.007892196
TCGA-86-6562	0.098844043	0	0.0971946	0	0	0.139210222	0.011455695	0	0.027060875	0	0.005986358	0	0	0.166118912	0.034723098	0.14500034	0.021872815	0	0.20719349	0	0	0.045339554
TCGA-86-7701	0.119458328	0	0.27004596	0.07314337	0	0.199443868	0.047245727	0.004768242	0.042065884	0.00894301	0.023917984	0	0	0.044229781	0.046281733	0.040943298	0.03252504	0.009965265	0.027738894	0	0	0.009283618
TCGA-86-7954	0.011133679	0	0	0.05513826	0	0.163013629	0	0.081303606	0.069143647	0.005671549	0	0.026655943	0.0004542	0.027638835	0.192938002	0.202310218	0.016867786	0.045382296	0.080652385	0	0	0.021696001
TCGA-86-8279	0.10875391	0	0.01105921	0	0	0.037514562	0	0.032699498	0.050123043	0	0	0.043108848	0	0.348322311	0.032424242	0.086616847	0.24170339	0.007674141	0	0	0	0
TCGA-86-8358	0.006497898	0	0.11955543	0.05385408	0	0	0.058432579	0.050670225	0	0.053206019	0	0.07330483	0	0.28861988	0.091989732	0.16217448	0.016035337	0	0.009096755	0	0.01656275	0
TCGA-86-8359	0.043904603	0	0.06635684	0.36136506	0	0.042963489	0.094365509	0.064204083	0.101134494	0	0	0	0	0.145564954	0.041751094	0.009572939	0	0	0	0	0	0.028816936
TCGA-86-8585	0.132234186	0	0.01702906	0.13714859	0	0.018091587	0.0693265	0.014704134	0	0	0	0.051458791	0	0.309131733	0.082892759	0.095295746	0.017511085	0	0.055175831	0	0	0
TCGA-91-6830	0.085824194	0	0.03675554	0.08477958	0	0.136204903	0.020269152	0	0	0	0	0.034323985	0	0.168704477	0.11066212	0.099915641	0	0.019882229	0.170032365	0	0	0.032645818
TCGA-91-6848	0.06471897	0	0.07577069	0.09252825	0	0.163336592	0.08171557	0.00518585	0	0	0.008272826	0.039569275	0	0.189136896	0.093798343	0.136208329	0.029014848	0	0.013332236	0	0	0.007411326
TCGA-91-A4BC	0.047928459	0	0.09776093	0.34532276	0	0	0.22135904	0.037067001	0	0.004208857	0.054032513	0	0	0.049425036	0.029123723	0.055079231	0.010231862	0	0.04846059	0	0	0
TCGA-93-A4JQ	0.123708698	0.055574123	0	0.06475036	0	0.262102645	0	0.043119598	0.069774688	0	0	0.013484688	0	0.173351517	0.098183303	0.089730429	0	0.006219949	0	0	0	0
TCGA-95-7562	0.146084565	0	0.05404733	0.06542104	0	0.080232037	0.033172849	0	0.055635067	0	0	0.01264423	0	0.185885254	0.097301705	0.155437587	0	0	0.081651658	0	0	0.032486683
TCGA-95-7944	0.073711362	0	0	0.24136361	0	0	0.198626074	0.029118976	0.014885451	0	0.075245808	0.052147052	0	0.094067169	0.108894607	0.063884243	0.002500128	0	0.04555552	0	0	0
TCGA-95-A4VN	3.30E-05	0	0	0.1832891	0	0.068293576	0.081826272	0.099141979	0	0	0.031901239	0.016512658	0	0.136517517	0.091529205	0.196226936	0.01717952	0.012663226	0.041585832	0	0	0.023299939
TCGA-97-8174	0.085072741	0	0.1021671	0.05018859	0	0.282333626	0	0.020831905	0	0	0.024041588	0	0.0051602	0	0.102154273	0.190875935	0.023241779	0.015051151	0.094794198	0	0	0.004086884
TCGA-97-8175	0.049979627	0	0	0.00748088	0	0.108622266	0	0.010854892	0.054700607	0	0	0.018334212	0	0.129729981	0.02997637	0.14659701	0.328766205	0.007922379	0.012817953	0	0	0.094217618
TCGA-97-8176	0.186176782	0	0.02778227	0.07122078	0	0	0.082734668	0	0	0	0.045827259	0	0.0123996	0.135709995	0.010843601	0.240152061	0.045972187	0	0.109468679	0	0	0.031712138
TCGA-97-8547	0.057928622	0	0	0	0	0.039832385	0	0.022274552	0.042544828	0.000512571	0	0.030722071	0	0.518871573	0	0.104990315	0.031874289	0.082930232	0.049352193	0	0	0.018166368
TCGA-99-8025	0.130970309	0	0.1046965	0.01158886	0	0.132033147	0	0.021401303	0.062950974	0	0	0.009557797	0	0.249073565	0.073901987	0.150728233	0	0	0.044657606	0	0	0.008439716
TCGA-L9-A444	0.020068047	0	0.05468334	0.24910974	0	0.108850401	0.119295881	0.026386257	0	0	0.013588293	0.049555399	0	0.07446792	0.103369343	0.08076195	0.019491955	0	0.061679646	0	0	0.018691834
TCGA-L9-A743	0.086123664	0	0.05877745	0.10606405	0	0.126446581	0.094696473	0.040018576	0.019340808	0	0	0	0	0.098672758	0.04362761	0.095522019	0.050472569	0.058902078	0.070282425	0	0	0.051052939
TCGA-L9-A8F4	0.051670871	0	0.147504	0.15314771	0	0	0.080733849	0.023834979	0.033955843	0.022567996	0	0.038294552	0	0.181651817	0.080457604	0.106542868	0.023687112	0	0.055950797	0	0	0
TCGA-MN-A4N1	0.186671206	0	0.00054081	0	0	0.059632921	0	0.053008467	0.083677414	0	0	0.04248168	0	0.402267494	0	0.063494093	0.057960326	0.044842306	0.005423286	0	0	0
TCGA-MN-A4N4	0.111517647	0	0.14659075	0.06002056	0	0	0.022640251	0	0.018244723	0.001960719	0	0.001481947	0	0.444110836	0.028671741	0.086389273	0	0	0.075509914	0	0	0.002861635
TCGA-MN-A4N5	0	0	0	0.16072111	0	0.006766991	0.008766037	0.146292272	0	0	0	0.205815654	0	0.147134439	0.113458196	0.076019881	0.004321312	0	0.117811394	0	0	0.012892712
TCGA-MP-A4SV	0.027743026	0	0.10538752	0.13129371	0	0.188634204	0.053504747	0.019961339	0.058233863	0	0.027131246	0.008012942	0	0.129906891	0.081706325	0.078460397	0.00475485	0.006476405	0.074336765	0	0	0.004455769
TCGA-MP-A4SW	0.141685974	0	0.17354109	0.0586272	0	0.141610758	0	0.01704346	0.077337603	0	0	0.020882968	0	0.126220711	0.011885282	0.136822357	0.03551468	0.007732173	0.040181435	0	0	0.01091431

TCGA-MP-A4T7	0.112626001	0	0.01624145	0.08722725	0	0.151062814	0	0.095316276	0.022211299	0	0.009013776	0.023004062	0	0.064259058	0.010162512	0.179331822	0.010531967	0.148665648	0.038879454	0	0	0.031466608
TCGA-MP-A4T8	0.125019386	0	0.06359234	0.00832675	0	0	0	0.031384714	0.08531578	0.003178226	0	0.01165232	0	0.426665486	0	0.094353561	0	0.149385986	0.001125456	0	0	0
TCGA-MP-A4TA	0.109490715	0	0.0973033	0.12995452	0	0.107352395	0.054708789	0.076048457	0.012521924	0	0.004732715	0.04370652	0	0.154424008	0.046043808	0.077335678	0	0.045387216	0.032147256	0	0	0.0088427
TCGA-MP-A4TC	0.090229151	0	0.02057035	0.07548265	0	0.064827641	0.043526138	0.016823162	0.031411708	0.007561285	0.058207714	0	0	0.281606589	0.081520235	0.138098954	0.036773065	0	0	0.032712689	0	0.020648669
TCGA-MP-A4TI	0.039616167	0	0.00879779	0.08638873	0	0.096424654	0.06431765	0.045582728	0.033589839	0	0.015430522	0.011660696	0	0.198015554	0.118038414	0.151312544	0.015709637	0.007126826	0.086963969	0	0	0.021024287
TCGA-MP-A4TJ	0.050945815	0	0.09483669	0.14396474	0	0.187314925	0.114396077	0	0.015815106	0	0.008714825	0	0	0.104208374	0.091705462	0.081882836	0.043517914	0	0.037072868	0	0	0.025624369
TCGA-MP-A4TK	0.096539522	0	0.15257836	0.03583498	0	0.093044643	0.010549908	0.007238131	0.012527751	0.00452629	0	0	0	0.321959686	0.037981761	0.139538031	0.049018407	0	0.028991722	0	0	0.009670803
TCGA-NJ-A4YP	0.030351575	0	0.09493148	0.06928768	0	0.18624856	0.033229639	0.05108352	0.007798898	0	0.022814392	0.020041392	0	0.150579371	0.039288407	0.115865823	0	0.062407573	0.091249681	0	0	0.024822011
TCGA-NJ-A55R	0.158498323	0	0.33325091	0.03169454	0	0.050714474	0	0.003350176	0.030267937	0	0	0.019424268	0	0.216308678	0.000370909	0.039771036	0	0	0.115763796	0	0	0.000584952
TCGA-S2-AA1A	0.106125306	0	0.18933368	0.06762687	0	0.210060774	0	0.040329809	0.030176249	0	0	0.046068988	0	0.099119366	0.038801843	0.039009347	0.031953172	0	0.101394596	0	0	0
